# Mismatch repair protein MLH1 suppresses replicative stress in BRCA2-deficient breast tumors

**DOI:** 10.1172/JCI173718

**Published:** 2024-01-25

**Authors:** Satheesh K. Sengodan, Xiaoju Hu, Vaishnavi Peddibhotla, Kuppusamy Balamurugan, Alexander Y. Mitrophanov, Lois McKennett, Suhas S. Kharat, Rahul Sanawar, Vinod Kumar Singh, Mary E. Albaugh, Sandra S. Burkett, Yongmei Zhao, Bao Tran, Tyler Malys, Esta Sterneck, Subhajyoti De, Shyam K. Sharan

**Affiliations:** 1 Mouse Cancer Genetics Program, Center for Cancer Research, National Cancer Institute, Frederick, Maryland USA.; 2 Rutgers Cancer Institute of New Jersey, Rutgers, the State University of New Jersey, New Brunswick, New Jersey, USA.; 3 Laboratory of Cell and Developmental Signaling, Center for Cancer Research, National Cancer Institute, Frederick, Maryland, USA.; 4 Statistical Consulting and Scientific Programming, Frederick National Laboratory for Cancer Research, NIH, Frederick, Maryland, USA.; 5 Laboratory Animal Sciences Program, Leidos Biomedical Research Inc., Frederick National Laboratory for Cancer Research, Frederick, Maryland, USA.; 6 Department of Cell Biology, Albert Einstein College of Medicine, Bronx, New York, USA.; 7 NCI Advanced Technology Research Facility, Center for Cancer Research, National Cancer Institute, Frederick, Maryland, USA.

**Keywords:** Genetics, Breast cancer, DNA repair, Genetic instability

## Abstract

Loss of BRCA2 (breast cancer 2) is lethal for normal cells. Yet it remains poorly understood how, in *BRCA2* mutation carriers, cells undergoing loss of heterozygosity overcome the lethality and undergo tissue-specific neoplastic transformation. Here, we identified mismatch repair gene mutL homolog 1 (*MLH1*) as a genetic interactor of *BRCA2* whose overexpression supports the viability of *Brca2-*null cells. Mechanistically, we showed that MLH1 interacts with Flap endonuclease 1 (FEN1) and competes to process the RNA flaps of Okazaki fragments. Together, they restrained the DNA2 nuclease activity on the reversed forks of lagging strands, leading to replication fork (RF) stability in BRCA2-deficient cells. In these cells, MLH1 also attenuated R-loops, allowing the progression of stable RFs, which suppressed genomic instability and supported cell viability. We demonstrated the significance of their genetic interaction by the lethality of *Brca2*-mutant mice and inhibition of *Brca2*-deficient tumor growth in mice by *Mlh1* loss. Furthermore, we described estrogen as inducing MLH1 expression through estrogen receptor α (ERα), which might explain why the majority of BRCA2 mutation carriers develop ER-positive breast cancer. Taken together, our findings reveal a role of MLH1 in relieving replicative stress and show how it may contribute to the establishment of BRCA2-deficient breast tumors.

## Abstract 2

The mismatch repair protein MLH1, a direct target gene of ER-alpha, overcomes cell lethality due to BRCA2 loss by relieving replicative stress.

## Graphical abstract

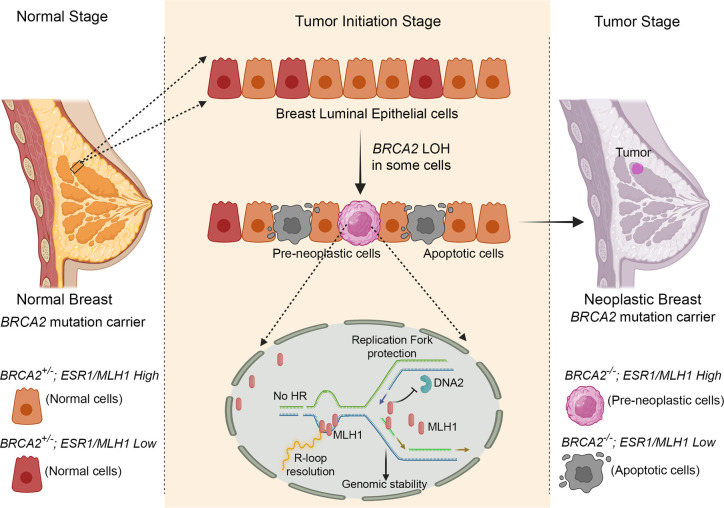

## Introduction

Some of the well-established breast and ovarian cancer susceptibility genes, such as breast cancer 1 (*BRCA1*), *BRCA2*, and *PALB2*, encode proteins that play a key role in repairing double-strand breaks (DSBs) by homologous recombination (HR) (1). BRCA2 is also required for the protection of replication forks (RFs) during replicative stress and resolution of R-loops formed during transcription (2–4). Both fork protection and R-loop resolution have been shown to promote genomic stability in multiple model systems (5, 6). Thus, BRCA2 functions as a tumor suppressor by maintaining genomic stability. Because of this vital function, BRCA2 is essential for viability of normal cells, including embryonic stem cells (7). Its loss results in embryonic lethality in mice (8, 9). Therefore, it is quite intriguing how some BRCA2-deficient normal cells overcome the crisis of BRCA2 loss and initiate their transformation into preneoplastic/neoplastic cells. Haploinsufficiency in normal mammary gland has also been proposed as a driver for BRCA-associated tumor development (10). But this possibility is called into question by the fact that genetically engineered *Brca2* heterozygous mice are not tumor prone (11). Previous reports have suggested that BRCA2 loss of heterozygosity (LOH) may not be the earliest event in the evolution of BRCA2-null preneoplastic cells (12, 13). We hypothesize that some normal cells of the mutation carriers acquire competence to overcome the BRCA2 loss–induced genomic threats and growth arrest before undergoing LOH. This is supported by the cooccurrence of *TP53* mutations in *BRCA*-mutated cancer (14). We have identified several genetic interactors that can support the viability of BRCA2-deficient cells. Loss of *Ptip*, *Tet2*, and heterozygosity of *Parp1* can support viability of *Brca2-*null mouse embryonic stem cells (mESCs) by conferring genomic stability (15–17).

We have previously reported that transient inhibition of MRE11 nuclease activity by mirin prior to the deletion of the conditional allele rescues the lethality of *Brca2-*null mESCs (15). Here, we sought to investigate the molecular mechanisms underlining the survival of these cells even after the inhibitor is removed. By RNA-Seq, we found mutL homolog 1 (*Mlh1*) to be overexpressed in these *Brca2^KO/KO^* clones. We found MLH1 to confer survival advantage to the rescued cells. MLH1 is a mismatch repair (MMR) protein, which forms a heterodimeric complex with PMS2 (MutLα) and MLH3 (MutLγ) to initiate the repair of mismatches on one of the DNA strands. However, MMR-independent roles of MLH1 are also well documented. For example, MLH1 along with MLH3 has been shown to play an important role in Holliday junction resolution during meiosis (18) and DNA repeat expansion (19). Here, we show the role of *MLH1* as a genetic interactor of *BRCA2* by protecting the stalled RFs from DNA2-mediated degradation and resolving R-loop in the absence of BRCA2. Our findings reveal that upregulation of MLH1 can restore genomic stability and support cell viability in BRCA2-deficient cells. Similarly, loss of MLH1 reduces growth of BRCA2-deficient tumors and causes lethality of *Brca2* mutant mice expressing a hypomorphic allele. Furthermore, we show that estrogen receptor α (ERα) induces MLH1 expression, which explains why BRCA2-associated tumors are mostly ER positive, which is a major subtype of breast cancer cases.

## Results

### MLH1 rescues lethality of Brca2^KO/KO^ ES cells.

**Figure 1:**
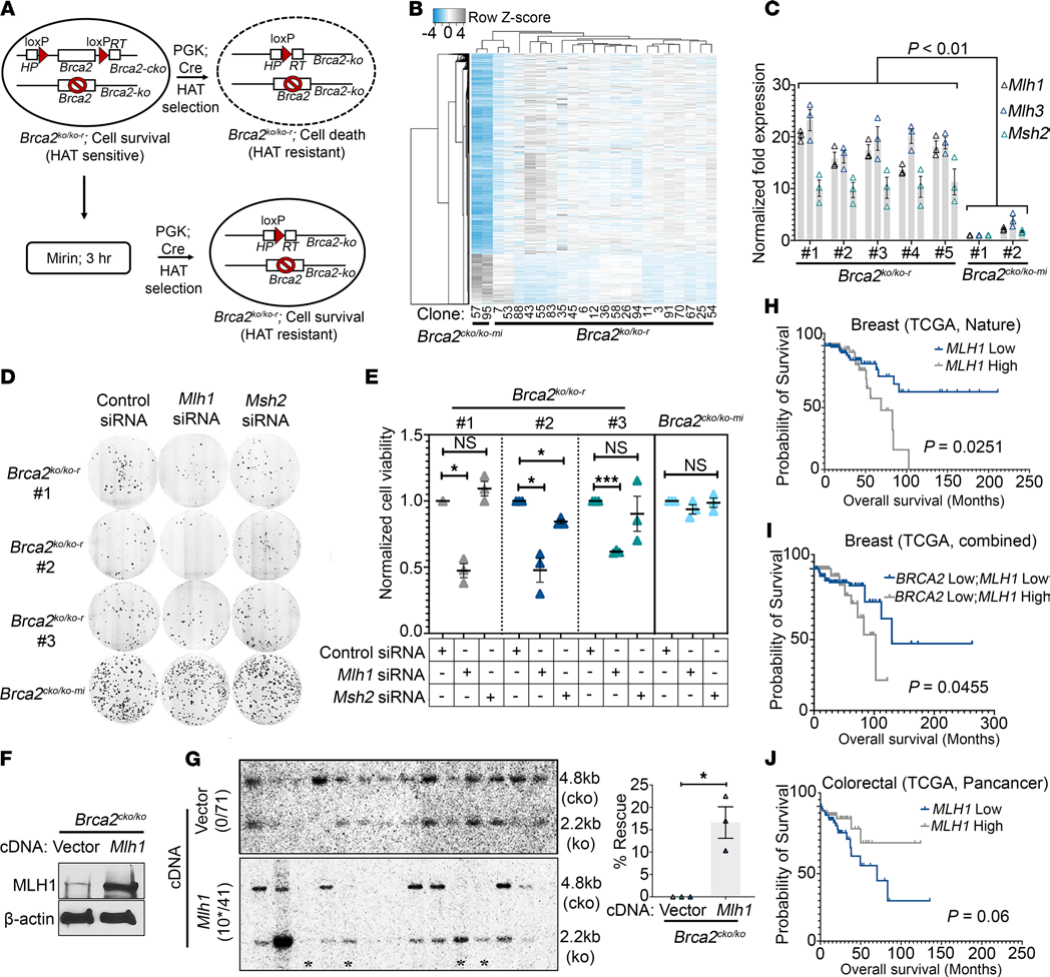
MLH1 rescues and promotes survival of BRCA2-deficient cells. (**A**) Schematic representation of experimental approach to generating *Brca2^KO/KO-r^* mESC clones from (*Brca2^cKO/KO^*) mESCs after mirin (10 μM for 3 hours) pretreatment. Conditional allele was deleted in DMSO or mirin-treated *Brca2^cKO/KO^* cells by Cre expression followed by selection in HAT media. (**B**) Heatmap showing mRNA expression of upregulated and downregulated genes in *Brca2^KO/KO-r^* (*n* = 22) and *Brca2^cKO/KO-mi^* clones (*n* = 2). (**C**) RT-qPCR analysis of *Mlh1*, *Msh2*, and *Mlh3* in *Brca2^KO/KO-r^* and *Brca2^cKO/KO-mi^* clones (*n* = 3 biological replicates). Independent clones are marked by number symbols. (**D**) Representative images showing colony formation upon *Mlh1* and *Msh2* silencing in *Brca2^KO/KO-r^* (*n* = 3 clones) and *Brca2^cKO/KO-mi^* clones (*n* = 1 clone). (**E**) Quantitation of **D**, showing the normalized cell viability in *Brca2^KO/KO-r^* and *Brca2^cKO/KO-mi^* clones (*n* = 3 biological replicates). (**F**) Immunoblot showing overexpression of MLH1 in *Brca2^cKO/KO^* mESCs. (**G**) Southern blot showing rescue of *Brca2^KO/KO^* clones in control vector or MLH1-overexpressing *Brca2^cKO/KO^* mESCs (left panel). Asterisks indicate *Brca2^KO/KO^* clones. Quantitation of percentage rescue in vector or *Mlh1*-overexpressing *Brca2^cKO/KO^* mESCs (*n* = 3 biological replicates) (right panel). (**H**) Kaplan-Meier analysis showing survival status of patients from *MLH1*-high (*n* = 111) versus *MLH1*-low (*n* = 80) breast cancer patient samples obtained from the TCGA Nature breast cancer data set. (**I**) Kaplan-Meier analysis showing the survival status of patients from *BRCA2*-low; *MLH1*-high (*n* = 100) versus *BRCA2*-low; *MLH1* low (*n* = 134) breast cancer patient samples pooled from TCGA breast cancer data sets (TCGA Nature, Cell, Firehose Legacy). (**J**) Kaplan-Meier analysis showing survival status of patients from *MLH1*-high (*n* = 68) versus *MLH1*-low (*n* = 74) colorectal cancer patient samples obtained from TCGA Pancancer data set. Data were analyzed using unpaired, 2-tailed Student’s *t* test (**G**) with Holm-Šidák multiple-comparison test (**C**), Holm multiple-comparison test (**E**), and log-rank (Mantel-Cox) test (**H**–**J**). *P < 0.05; ***P < 0.001.

We have previously shown that transient treatment of PL2F7 mESCs carrying a null (*KO*) and conditional (*cKO*) allele of *Brca2* (hereafter referred to as *Brca2^cKO/KO^)* with the MRE11 inhibitor mirin can rescue the lethality of *Brca2*-null mESCs (hereafter referred to as *Brca2^KO/KO-r^*) (15). However, the molecular mechanism associated with the rescue and survival of *Brca2^KO/KO-r^* mESCs was poorly understood. To elucidate the mechanism, we generated independent *Brca2^KO/KO-r^* mESCs by pretreating the *Brca2^cKO/KO^* mESCs transiently with mirin (Figure 1A). Recombinant clones were selected in hypoxanthine-aminopterin-thymidine (HAT) media due to the generation of a function *HPRT* minigene as a result of the recombination between the 2 *loxP* sites in the *Brca2^cKO^* allele (Figure 1A). HAT-resistant colonies were genotyped by Southern blotting, and 51% of these clones were confirmed to be *Brca2^KO/KO-r^* clones (Supplemental Figure 1A; supplemental material available online with this article; https://doi.org/10.1172/JCI173718DS1).

To understand the mechanism of viability of these cells, we performed RNA-Seq analysis of 19 independently generated *Brca2^KO/KO-r^* clones. As BRCA2-proficient control cells, we used 2 clones that were also pretreated with mirin and selected in HAT media, but they retained the conditional allele of *Brca2*. These clones will hereafter be referred to as *Brca2^cKO/KO-mi^*. Loss of the conditional allele in *Brca2^KO/KO-r^* clones was confirmed by integrative genomic viewer (IGV), which showed a loss of the 5′ end of exon 11 of the *cKO* allele of *Brca2* (Supplemental Figure 1B). We did not observe any change in the expression levels of MRE11 in the *Brca2^KO/KO-r^* clones (Supplemental Figure 1, C and D). The RNA-Seq data revealed upregulation of many genes in *Brca2^KO/KO-r^* clones and, strikingly, a high level of homogeneity among 19 *Brca2^KO/KO-r^* clones was observed (Figure 1B). Pathway analysis showed significant enrichment of multiple pathways. Among these, Fanconi anemia (FA) and MMR were two of the top enriched DNA repair pathways in all the *Brca2^KO/KO-r^* clones (Supplemental Figure 1E). Because the role of the MMR pathway in complementing BRCA2 function is unknown, we focused on the MMR genes to determine whether there was any functional interaction with BRCA2. The enrichment of MMR genes in *Brca2^KO/KO-r^* clones in comparison with *Brca2^cKO/KO-mi^* is shown in Supplemental Figure 1F. We confirmed the upregulation of MMR genes (*Mlh1*, *Mlh3*, *Msh2*, and *Msh6*) by quantitative reverse-transcription PCR (RT-qPCR) and Western blotting (Figure 1C and Supplemental Figure 1G).

To examine the effect of the MMR genes on the survival of *Brca2^KO/KO-r^* clones, we knocked down *Mlh1*, *Mlh3*, *Pms2*, and *Msh2* in multiple clones. We found that the loss of *Mlh1*, but not *Mlh3*, *Msh2*, or *Pms2*, significantly reduced cell viability in all clones (Figure 1, D and E, and Supplemental Figure 1, H and I). This suggested an important role of MLH1, but not the MMR pathway, in the viability of these cells. To directly test whether MLH1 has a role in the rescue of *Brca2^KO/KO-r^* clones, we knocked down *Mlh1* prior to mirin pretreatment. After deletion of the *cKO* allele of *Brca2*, the level of rescue was analyzed by treating the HAT-resistant clones with or without olaparib (Supplemental Figure 2, A and B). We postulated that if MLH1 is crucial for the rescue of *Brca2^KO/KO-r^* clones, the number of HAT-resistant *Brca2^KO/KO-r^* clones should remain low and there should not be a significant reduction in the cell number after olaparib treatment. In contrast, control siRNA–treated mESCs should show a significant reduction in the number of HAT-resistant colonies after olaparib treatment. While we observed a significant reduction in the colony number (~40%–50% reduction) upon olaparib treatment in control mirin-pretreated mESCs, the difference was minimal in *Mlh1*-silenced mirin-pretreated *Brca2^cKO/KO-mi^* mESCs, as expected (5%–15%) (Supplemental Figure 2C). We further validated the genotype of *Brca2^KO/KO-r^* and *Brca2^cKO/KO-mi^* clones by Southern blotting. We found the rescue rates to be 25% and 7% in control and *Mlh1-*silenced cells, respectively (Supplemental Figure 2D). Interestingly, silencing of *Mlh3* and *Msh2* had no effect on the rescue of *Brca2^KO/KO-r^* clones (Supplemental Figure 2, E and F). To directly test the role of MLH1 in viability of *Brca2^KO/KO^* cells, we overexpressed MLH1 in *Brca2^cKO/KO^* cells and performed the rescue experiment without mirin pretreatment. After deletion of the conditional allele, 15% of MLH1-overexpressing HAT-resistant clones were confirmed to be *Brca2^KO/KO-r^* (Figure 1, F and G). Taken together, these results demonstrate that MLH1 plays an important role in the rescue and survival of *Brca2-*deficient cells.

We next analyzed The Cancer Genome Atlas (TCGA) database to determine the clinical significance of our findings. We found that *MLH1*-low breast cancer patients have a better overall survival than *MLH1*-high breast cancer patients (Figure 1H and Supplemental Figure 3, A and B). Corroborating our findings in mESCs, differential expression of other MMR genes (such as *MLH3*, *MSH2*, and *PMS2*) had no effect on overall survival of breast cancer patients (Supplemental Figure 3, C–E). We next analyzed patient survival with respect to *MLH1* status in *BRCA2*-low breast cancer patient samples. Notably, we found *BRCA2*-low; *MLH1*-low patients showed better overall survival than *BRCA2*-low; *MLH1*-high patients (Figure 1I). Interestingly, the correlation of low levels of *MLH1* to better patient survival was observed only in breast and bladder cancer patients (Supplemental Figure 3, A, B, and K). We found an opposite but substantial correlation in colon cancer, where *MLH1*-low patients had poor prognosis relative to *MLH1*-high patients (Figure 1J and Supplemental Figure 3F). *MLH1* tends to have an insignificant impact on the survival of patients with other cancers (Supplemental Figure, 3 G–J and L–O), supporting a tissue-specific role of MLH1 in BRCA2-associated breast cancer.

### MLH1 promotes RF speed and restart in Brca2^KO/KO-r^ cells.

To investigate how MLH1 supports the viability of *Brca2^KO/KO-r^* cells, we determined whether any of the known functions of BRCA2 (HR, RF protection, and R-loop resolution) were restored by MLH1 overexpression. Because the effect of MLH1 loss on cell viability was observed in multiple rescued clones, in subsequent experiments, we focused on 2 randomly selected *Brca2^KO/KO-r^* clones. First, we examined the effect on HR, which was marked by the recruitment of RAD51 to the irradiation-induced (IR-induced) DSBs. While *Brca2^cKO/KO-mi^* cells showed the presence of RAD51 foci, no foci were observed in *Brca2^KO/KO-r^* cells, suggesting lack of HR restoration in *Brca2^KO/KO-r^* cells (Supplemental Figure 3P).

**Figure 2:**
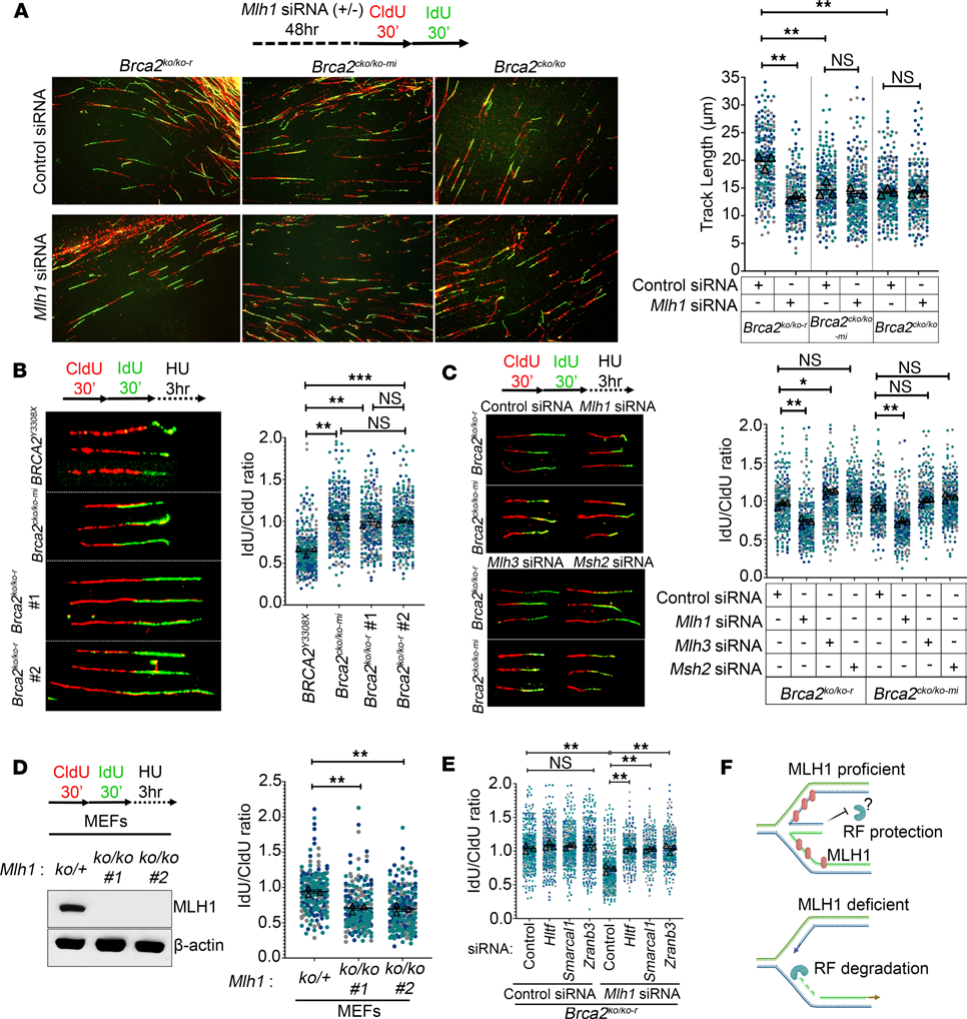
MLH1 promotes RF speed/restart and protects stalled RF in BRCA2-deficient cells. (**A**) Representative images of DNA fibers showing dual color track length in control and *Mlh1*-silenced *Brca2^KO/KO-r^*, *Brca2^cKO/KO-mi^*, and *Brca2^cKO/KO^* clones. Right panel shows quantitation of dual color track length (in μm) using ImageJ. (**B**) Representative images showing IdU/CldU tract ratios of *Brca2^KO/KO-r^* (*n* = 2) and *Brca2^cKO/KO-mi^* (*n* = 1) clones. BRCA2 variant *BRCA2^Y3308X^* (known to have RF degradation) was used as positive control. Replicating DNA was labeled with CldU and IdU, and RFs were stalled by HU treatment. Ratio is approximately 1 when RFs are protected, but when they are not stable, the ratio is significantly less than 1. Right panel shows quantitation of IdU/CldU ratios using ImageJ. (**C**) Representative images showing IdU/CldU tract ratios of control or *Mlh1*- (upper panel) and *Msh2*- or *Mlh3*-silenced (lower panel) *Brca2^KO/KO-r^* and *Brca2^cKO/KO-mi^* clones. Right panel shows quantitation of IdU/CldU tract ratios using ImageJ. Original magnification, ×63. (**D**) Immunoblot showing confirmation of MLH1 knockout in MEFs. β-Actin was used as an endogenous control. Right panel shows quantitation of IdU/CldU tract ratios of BRCA2-proficient *Mlh1*^KO/+^(*n* = 1 MEFs) and *Mlh1^KO/KO^* (*n* = 2 MEFs from independent animals) MEFs. (**E**) Quantitation of IdU/CldU tract ratios of control or *Mlh1*-silenced *Brca2^KO/KO-r^* mESCs under *Hltf*-, *Smarcal1*-, or *Zranb3*-silenced conditions. (**F**) Schematic representation of RF protection or degradation in MLH1-proficient/deficient cells. Each dot represents an individual fiber, and at least 150 fibers pooled from 3 biological replicates (>50 fibers per replicate) were used to generate superplots in **A**–**E**. Each replicate is color coded. Data are represented as means ± SEM. Data were analyzed by unpaired, 2-tailed Student’s *t* test (**A**–**E**) using the mean from 3 biological replicates. *P < 0.05; **P < 0.01; ***P < 0.001.

Next, we investigated DNA replication dynamics in *Brca2^KO/KO-r^* clones, as suppression of replicative stress has been shown to play an important role in the survival of BRCA2-deficient cells (16, 20). We examined RF speed, fork restart, and fork protection in *Brca2^KO/KO-r^* mESCs by DNA fiber assay. Interestingly, RF speed was found to be higher in *Brca2^KO/KO-r^* clones (average tract length, ~17 μm) as compared with the *Brca2^cKO/KO-mi^* (average tract length, ~14 μm) and *Brca2^cKO/KO^* clones (average tract length, ~13 μm) (Figure 2A). We also examined the fork speed using an independent approach by measuring EdU incorporation in replicating cells by immunofluorescence and FACS analysis. We found that the mean EdU intensity was higher in *Brca2^KO/KO-r^* clones in comparison with *Brca2^cKO/KO-mi^* (Supplemental Figure 4, A–C), further confirming an increase in RF speed in *Brca2^KO/KO-r^* cells. We observed that silencing of *Mlh1*, but not *Mlh3* or *Msh*2, reduced the RF speed in *Brca2^KO/KO-r^* clones, whereas no effect was observed in *Brca2^cKO/KO-mi^* or *Brca2^cKO/KO^* clones (Figure 2A and Supplemental Figure 4, D and E). We also detected an enrichment of MLH1 at the active forks of *Brca2^KO/KO-r^* clones by isolation of proteins on nascent DNA (iPOND), further supporting its role in DNA-replication dynamics (Supplemental Figure 4F). This is consistent with a recent study that found MLH1 to be enriched at active RF by a proteomic approach (21). Taken together, these observations suggest that MLH1 overexpression probably allows *Brca2^KO/KO-r^* cells to complete DNA synthesis in S-phase and progress through the cell cycle. DNA under replication in S-phase and mitotic DNA synthesis have been reported to induce cell death in BRCA2-deficient cells (7). Because high fork speed also leads to the generation of replication gaps (Supplemental Figure 5A), some *Brca2^KO/KO-r^* cells with high MLH1 may retain large replication gaps and get arrested in the G_2_/M phase. This may explain why, despite higher replication speed, the rescued *Brca2^KO/KO-r^* mESCs exhibit slower proliferation compared with *Brca2^cKO/KO-mi^* cells.

Since RF speed is affected by MLH1, we tested its effect on RF restart. We found fork restart after hydroxyurea (HU) stalling was higher in *Brca2^KO/KO-r^* clones and varied between 25% and 33% compared with approximately 13% in *Brca2^cKO/KO-mi^* cells (Supplemental Figure 5B). Furthermore, we found that *Mlh1* silencing in *Brca2^KO/KO-r^* cells reduced the fork restart to 20% compared with 33% in control cells, whereas no difference in fork restart was observed in *Brca2^cKO/KO-mi^* clones (Supplemental Figure 5C). We reconfirmed this effect using FACS analysis wherein HU-treated cells were released in EdU media and the percentages of EdU-positive cells were found to be low in *Mlh1*-silenced *Brca2^KO/KO-r^* cells (Supplemental Figure 5D). Overall, our findings suggest that MLH1 is enriched at active RF and its overexpression increases the fork speed and fork restart in the rescued *Brca2^KO/KO-r^* clones.

### MLH1 protects the RFs from DNA2 nuclease in Brca2^KO/KO-r^ cells.

Degradation of stalled RFs by MRE11 is frequently observed in BRCA2-deficient cells (2). Therefore, we next assessed the stability of stalled RFs in the *Brca2^KO/KO-r^* clones. Surprisingly, we found the RFs to be protected (iododeoxyuridine [IdU]/ chlorodeoxyuridine [CldU] ratio >0.9) in these rescued *Brca2^KO/KO-r^* mESCs compared with *BRCA2^Y3308X^*, a *BRCA2*-mutant mESC line that shows RF degradation (15) (IdU/CldU ratio <0.6) (Figure 2B and Supplemental Figure 6A). It is intriguing that despite harboring BRCA2 deficiency and having normal expression levels of MRE11, the stalled RFs were stable. To determine whether MLH1 contributes to RF protection, we silenced *Mlh1* and assessed the fork stability in *Brca2^KO/KO-r^* clones. Knockdown of *Mlh1*, but not *Mlh3* or *Msh2*, resulted in fork degradation in *Brca2^KO/KO-r^* clones (Figure 2C). Surprisingly, *Mlh1* knockdown also resulted in fork degradation in the *Brca2^cKO/KO-mi^* clones (Figure 2C). These effects were confirmed in other independently generated *Brca2^KO/KO-r^* and *Brca2^cKO/KO-mi^* clones (Supplemental Figure 6B). We observed a similar effect of MLH1 loss on fork instability in BRCA2-proficient *Mlh1^KO/KO^* mouse embryonic fibroblasts (MEFs) as well as BRCA2-proficient KB2P1.21R2 mouse mammary tumor cells (Figure 2D and Supplemental Figure 6C).

**Figure 3:**
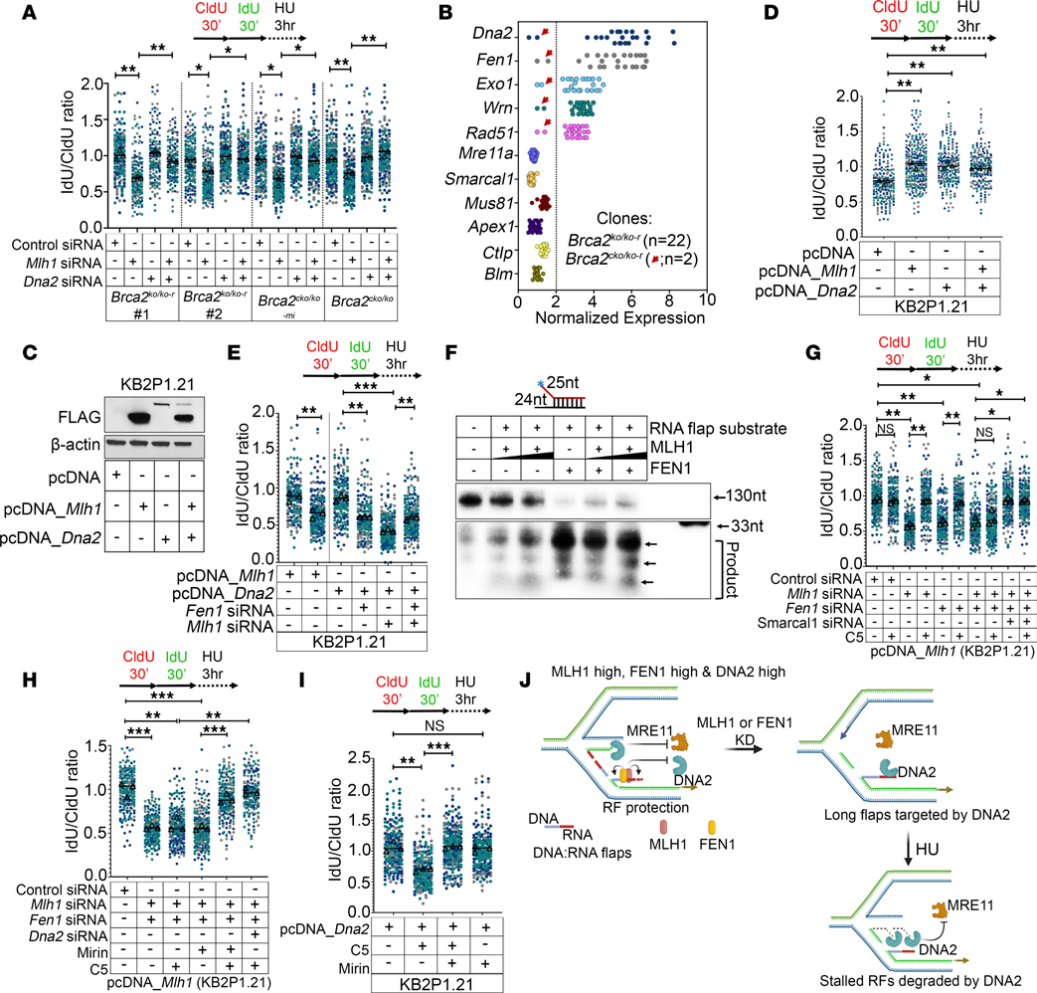
MLH1 protects reversed RFs from DNA2 nuclease. (**A**) Quantitation of IdU/CldU ratios of control, *Mlh1*-, or *Dna2*-silenced or a combination in *Brca2^KO/KO-r^* (*n* = 2), *Brca2^cKO/KO-mi^* (*n* = 1), or *Brca2^cKO/KO^* (*n* = 1) mESCs. (**B**) mRNA level expression status of panel of nucleases in *Brca2^KO/KO-r^*(*n* = 22) and *Brca2^cKO/KO-mi^*(*n* = 2) clones. Each dot in the graph represents an individual *Brca2^KO/KO-r^* or *Brca2^cKO/KO-mi^* clone. Red arrows are used to indicate *Brca2^cKO/KO-mi^* clones. (**C**) Immunoblot showing confirmation of MLH1 and/or DNA2 overexpression in KB2P1.21 cells. (**D**) Quantitation of IdU/CldU ratios of pcDNA_*Mlh1*- or pcDNA_*Dna2*-overexpressing KB2P1.21 cells. (**E**) Quantitation of IdU/CldU ratios of *Dna2-*overexpressing or *Mlh1*-overexpressing KB2P1.21 cells upon control or *Fen1* or *Mlh1* silencing. (**F**) In vitro nuclease assay of RNA flap substrate using pure MLH1 and FEN1 proteins. Processed products are marked by arrows. (**G**) Quantitation of IdU/CldU ratios of pcDNA_*Mlh1*-overexpressing KB2P1.21 cells upon *Mlh1*, *Fen1*, or *Smarcal1* silencing under C5-treated or untreated conditions. (**H**) Quantitation of IdU/CldU ratios of pcDNA_*Mlh1*-overexpressing KB2P1.21 cells upon *Mlh1*, *Fen1*, or *Dna2* silencing under C5- or mirin-treated or untreated conditions. (**I**) Quantitation of IdU/CldU ratios of pcDNA_*Dna2*-overexpressing KB2P1.21 cells upon treating with DNA2 inhibitor C5 or MRE11 inhibitor mirin. (**J**) Schematic representation of model to show RF protection and/or degradation in MLH1 or FEN1-high/low cells under DNA2-high conditions. MLH1/FEN1 binds and processes flaps of Okazaki fragments. MLH1/FEN1 knockdown generates longer Okazaki fragment flaps, which are then bound by DNA2, leading to RF degradation upon fork stalling (with HU). Each dot represents an individual fiber, and at least 150 fibers pooled from 3 biological replicates (>50 fibers per replicate) were used to generate the superplots in **A**, **D**, **E**, **G**, **H**, and **I**. Each replicate is color coded. Data are represented as means ± SEM. Data were analyzed by unpaired, 2-tailed Student’s *t* test (**A**, **D**, **E**, **G**, **H**, and **I**) using the mean from 3 biological replicates. *P < 0.05; **P < 0.01; ***P < 0.001.

One of the protective mechanisms for recovering stalled RFs during replicative stress is RF reversal process, mediated by SNF2 family proteins such as SMARCAL1, ZRANB3, and HLTF (22). However, if the reversed RFs are not protected from the nucleases, it leads to RF degradation. Therefore, we investigated the role of MLH1 in protecting the reversed RFs. Knockdown of fork reversal factors, such as *Smarcal1*, *Hltf*, and *Zranb3*, suppressed fork degradation in *Mlh1*-silenced *Brca2^KO/KO-r^* and *Brca2^cKO/KO-mi^* clones (Figure 2E and Supplemental Figure 6D). Since fork reversal was blocked, the reversed RFs were not readily available for degradation by the nuclease and the RFs remained stable in *Mlh1*-silenced *Brca2^KO/KO^* clones. These results suggest that MLH1 protects the reversed RFs from nucleolytic degradation (Figure 2, E and F, and Supplemental Figure 6D). To understand the mechanism of MLH1-dependent RF protection, we screened a panel of nucleases in human osteosarcoma cell line U2OS by siRNA-mediated knockdown. We found that knockdown of DNA2 but no other nucleases (such as MRE11, EXO1, APE1, CtIP) rescued the MLH1 loss–mediated fork degradation in U2OS (Supplemental Figure 6, E–G). Protection of stalled RFs upon *Mlh1* knockdown was also observed in *Dna2*-silenced *Brca2^KO/KO-r^*, *Brca2^cKO/KO-mi^*, and *Brca2^cKO/KO^* clones (Figure 3A and Supplemental Figure 7, A and B). We observed a similar RF protection by *Dna2* silencing in *Mlh1^KO/KO^* MEFs and *Mlh1*-silenced KB2P1.21R2 cells (Supplemental Figure 7, C–E). These results provide strong evidence to suggest that MLH1 protects the reversed RFs from DNA2 nuclease.

### MLH1 mediates RNA flap processing of Okazaki fragments to promote RF protection from DNA2-mediated overresection.

Interestingly, RNA-Seq analysis of *Brca2^KO/KO-r^* clones showed that *Dna2* and Flap endonuclease 1 (*Fen1*) were also overexpressed along with *Mlh1* (Figure 3B). Regulated resection of RF by DNA2 is crucial for processing of reversed forks and maintaining genomic stability (23). However, overresection of reversed RFs by DNA2 and its association with genomic instability are also reported (24). FEN1 is a structure-specific nuclease that plays a key role in Okazaki fragment maturation (OFM) by processing the short flaps on the lagging strands. However, when short flaps of Okazaki fragments are not processed, longer flaps are generated, which are then bound and targeted by DNA2 via RPA (25). Since *Dna2* and *Fen1* were overexpressed in *Brca2^KO/KO-r^* clones and compete for Okazaki fragment processing, we analyzed their impact on RF stability in the context of MLH1 overexpression. We first examined their functional interaction in an independent system by overexpressing MLH1 and DNA2 in BRCA2-deficient KB2P1.21 cells (Figure 3C). Overexpression of MLH1 independently rescued the fork stability in BRCA2-deficient KB2P1.21 cells (Figure 3D). Surprisingly, *Fen1* knockdown resulted in partial RF degradation in *Mlh1*-overexpressing KB2P1.21 cells (Figure 3E), suggesting that MLH1-mediated fork protection from DNA2 could be influenced by FEN1. Interestingly, overexpression of *Fen1* also rescued fork stability in BRCA2-deficient KB2P1.21 cells (Supplemental Figure 8A). In accordance, silencing of *Mlh1* or *Fen1* resulted in RF degradation in *Brca2^KO/KO-r^* clones (Supplemental Figure 8B). Intriguingly, stable overexpression of *Dna2* also rescued the fork stability in BRCA2-deficient KB2P1.21 cells (Figure 3D). However, we observed severe RF degradation upon *Mlh1* or *Fen1* silencing in *Dna2*-overexpressing KB2P1.21 cells (Figure 3E). Based on these observations we hypothesized that while overexpression of MLH1 and FEN1 protects the RFs from DNA2, overexpression of DNA2 limits MRE11-mediated RF resection, resulting in fork stability in *Brca2^KO/KO-r^* clones and KB2P1.21 cells.

To test this, we investigated how MLH1/FEN1 loss–induced defects in OFM result in RF instability. The role of FEN1 and DNA2 in OFM is well documented (25). Recently, MutLα endonuclease (MLH1/3 complex) was reported to process 1-nucleotide flaps of Okazaki fragments (26). By in vitro binding assay using RNA:DNA duplex structures and pure MLH1 protein, we found that MLH1 binds to the RNA:DNA duplex, which resembles the structure of Okazaki fragments (Supplemental Figure 8C). We also found that MLH1 physically interacts with FEN1 in KB2P1.21 cells (Supplemental Figure 8D). We next performed in vitro nuclease assay using pure MLH1 and FEN1 proteins. We found that MLH1 processes the flaps of RNA strand of RNA:DNA duplex structures, suggesting that the RNA primer of Okazaki fragments can be processed by MLH1 (Figure 3F). While FEN1 processes the flaps of RNA strand more efficiently than MLH1, the degradation product of RNA primer tends to be higher in the presence of MLH1(Figure 3F). Interestingly, increasing the concentration of MLH1 inhibited FEN1-mediated flap processing, suggesting that MLH1 and FEN1 may compete for RNA primer processing of Okazaki fragments (Figure 3F). However, it is unclear whether MLH1 targets all RNA flaps similarly to FEN1. Indeed, secondary structure or specific sequence of RNA flaps could be a critical factor in determining the specificity of MLH1 in processing the RNA flaps. We also found that MLH1 fails to process DNA flap structures, although it inhibits FEN1-mediated DNA flap processing (Supplemental Figure 8, E and F).

In line with the above observation, we found knockdown of *Mlh1* or *Fen1* or a combination of both resulted in fork degradation in *Mlh1*-overexpressing KB2P1.21 cells (Figure 3, G and H, and Supplemental Figure 8, G and H). The DNA2 inhibitor C5 rescued fork degradation associated with *Mlh1* or *Fen1* loss (Figure 3I). However, C5 failed to rescue RF degradation when *Mlh1* and *Fen1* were silenced together. Surprisingly, treating *Mlh1/Fen1*-silenced cells with C5 and mirin rescued RF degradation, suggesting that inhibition of DNA2 nuclease activity allows MRE11 to degrade the RFs in BRCA2-deficient cells (Figure, 3, G and H, and Supplemental Figure 8, G and H). Further, *Smarcal1* knockdown also rescued the fork degradation phenotype associated with *Mlh1* and *Fen1* loss in *Mlh1*-overexpressing KB2P1.21 cells (Figure 3H). A similar phenomenon was also observed in *Dna2*-overexpressing KB2P1.21 cells wherein *Smarcal1* loss rescued the fork degradation phenotype associated with *Mlh1* and *Fen1* loss (Supplemental Figure 8I). It is possible that long unprocessed 5′ flaps of Okazaki fragments are traversed into 3′ flaps, as reported earlier (27), which are then targeted by MRE11 in the absence of DNA2 nuclease activity. We conclude that in the absence of *Mlh1* or *Fen1*, the unprocessed flaps of Okazaki fragments form 5′ overhangs on stalled reversed RFs of lagging strand that are the potential target for DNA2-mediated RF degradation (Figure 3J).These observations also suggest that MLH1 phenocopies FEN1, a synthetic lethal target in BRCA2-deficient cells (28).

Intriguingly, we also observed protection of RFs in *Dna2*-silenced *Brca2^KO/KO-r^* clones (Figure 3A). We predicted that loss of DNA2 will cause accumulation of reversed RFs that could be degraded by MRE11 in the absence of BRCA2. Interestingly, we found that knockdown of *Dna2* also reduced the expression of *Mre11* in control and *Brca2*-silenced *Brca2^cKO/KO^* cells (~60%–80% reduction) (Supplemental Figure 8J). Reverse-phase protein array (RPPA) analysis from TCGA breast cancer data set also showed reduced MRE11 protein expression in *DNA2*-low samples (Supplemental Figure 8K). This may explain why RFs remain protected in *Dna2*-silenced *Brca2^KO/KO-r^* clones. To test this, we analyzed RF stability with or without the DNA2 inhibitor (C5) and the MRE11 inhibitor (mirin) alone or in combination in *Dna2*-overexpressing KB2P1.21 cells. We found RFs to be protected upon *Dna2* overexpression in BRCA2-deficient KB2P1.21 cells. Under these conditions, inhibiting nuclease activity of DNA2 by C5 resulted in fork degradation (Figure 3I). However, RFs remained stable when MRE11 was inhibited by mirin in C5-treated *Dna2*-overexpressing cells (Figure 3I). We conclude from these observations that overexpression of *Dna2* limits MRE11-mediated fork degradation and that when the nuclease activity of DNA2 is inhibited, MRE11 promotes fork degradation in KB2P1.21 cells. Furthermore, our results suggest that MLH1/FEN1 functions in OFM and protects the reversed RFs from DNA2-mediated extensive RF degradation in *Brca2^KO/KO-r^* clones (Figure 3J).

### MLH1 mediates stable RF progression in Brca2^KO/KO-r^ mESCs by attenuating R-loops.

**Figure 4:**
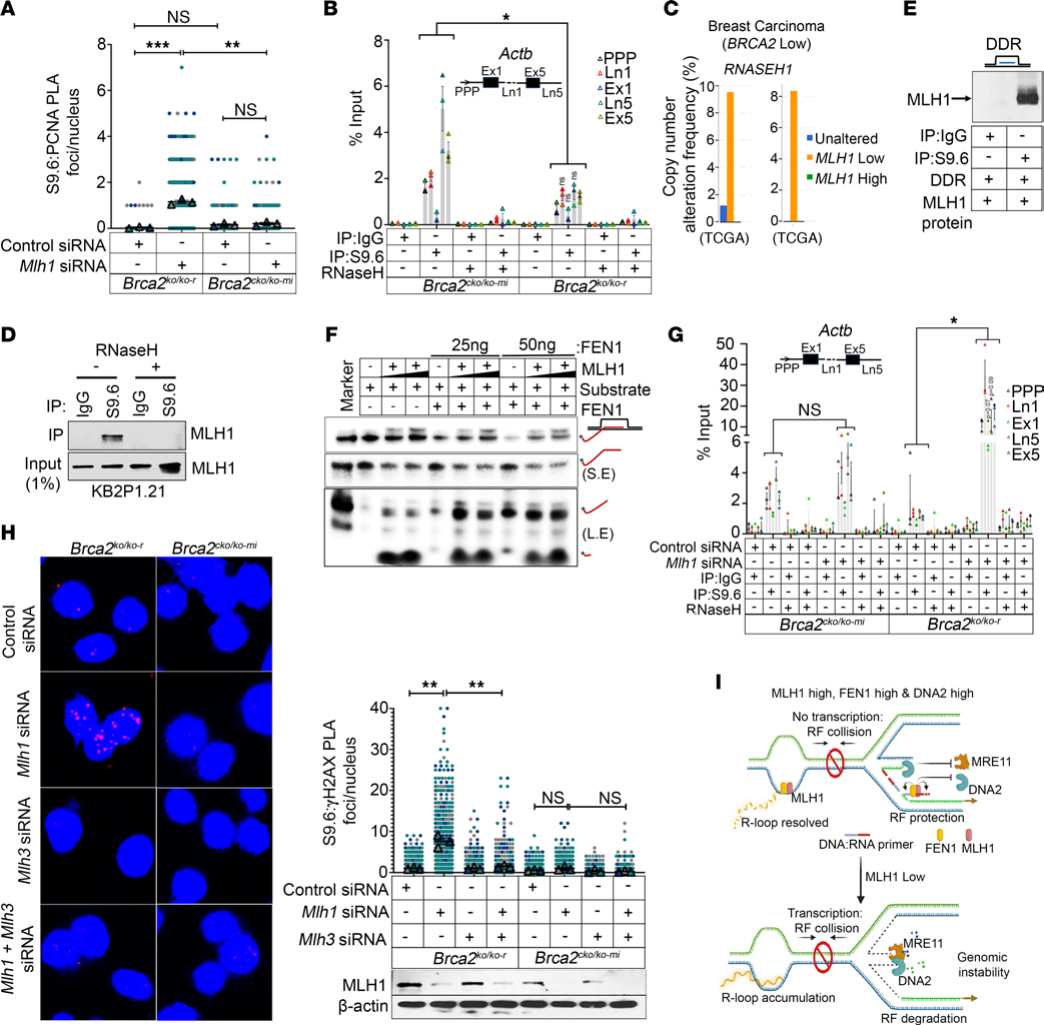
MLH1 resolves R-loops in BRCA2-deficient cells. (**A**) Quantitation of PLA foci formed between S9.6-PCNA in *Mlh1*-silenced *Brca2^KO/KO-r^* or *Brca2^cKO/KO-mi^* clones. (**B**) DRIP assay showing the enrichment of R-loops in *Brca2^KO/KO-r^* or *Brca2^cKO/KO-mi^* clones by RT-qPCR. IgG was used as a negative control, and RNaseH treatment was used to confirm R-loops. Values are expressed as percentages of input. *Actb* genes analyzed are depicted (insert). (**C**) TCGA analysis showing copy number alteration frequency of *RNASEH1* in *BRCA2*-low breast carcinoma samples under *MLH1*-unaltered versus *MLH1*-low (*MLH1* Exp: <-1) versus MLH1-high (*MLH1* Exp: >1) conditions. Data are from ref. 51 (left) and ref. 52(right). (**D**) ChIP-immunoblot showing levels of MLH1 on approximately 500 bp fragmented genomic DNA from KB2P1.21 pulled down with IgG or S9.6. Input of 1% was used as a control. (**E**) ChIP-immunoblot showing levels of MLH1 on in vitro–developed R-loop structure (DDR) pulled down with IgG or S9.6. MLH1 pure protein was used for this assay. (**F**) In vitro nuclease assay showing degradation of RNA strand in P^32^-labeled R-loop substrate by MLH1 (0, 250 ng, and 500 ng) and FEN1 protein (0, 25 ng, or 50 ng). RNA strand with 5′ overhangs is labeled in red. (**G**) DRIP assay showing enrichment of R-loops in *Actb* gene in *Mlh1*-silenced *Brca2^KO/KO-r^* or *Brca2^cKO/KO-mi^* clones by RT-qPCR. (**H**) Representative images of PLA foci formed between S9.6-γH2AX in *Mlh1*- or *Mlh3*-silenced or combination of *Mlh1/3*-silenced *Brca2^KO/KO-r^* or *Brca2^cKO/KO-mi^* clones (left panel). Quantitation of PLA foci (right panel). Lower panel shows immunoblot confirmation of *Mlh1* or *Mlh3* silencing. Original magnification, ×63. (**I**) Model showing replication-transcription conflict in *MLH1*-high/low conditions in *Brca2^KO/KO-r^* clones. Data are represented as mean ± SEM for **A**, **B**, **G**, and **H** (*n* = 3 biological replicate). Data were analyzed using unpaired, 2-tailed Student’s *t* test (**A** and **H**) and unpaired, 2-tailed Student’s *t* test with Holm-Šidák multiple-comparison test (**B** and **G**). *P < 0.05; **P < 0.01; ***P < 0.001.

Replication-transcription conflicts were identified as a major source of RF instability in BRCA2-deficient cells (29). It is also known that abnormal transcription is the major source of persistent/unscheduled R-loop accumulation, causing stalled RF-induced DNA damage, resulting in genomic instability in BRCA2-deficient conditions (3, 30). BRCA2 is known to resolve transcription-associated R-loops and maintain genome stability (3, 4). We confirmed this effect in *Brca2^cKO/KO^* mESCs, where knockdown of *Brca2* resulted in accumulation of R-loops and DNA damage marked by nuclear γH2AX (Supplemental Figure 9, A and B). Since MLH1 deficiency causes impaired RF stability/progression in *Brca2^KO/KO-r^* clones, we investigated the level of collisions between RFs and R-loops by proximity ligation assay (PLA). We silenced *Mlh1* and analyzed the PLA foci formed between S9.6 (which marks R-loops) and PCNA (which marks RFs) to determine the level of fork stalling associated with R-loop accumulation in *Brca2^KO/KO-r^* cells. As expected, we did not observe any significant change in S9.6-PCNA PLA foci between *Brca2^KO/KO-r^* and *Brca2^cKO/KO-mi^* clones under control conditions (Figure 4A), suggesting that fork progression is not impaired in *Brca2^KO/KO-r^* clones. However, depletion of *Mlh1* significantly increased the S9.6-PCNA PLA foci in *Brca2^KO/KO-r^* but not in *Brca2^cKO/KO-mi^* clones (Figure 4A). Interestingly, we also observed an overall increase in transcription in *Brca2^KO/KO-r^* cells that was marked by a significant enrichment of genes involved in regulating/promoting transcriptional activity (*P* < 10^–10^) and the enrichment of RNA polymerase II (RNAPII) in gene portions of actively transcribed, actin (*Actb*) gene loci (Supplemental Figure 9, C–E). However, the level of R-loops at transcriptionally active *Actb* and *Tet1* loci were either low or unaltered in *Brca2^KO/KO-r^* clones in comparison with *Brca2^cKO/KO-mi^* cells by DNA:RNA immunoprecipitation (DRIP) assay, consistent with the above observation that RF progression is stable in *Brca2^KO/KO-r^* clones (Figure 4B and Supplemental Figure 9F).

Though a direct role for MLH1 in regulating R-loop structures has not been reported, MLH1 has been shown to bind multiple DNA structures including Holliday junction and loop-containing DNA structures (19). Increased expression of RNASEH1 has been reported as an indicator for R-loop accumulation (5) because of its well-established role in resolution of R-loops throughout the genome (31). When we analyzed the copy number variations of *RNH1* in patient samples from 2 breast cancer TCGA data sets (TCGA, Cell and Firehose Legacy), we found the copy number amplification was significantly higher in *BRCA2*-low; *MLH1*-low human breast cancer samples compared with unaltered controls (Figure 4C). This correlation was supported by the induction of *Rnh1* expression in *Mlh1*-silenced *Brca2^KO/KO-r^* (~2.5-fold increase), but not in *Brca2^cKO/KO-mi^* clones (Supplemental Figure 9G). We further investigated the role of MLH1 in R-loop resolution by ChIP-immunoblot in KB2P1.21 cells. We detected the presence of MLH1 by immunoprecipitation using R-loop–specific S9.6 antibody, suggesting that MLH1 directly binds to the R-loops (Figure 4D). Next, we used synthetic R-loops and pure MLH1 protein and detected the binding between R-loops and MLH1 by S9.6-mediated ChIP-IB (Figure 4E). Our findings are supported by a recent report that identified MLH1 as an R-loop–binding protein by a global prediction model (32). By in vitro nuclease assay, we observed that MLH1 degraded the RNA strand of R-loop structure with or without tails in a concentration-dependent manner (Figure 4F). FEN1 is also reported to process R-loops (33). While FEN1 processes RNA strand with tails, the level of degradation is less efficient in comparison with MLH1. We next analyzed the level of R-loops in *Mlh1*-silenced *Brca2^KO/KO-r^* and *BRCA2^cKO/KO-mi^* clones using DRIP assay. While we did not observe an increase in R-loop levels in *Brca2^cKO/KO-mi^* clones in response to *Mlh1* knockdown, there was a significant accumulation of R-loops (~10- to 20-fold increase) in *Brca2^KO/KO-r^* clones at all the 3 genomic loci (*Actb*, *Tet1*, *Has2*) (Figure 4G and Supplemental Figure 9, H and I). In addition, we observed high levels of S9.6 and γH2AX nuclear intensity in *Brca2^KO/KO-r^* clones upon *Mlh1* knockdown, whereas no difference was observed in *Brca2^cKO/KO-mi^* clones (Supplemental Figure 10A). Similarly, we found a significant increase in R-loops (~1.5- to 2-fold increase) upon *Mlh1* knockdown in BRCA2-deficient mouse mammary tumor cell line KB2P1.21 (Supplemental Figure 10B). Exposing *Mlh1*-silenced KB2P1.21 cells to a low dose of camptothecin (10 nM) resulted in further augmentation of R-loops (Supplemental Figure 10, C and D). Consistent with this, the levels of γH2AX were also high in these cells (Supplemental Figure 10, C and E). To demonstrate that the DNA damage was contributed to by R-loop accumulation, we performed PLA using S9.6 and γH2AX antibodies. While the numbers of PLA foci were low in control *Brca2^KO/KO-r^* and *Brca2^cKO/KO-mi^* cells, we observed a significant increase in PLA foci in *Mlh1*-silenced *Brca2^KO/KO-r^* cells, but not in *Brca2^cKO/KO-mi^* cells (Figure 4H). We also observed that the enrichment of R-loops and γH2AX observed in *Mlh1*-silenced *Brca2^KO/KO-r^* clones or camptothecin-treated KB2P1.21 cells was completely abolished when *Mlh3* was silenced (Figure 4H and Supplemental Figure 10, C–E). We predict that in the absence of MLH1, MLH3 generates the DNA breaks on the trans DNA strand of R-loop structures, as reported previously in a *Saccharomyces* *cerevisiae* model system (34). These observations suggest that the loss of MLH1 induces R-loop–associated DNA damage in *Brca2^KO/KO-r^* cells.

To determine whether the DNA damage caused by the unresolved R-loops was the source of cell death, we overexpressed *Rnaseh1* in KB2P1.21 cells. While *Mlh1* knockdown resulted in reduction in cell viability, overexpression of *Rnaseh1* rescued the effect of *Mlh1* loss substantially, confirming R-loop–associated DNA damage causes cell death in *Mlh1*-deficient KB2P1.21 cells (Supplemental Figure 10, F and G). Taken together, these observations suggest that MLH1 can process R-loops like FEN1 and reduce R-loop–associated DNA damage, which in turn mitigates replicative stress in *Brca2^KO/KO-r^* clones by relieving conflict between replication and transcription (Figure 4I).

### MLH1 reduces genomic instability in BRCA2-deficient cells.

**Figure 5:**
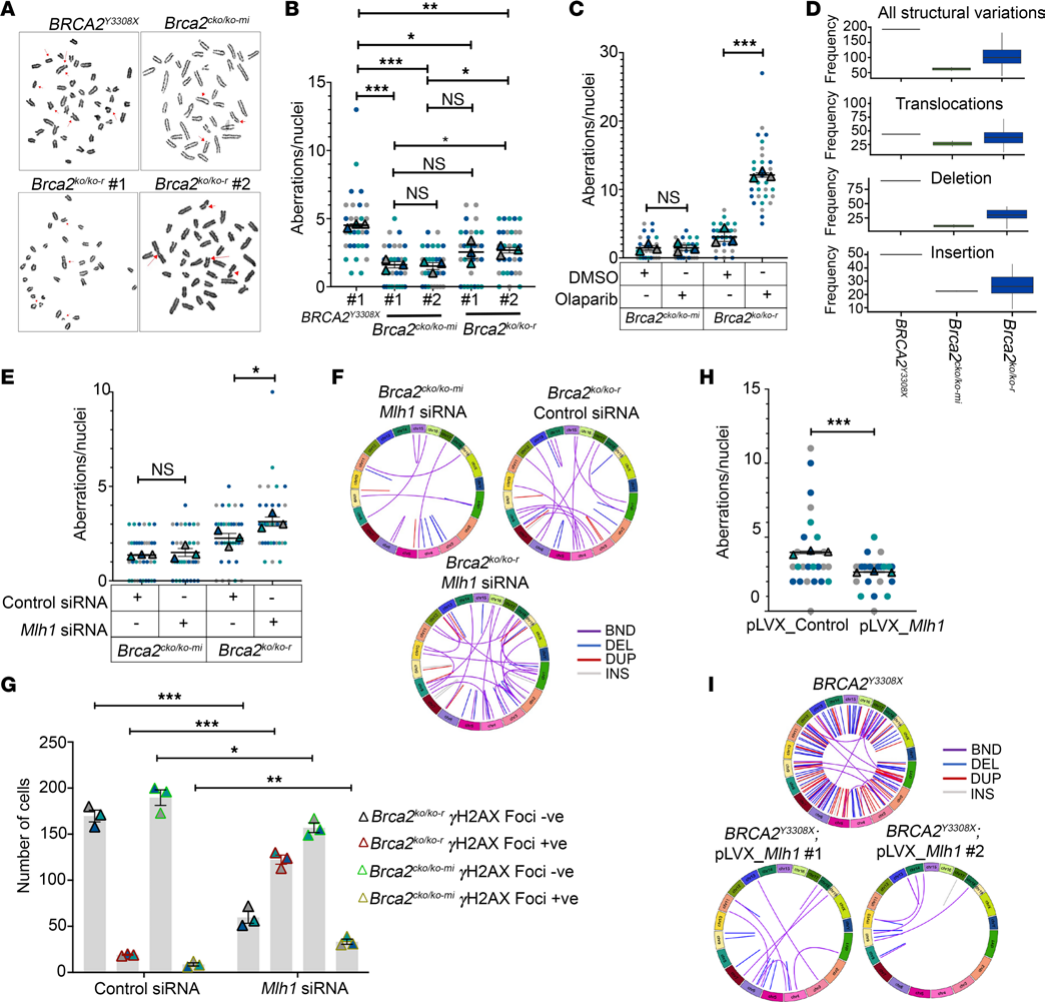
MLH1 suppresses genomic instability in BRCA2-deficient cells. (**A**) Representative images of chromosomal aberrations in *BRCA2^Y3308X^*, *Brca2^KO/KO-r^* (*n* = 2 clones), and *Brca2^cKO/KO-mi^* clones (*n* = 1 clone) as assessed by metaphase spreads. (**B**) Quantitation of chromosomal aberrations in *BRCA2^Y3308X^*, *Brca2^KO/KO-r^* (*n* = 2 clones), and *Brca2^cKO/KO-mi^* clones (*n* = 2 clones) from **A**. Aberrations per nuclei are plotted in a superplot (30 nuclei; *n* = 3 biological replicate; 10 nuclei per replicate). (**C**) Quantitation of chromosomal aberrations in *Brca2^KO/KO-r^* (*n* = 2 clones) and *Brca2^cKO/KO-mi^* clones (*n* = 1 clone) treated with and/or without olaparib (100 nM). Aberrations per nuclei are plotted in a superplot (25–30 nuclei; *n* = 3 biological replicate; 5–10 nuclei per replicate). (**D**) WGS analysis showing the SVs in *BRCA2^Y3308X^*, *Brca2^KO/KO-r^* (*n* = 2 clones), and *Brca2^cKO/KO-mi^* clones (*n* = 19 clones). SVs such as translocations (BND), deletion (DEL), and insertion (INS) are expressed as unique SVs. (**E**) Quantitation of chromosomal aberrations in *Mlh1*-silenced *Brca2^KO/KO-r^* and *Brca2^cKO/KO-mi^* clones. Aberrations per nuclei are plotted in a superplot (30 nuclei; *n* = 3 biological replicate; 10 nuclei per replicate). (**F**) Circos plot depicting levels of SVs in *Brca2^KO/KO-r^* and *Brca2^cKO/KO-mi^* clones upon silencing *Mlh1*. Levels of SVs were normalized to *Brca2^cKO/KO-mi^* (control siRNA) clone. (**G**) Quantitation of γH2AX foci in control or *Mlh1*-silenced *Brca2^KO/KO-r^* and *Brca2^cKO/KO-mi^* clones using immunofluorescence analysis. ImageJ was used to count γH2AX foci manually. (**H**) Quantitation of chromosomal aberrations in control (pLVX_control) or *Mlh1*-overexpressing (pLVX_*Mlh1*) *BRCA2^Y3308X^* clone. Aberrations per nuclei are plotted in a superplot (25 nuclei; *n* = 3 biological replicate; 5–10 nuclei per replicate). (**I**) Circos plot depicting the levels of SVs in *BRCA2^Y3308X^* (normalized to *Brca2^cKO/KO-mi^* clone) and upon *Mlh1* overexpression in *BRCA2^Y3308X^* clones (*n* = 2; *BRCA2^Y3308X^; pLVX_Mlh1* #1 and *BRCA2^Y3308X^; pLVX*_*Mlh1* #2) (normalized to *BRCA2^Y3308X^*; pLVX_control). Data were analyzed using unpaired, 2-tailed Student’s *t* test (**B**, **C**, **E**, and **H**) and unpaired, 2-tailed Student’s *t* test with Holm-Šidák multiple-comparison test (**G**). *P < 0.05; **P < 0.01; ***P < 0.001.

Since *Brca2^KO/KO-r^* mESCs exhibit reduced replicative stress due to MLH1 overexpression, we hypothesized that these cells should exhibit more genomic stability compared with *BRCA2^Y3308X^* mutant mESCs (35). As expected, the basal levels of chromosomal aberrations in *Brca2^KO/KO-r^* clones (~2.5–3.5 aberrations per nuclei) were significantly lower than in the *BRCA2^Y3308X^* cells (~5.5–6 aberrations per nuclei) and were moderately higher than the *Brca2^cKO/KO-mi^* clones (1–2.5 aberrations per nuclei) (Figure 5, A and B). Further, we used the PARP inhibitor olaparib to examine the level of chromosomal aberrations, since it is the known standard drug used to treat BRCA2-deficient tumors. When we treated these cells with olaparib (100 nM), we observed an increase in chromosomal aberrations in *Brca2^KO/KO-r^* (~10–15 aberrations per nuclei), but not in *Brca2^cKO/KO-mi^* clones (Figure 5C). Similar increases in chromosomal aberrations were also observed in *Brca2^KO/KO-r^* upon exposure to camptothecin (10 nM), mitomycin C (MMC) (20 ng/μl), or cisplatin (0.6 μM) (Supplemental Figure 11A). These observations suggest that, while RF protection promotes basal level genomic stability in BRCA2-deficient cells, chemotherapeutic sensitivity is largely determined by HR status, in line with recent reports (36).

To strengthen the above observations, we performed whole-genome sequencing (WGS) to examine the impact of genomic instability on the structural variants (SVs) observed in these clones. The SVs were found to be high in *BRCA2^Y3308X^* and low in *Brca2^cKO/KO-mi^* clones, as expected. *Brca2^KO/KO-r^* clones exhibited an intermediate level of SVs (Figure 5D). These observations further support that the *Brca2^KO/KO-r^* cells have surmounted the genomic instability to levels that support their survival. Next, we investigated the role of MLH1 in suppressing genomic instability in *Brca2^KO/KO-r^* cells. We observed a significant increase in chromosomal aberrations upon silencing *Mlh1* in *Brca2^KO/KO-r^* cells (Figure 5E). Similarly, we observed an increase in SVs upon silencing *Mlh1* in *Brca2^KO/KO-r^* cells by WGS (Figure 5F). Also, the DNA damage marked by γH2AX foci was not significantly different between *Brca2^KO/KO-r^* and *Brca2^cKO/KO-mi^* clones. Notably, upon *Mlh1* knockdown, the levels of γH2AX foci–positive cells significantly increased in *Brca2^KO/KO-r^* cells (Figure 5G and Supplemental Figure 11B). However, we did not observe any difference in chromosomal aberrations upon *Mlh1* silencing with prolonged HU treatment (8 hours) in *Brca2^KO/KO-r^* cells, although silencing of *Mlh1* with prolonged HU treatment (8 hours) in BRCA2-proficient U2OS resulted in increases in chromosomal aberrations (Supplemental Figure 11, C and D). These results further underscore that sensitivity toward chemotherapeutic drugs predominantly relies on HR status.

When we overexpressed MLH1 in *BRCA2^Y3308X^*, there was a significant reduction in chromosomal aberrations and SVs (Figure 5, H and I, and Supplemental Figure 11E). In addition, the overall mutation density in *Brca2^KO/KO-r^* was comparable to that in *Brca2^cKO/KO-mi^* cells (Supplemental Figure 11F). Taken together, these results clearly demonstrate a key role for MLH1 in suppressing genomic instability in unperturbed BRCA2-deficient cells.

### Brca2^L24231P/L24231P^ mice exhibit reduced viability in Mlh1^KO/KO^ background.

To examine the physiological significance of the genetic interaction between *Mlh1* and *Brca2*, we examined how loss of MLH1 affects the survival of *Brca2* mutant mice. Since complete loss of *Brca2* results in early embryonic lethality, we used a mutant line that carries a hypomorphic allele (*Brca2^L2431P^*) (37). This variant corresponds to a pathogenic BRCA2 variant, p.Leu2510Pro, which disrupts the interaction between BRCA2 and DSS1 (37, 38). *Brca2^L2431P/L2431P^* animals are viable, but born at sub-Mendelian ratios (37). *Mlh1^KO/KO^* mice are viable and born at expected Mendelian ratios, but are infertile (39). We crossed homozygous *Brca2^L2431P/L2431P^* and hemizygous *Brca2^L2431P/KO^* mice to *Mlh1^KO/+^* to obtain *Mlh1^KO/+^;Brca2^L2431P/+^* and *Mlh1^KO/+^;Brca2^KO/+^* mice. We intercrossed these double-heterozygous mice to examine the phenotype of *Brca2^L2431P/L2431P^*;*Mlh1^KO/KO^* and *Brca2^L2431P/KO^*;*Mlh1^KO/KO^* mice. While we obtained mice of all possible genotypes, including *Brca2^L2431P/L2431P^*;*Mlh1^KO/+^* and *Brca2^L2431P/+^*;*Mlh1^KO/KO^*, in expected Mendelian ratios, we obtained only 1 viable *Brca2^L2431P/L2431P^*;*Mlh1^KO/KO^* mouse out of 306 live-born offspring (Table 1). Similarly, we obtained mice of all possible genotypes including *Brca2^L2431P/KO^*;*Mlh1^KO/+^*, but failed to obtain any viable *Brca2^L2431P/KO^;Mlh1^KO/KO^* mouse out of 194 live-born offspring (Table 1). Such a significant impact on the survival of *Brca2^L2431P/L2431P^* and *Brca2^L2431P/KO^* mice on a *Mlh1-*deficient background provides strong genetic evidence to support a synthetic lethal genetic interaction between *Mlh1* and *Brca2*.

**Figure 6:**
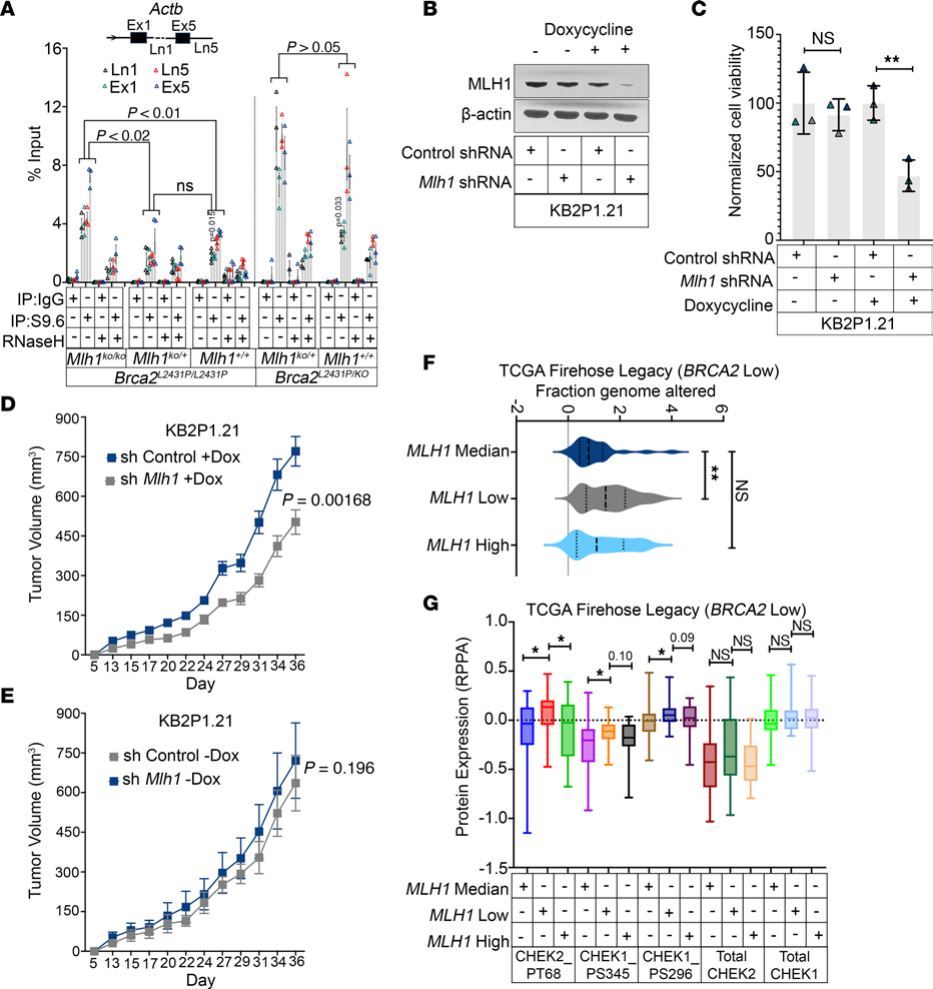
MLH1 loss is lethal during BRCA2 deficiency. (**A**) DRIP assay showing the enrichment of R-loops in *Actb* genes in MEFs from *Brca2^L2431P/L2431P^* (under genotypes of *Mlh1^KO/KO^*;*Mlh1^KO/+^;Mlh1^+/+^*) (*n* = 3 biological replicates for *Mlh1^KO/KO^*; *n* = 2 biological duplicates from 2 independent MEFs for *Mlh1^KO/+^;* *n* = 2 biological replicates from 2 independent MEFs for *Mlh1^+/+^*) and *Brca2^L2431P/KO^* (under genotypes of *Mlh1^KO/+^;Mlh1^+/+^*) (*n* = 3 biological replicates) by RT-qPCR. (**B**) Immunoblot confirmation of doxycycline-induced knockdown of *Mlh1* using *Mlh1* shRNA in KB2P1.21 cells. (**C**) Quantitation of colony formation assay showing the normalized cell viability of doxycycline-induced knockdown of *Mlh1* using *Mlh1* shRNA in KB2P1.21 cells. Control shRNA and no doxycycline treatment was used as a control (*n* = 3 biological replicates). (**D**) Allograft model showing the tumor volume of short hairpin RNA control (shControl) or sh*Mlh1* KB2P1.21 cells under doxycycline induction (*n* = 10 animals). Tumor volume was measured every 2 days using digital caliper. (**E**) Allograft model showing the tumor volume of shControl or sh*Mlh1* KB2P1.21 under no-doxycycline induction (*n* = 8 animals). Tumor volume was measured every 2–3 days using digital calipers. (**F**) TCGA data set analysis (TCGA, Firehose Legacy) showing the percentage of genomes altered in *BRCA2*-low; *MLH1*-median versus *BRCA2*-low; *MLH1*-low breast cancer patient samples. (**G**) TCGA data set analysis (TCGA, Firehose Legacy) showing the phosphorylation status of CHEK1 and CHEK2 in *BRCA2*-low; *MLH1*-median versus *BRCA2*-low; *MLH1*-low versus *BRCA2*-low; *MLH1*-high breast cancer patient samples. The levels of total CHEK1 and CHEK2 act as a control. Note: TCGA breast data set (TCGA, Cell and Firehose Legacy) has some overlapping samples. Data were analyzed using unpaired, 2-tailed Student’s *t* test with Holm-Šidák multiple-comparison test (**A**), unpaired, 2-tailed Student’s *t* test and Wilcoxon’s rank-sum test (**C**), unpaired, 2-tailed Student’s *t* test with Holm multiple-comparison test (**D** and **E**), Wilcoxon’s rank-sum test (**F**), and unpaired, 2-tailed Student’s *t* test (**G**). *P < 0.05; **P < 0.01.

To understand whether R-loop–induced DNA damage contributed to embryonic lethality, we analyzed R-loop levels in MEFs isolated from *Brca2^L2431P/L2431P^* and *Brca2^L2431P/KO^* mice with different MLH1 genotypes. Interestingly, *Brca2^L2431P/KO^* mice showed higher levels of R-loops at basal level and no drastic increase in R-loops was observed in *Brca2^L2431P/KO^*; *Mlh1^KO/+^* MEFs, corroborating the embryonic lethal phenotype of *Brca2^L2431P/KO^* mice. We did not obtain any *Brca2^L2431P/KO^*;*Mlh1^KO/KO^* MEFs due to early lethality of these embryos (Figure 6A). While *Brca2^L2431P/L2431P^* MEFs showed basal level of R-loops on *Mlh1^+/+^* and *Mlh1^KO/+^* genetic backgrounds, significant increases in R-loops were observed upon *Mlh1* loss (*Brca2^L2431P/+^*;*Mlh1^KO/KO^*) (Figure 6A). These results clearly suggest that R-loop accumulation could be a major driver for genomic instability and embryonic lethality observed in *Brca2^L2431P/KO^*;*Mlh1^KO/KO^* mice.

### MLH1 loss suppresses growth of BRCA2-deficient tumors, and its low expression correlates with better prognosis in breast cancer patients.

Next, we determined whether MLH1 loss can suppress growth of BRCA2-deficient mouse mammary tumor cells (KB2P1.21). We generated *Mlh1*-deficient KB2P1.21 cells with doxycycline-inducible *Mlh1* shRNA. While there was no significant reduction in the growth of KB2P1.21 cells without doxycycline treatment, more than 50% growth reduction was observed when *Mlh1* was knocked down using doxycycline treatment (Figure 6, B and C). We examined the impact of *Mlh1* knockdown on the growth of KB2P1.21-derived allograft tumors in mice. We subcutaneously injected KB2P1.21 cells containing doxycycline-inducible *Mlh1* shRNA. We observed a significant reduction in tumor growth when *Mlh1* was knocked down by *Mlh1* shRNA (Figure 6, D and E). These results signify the importance of MLH1 in supporting the survival of BRCA2-deficient tumors.

We also analyzed the TCGA database to determine the clinical relevance of the above observation. The fraction genome altered was high in *BRCA2*-low; *MLH1*-low samples compared with *BRCA2*-low; *MLH1*-median (refers to unaltered expression) and *MLH1*-high samples (Figure 6F and Supplemental Figure 12A). This was supported by the enrichment of CHEK1/2 phosphorylation at Thr68, Ser296, and Ser345 residues in *BRCA2*-low; *MLH1*-low samples (Figure 6G and Supplemental Figure 12B). No difference in the genome alterations or change in CHEK1/2 phosphorylation status was observed in *BRCA2*-high; *MLH1*-median, -low, or -high conditions (Supplemental Figure 12, C–F). In addition, *MLH3*-median, -low, and -high samples had no effect on genome alterations or CHEK1/2 phosphorylation in *BRCA2*-low or -high samples (Supplemental Figure 12, G–I).

To determine whether enrichment of MLH1 mutation is reduced in *BRCA2* mutant samples in comparison with non-MLH1 MMR genes, we pooled multiple TCGA data sets and examined *MLH1* mutation versus *PMS2/MSH6/MSH2* in MSI-high (microsatellite instability) cancer samples (*n* = 10,068). In the MSI-high group, we obtained 42 *MLH1* mutations and 117 *PMS2/MSH6/MSH2* cases. Interestingly, only 11.9% (5 out of 42 samples) of samples with *MLH1* mutation showed *BRCA2* mutation, whereas 25.64% (30 out of 117 samples) of samples with *PMS2/MSH6/MSH2* mutation showed *BRCA2* mutation (Table 2). These results suggest the enrichment of *BRCA2* mutation as a second hit in non-*MLH1* MMRD cancers, but not in *MLH1-*mutant MMRD cancers, corroborating the synthetic lethality of *Brca2* mutant mice on a *Mlh1^KO/KO^* background. These findings suggest that MLH1 deficiency induces DNA damage and genomic instability in BRCA2-deficient breast cancer patient samples, resulting in better prognosis (Figure 1H).

### Estrogen induces MLH1 expression directly via ERα in breast cancer cells and PDX breast cancer models.

**Figure 7:**
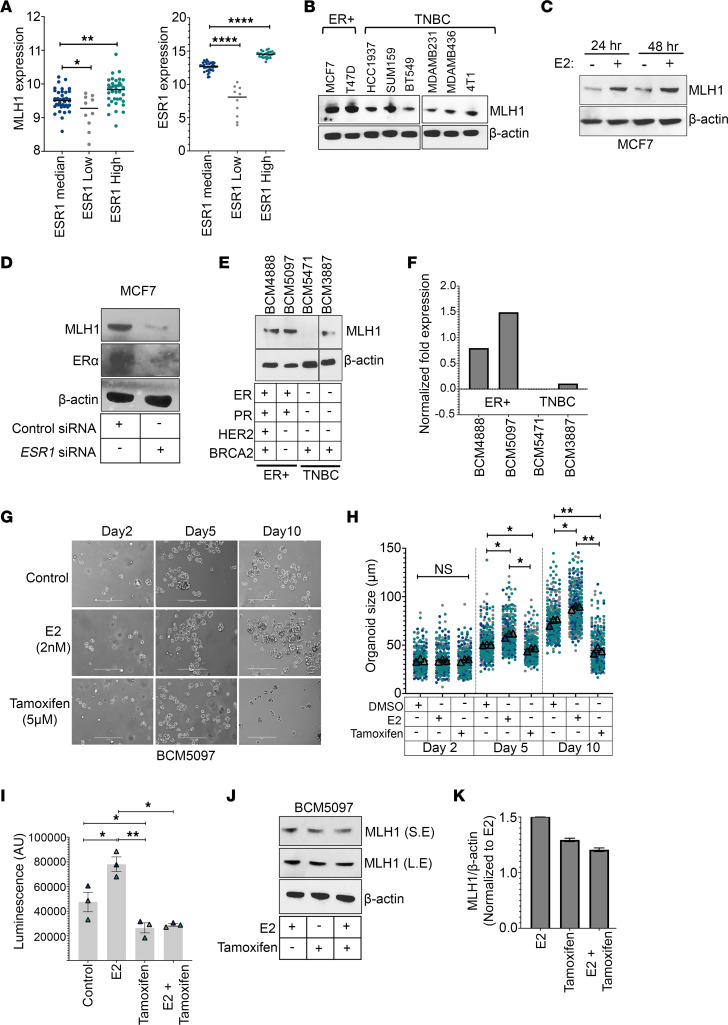
ERα induces MLH1 expression in ER^+^ breast cancer cells. (**A**) TCGA data set analysis (TCGA, Firehose Legacy) showing the expression of ESR1 (left panel) and MLH1 (right panel) in ESR1-median; ESR1-low and ESR1-high breast cancer patient samples. (**B**) Immunoblot showing the expression of MLH1 in ER^+^ and TNBC cell lines. (**C**) Representative immunoblot showing expression of MLH1 in MCF7 cells upon E2 supplementation (2 nM) for 24 to 48 hours. (**D**) Immunoblot showing expression of MLH1 and ERα in control or ERα-silenced MCF7 cells. (**E**) Immunoblot and (**F**) quantification showing expression of MLH1 in PDX breast cancer models of the indicated subtypes with WT (+) or mutant (–) BRCA2 status. (**G**) Representative images (left panel) of BCM5097 organoids from day 2, day 5, and day 10 of culture in the presence of E2, tamoxifen, or DMSO (control) as indicated. Scale bars: 400 μm. (**H**) Quantitation of **G** showing diameter of organoids (*n* > 100 from 3 biological replicates). (**I**) Luminescence-based assay quantifying live cells in BCM-5471 organoids cultured in the presence of E2 (2 nM) for 5 days and/or tamoxifen (5 μM) for 3 days. DMSO was used as control (*n* = 3 biological replicates). (**J**) Immunoblot showing the expression of MLH1 in BCM5097 tumor organoids as in panel **I** and **K** quantitation. Data were analyzed using unpaired, 2-tailed Student’s *t* test (**A**) and paired, 2-tailed Student’s *t* test (**H** and **I**). *P < 0.05; **P < 0.01; ****P < 0.0001.

Given the contrasting correlation of MLH1 expression with colon versus breast cancer survival probability (Figure 1, H–J) and the observation that most *BRCA2* mutation carriers develop ERα-positive (ER^+^) breast cancer, we investigated MLH1 expression in breast tissues. Analysis of the TCGA breast cancer database showed that *MLH1* expression was higher in tumor tissue in comparison with that of normal controls (Supplemental Figure 13A). Among tumor tissues, MLH1 expression was higher in luminal compared with basal subtypes both at RNA (Supplemental Figure 13B) and protein levels (Supplemental Figure 13C), correlated positively with ERα expression, and tended to cooccur in breast cancer (Figure 7A and Supplemental Figure 13, D–H). Conversely, MLH1 was low in 66% of ERα-negative and 74% of triple-negative breast cancer (TNBC) patient samples, which lack ERα expression (Supplemental Figure 13G). Therefore, we investigated the influence of ERα on MLH1 expression. ERα regulates gene expression through binding to estrogen response element–binding (ERE-binding) elements (40). Interestingly, the promoter of MLH1 harbors ERE-binding regions (41). By in silico JASPAR (https://jaspar.elixir.no) prediction, we confirmed the presence of ERE elements in the promoter of MLH1 (Supplemental Figure 13, I and J). Survey of a panel of breast cancer cell lines showed higher expression of MLH1 in ERα-positive cell lines when compared with TNBCs (Figure 7B). We selected ER^+^ MCF7 cells for further analysis and found that supplementation with 17β-estradiol (E2) induced the expression of MLH1 (Figure 7C) and silencing of the ERα gene (*ESR1*) reduced the expression of MLH1 (Figure 7D). To extend the ERα and MLH1 relationship in vivo, we next surveyed the PDX models database (Supplemental Figure 14A) and selected BCM-5097 based on BRCA2 mutant status. Interestingly, BCM-5097 is an ER^+^; progesterone receptor–positive (PR^+^) PDX and the mRNA expression of *MLH1* was relatively higher than in most other PDX models (Supplemental Figure 14A). We confirmed high expression of MLH1 in BCM-5097 at the level of protein (Figure 7, E and F). We generated organoids to analyze the effect of E2 and the E2 antagonist tamoxifen on organoid growth and MLH1 expression. As expected, E2 induced, while tamoxifen inhibited, the growth of organoids (Figure 7, G–I). Furthermore, the expression of MLH1 was also reduced upon tamoxifen treatment (Figure 7, J and K). These observations demonstrate that MLH1 is a bona fide target of ERα and the ERα/MLH1 axis may contribute to BRCA2-deficient tumor initiation and progression in breast tissue.

## Discussion

MLH1 is a key DNA MMR protein (42). Mutation in several MMR genes, including *MLH1*, results in increased mutational load and microsatellite instability that is a major driver of colon cancer (43) or Lynch syndrome (44). Though multiple studies have attempted to link MLH1 loss with predisposition to breast cancer, the findings are inconclusive (45–48). Our present study describes a role of *MLH1* as a genetic interactor of *BRCA2* and its relevance to the survival of BRCA2-deficient cells and *Brca2* mutant mice. This genetic interaction was revealed by the functional characterization of *Brca2*-null mESCs that were rescued by transient inhibition of MRE11. We found MLH1 overexpression contributes to *Brca2^KO/KO-r^* mESC viability.

We describe an important function for MLH1 in protecting stalled RFs from DNA2 nuclease. MRE11 is reported to be the predominant nuclease that targets stalled forks for degradation in the absence of BRCA2 (2). Strikingly, we did not observe MRE11 nuclease activity at stalled RFs in *Brca2^KO/KO-r^* mESCs with MLH1 overexpression. Knockdown of *Mlh1* rendered stalled forks susceptible to degradation by DNA2 nuclease. While the precise cause of preferential access of DNA2 is not clear, it is possible that excess availability of a specific nuclease enhances its accessibility to the stalled forks. This is supported by the fact that overexpression of DNA2 blocks the MRE11-mediated fork degradation in *Brca2-*deficient mouse mammary tumor cells. It is unclear whether increased expression of *Dna2* steered the selection of *Brca2^KO/KO-r^* mESCs with higher *Mlh1* expression to protect them from DNA2-mediated excessive degradation of stalled RFs and support cell survival. In addition to a direct role in maintaining RF integrity and mitigating replicative stress, we found MLH1 to promote stable fork progression by suppressing R-loop accumulation.

In HR-proficient *Brca2^cKO/KO^* cells, the role of MLH1 in fork protection is not essential for cell viability. This is consistent with a recent study focused on the functional analysis of a *Brca2* mouse model, wherein HR was reported to play a predominant role and fork protection had only minimal effect when cells were HR proficient (36). However, in HR-deficient cells, fork protection can contribute to cell viability by suppressing genomic instability (15, 16, 49). In the present study, we have shown that MLH1 resolves R-loops, which suppresses R-loop–associated fork stalling/collapse, resulting in genomic stability in *Brca2^KO/KO-r^* cells. Depletion of MLH1 led to R-loop–associated fork stalling/collapse in *Brca2^KO/KO-r^* cells. In contrast, in *Brca2*^cKO/KO^ cells, R-loops were minimal even upon MLH1 depletion, and hence, we did not observe any replication-transcription conflicts, suggesting that these cells may not experience severe stalled RF-induced DNA damage and genomic instability. Even in cases in which RFs stall independently of R-loops (such as G4 structures, etc.), the resulting DNA damage could be repaired by active HR, and hence MLH1 depletion has no effect on cell viability in *Brca2*^cKO/KO^ cells. While R-loop resolution appears to be the critical function for viability, RF protection by MLH1 is also critical for viability of HR-deficient cells under circumstances in which forks stall independently of R-loops. Consistent with this, *Mlh1* deficiency augmented the chromosomal aberrations and DNA damage in *Brca2^KO/KO-r^*, but not in *Brca2^cKO/KO-mi^* cells.

**Figure 8:**
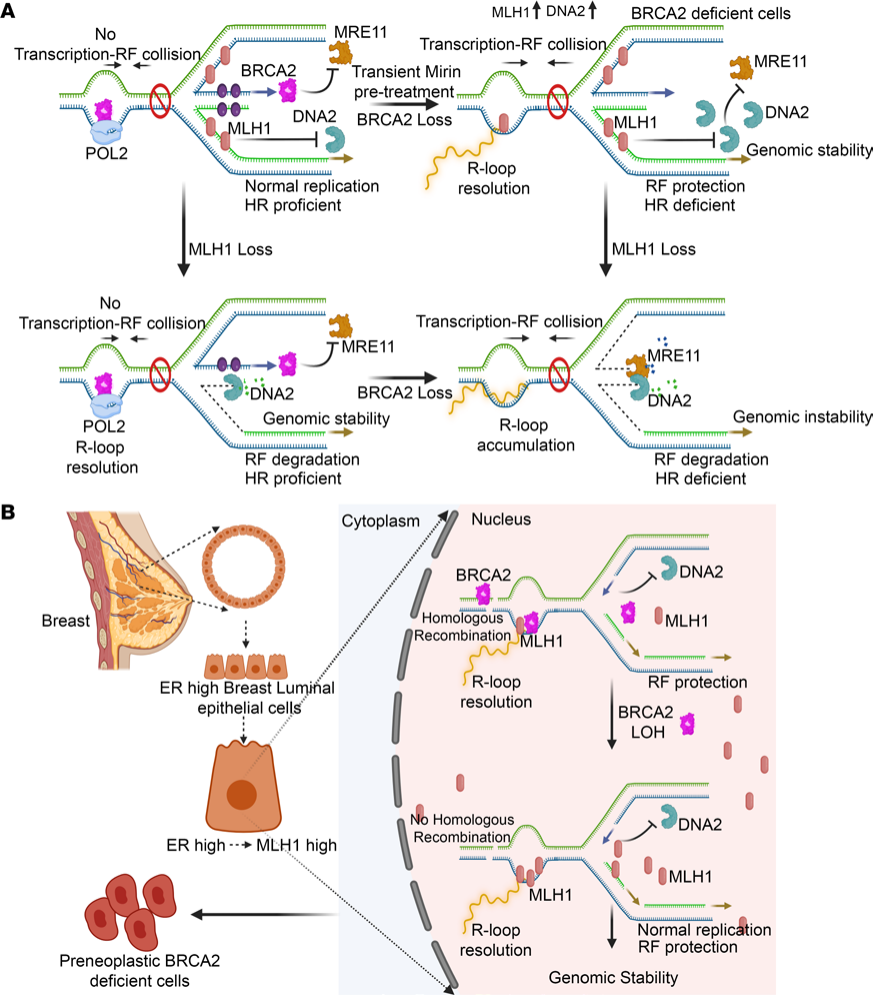
Model depicting the role of MLH1 in BRCA2-deficient cell viability by relieving replicative stress. (**A**) Schematic representation of the model depicting the role of MLH1 in relieving the replicative stress in BRCA2-deficient cells. MLH1 protects RFs from DNA2 and resolves R-loops in BRCA2-deficient conditions, contributing to genomic stability. Hence, loss of MLH1 is lethal for BRCA2-deficient cells. (**B**) Schematic model depicting tissue-specific induction of MLH1 by estrogen supporting the survival of BRCA2 heterozygous cell undergoing LOH, resulting in the development of preneoplastic BRCA2-deficient breast cancer cells.

We have demonstrated a synthetic lethal interaction between BRCA2 and MLH1 by a severe reduction in the survival of *Brca2* mutant mice on a *Mlh1^KO/KO^* genetic background. Since MLH1 loss is known to cause increased genomic instability due to MMR defect, we cannot rule out the possibility that the lethality of *Brca2^Leu2431Pro/Leu2431Pro^;Mlh1^KO/KO^* mice may be caused by the additive effect of the loss of HR and MMR pathways. But given the specific effect of loss of *Mlh1* on the viability of *Brca2^KO/KO-r^* mESCs and not of any other MMR genes, the increased replicative stress and R-loop accumulation is likely the cause of lethality and not the additional mutational load. Thus, we have demonstrated that MLH1 can relieve replicative stress and promote genomic stability and shown how its loss can contribute to synthetic lethality in BRCA2-deficient cells (Figure 8A).

The interaction between BRCA2 and MLH1 is also supported by the TCGA data set from human breast cancer patients. Reduced expression of *MLH1* in *BRCA2-*low tumor-induced DNA damage resulted in better breast cancer prognosis. Similar correlation was previously reported where restoration of partial HR or fork protection in BRCA2 mutant tumors led to poor patient survival (15–17). It is interesting to note that *MLH1* deficiency predisposes to colorectal cancers because of high mutational load, resulting in poor prognosis, whereas in breast cancer, *MLH1* deficiency results in better prognosis. Given its contrasting role in breast/colon cancers and no significant clinical effect in other cancer types, it is possible that MLH1 functions in a tissue-specific manner. This is corroborated by a positive correlation between ERα and MLH1 expression in human breast cancer samples in TCGA. BRCA2 LOH can be tolerated in MLH1-expressing cells, and therefore ER^+^ cells would have the ability to give rise to BRCA2 mutant tumors that are selected for maintenance of ER expression (Figure 8B). This is consistent with the fact that most BRCA2-defective tumors are ER^+^; PR^+^ and HER2 negative (50). More rigorous studies are warranted to establish the role of ERα-induced MLH1 in initiation and progression of BRCA2-deficient breast tumors.

## Methods

### Sex as a biological variable.

Our study examined male and female animals, and similar findings are reported for both sexes.

### Generation of Brca2^KO/KO-r^ mESC clones.

*Brca2^cKO/KO^* cells were generated from an AB2.2 mESC line, as described previously (35). To generate *Brca2^KO/KO-r^* mESC clones by mirin pretreatment, *Brca2^cKO/KO^* cells were treated with 10 μM mirin for 3 hours, as described previously (15). Cells were harvested and 10 million cells were electroporated with 25 μg of *PGK-Cre* to delete the conditional allele of *Brca2* in *Brca2^cKO/KO^* cells. Recombinant clones were selected in HAT media. PL2F7 cells are sensitive to HAT due to a mutation in the endogenous *Hprt* gene. In these cells, the *loxP* sites used to generate the conditional allele are flanked by 5′ and 3′ halves of human *HPRT* minigene. Upon recombination, *Brca2* is deleted and a functional *HPRT* minigene is generated, which renders the cells HAT resistant. This allows selection of the recombinant clones. HAT-resistant clones were genotyped by Southern blotting, as described earlier (15). A DNA probe specific to distinguishing conditional (*cKO*, 4.8 kb) and knockout allele (*KO*, 2.2 kb) of *Brca2* was used as described previously (15), and imaging was performed using an Amersham Typhoon image scanner (Cytiva).

### DNA fiber assay.

To determine RF speed, subconfluent, asynchronous cells were sequentially pulsed with thymidine analogues, 8 μg/ml of CldU and 90 μg/ml of IdU for 30 minutes each. To analyze fork restart, cells pulsed with 8 μg/ml of CldU for 30 minutes were washed with PBS and released in media containing 4 mM HU for 3 hours to stall the RF and then labeled with IdU for another 30 minutes. For fork protection assay, cells were pulsed sequentially with 8 μg/ml of CldU and 90 μg/ml of IdU for 30 minutes each before stalling the RF with 4 mM HU for 3 hours. Cells were treated with all siRNAs for 48 hours before treating them with CldU. AFter drug treatment, the cells were lysed with fresh cell lysis buffer (200 mM Tris-HCl, pH 7.4, 50 mM EDTA, 0.5% SDS) on glass slides for 8 minutes and DNA was spread by tilting the glass slide at about a 60° angle. DNA fibers were then fixed with methanol/acetic acid (3:1) overnight at 4°C. Fibers were then rehydrated in PBS, denatured in 2.5 M HCl for 1 hour at room temperature (RT), and blocked with blocking solution (2% BSA, 0.1% Tween 20, 1× PBS) for 40 minutes. The slides were then incubated with anti-BrdU antibody (mouse, 347580, BD; rat, ab6326, Abcam) overnight at 4°C, before being stained with secondary antibody conjugates (Alexa Fluor 488–conjugated anti-mouse IgG secondary antibody and Alexa Fluor 594–conjugated anti-rat IgG secondary antibody) for 1 hour at RT. The slides were then mounted with ProLong gold antifade reagent (Thermo Fisher Scientific, P36934) and imaged using a Zeiss Axio Imager Z1 microscope; fiber lengths were measured using ImageJ software (NIH).

### Statistics.

Statistical analysis was performed using either GraphPad Prism or custom-written scripts in the R language for statistical computing. Pairwise comparisons of statistical samples were performed using either the unpaired or paired, 2-sided *t* test or Wilcoxon’s rank-sum test in cases of nonnormality. The *P* values from pairwise sample comparisons were adjusted for multiple comparisons using the Holm method or the Holm-Šidák method, which can have increased statistical power in certain cases. *P* value adjustment using R-studio was performed separately on separate blocks of samples, as described in Supplemental Methods. The χ^2^ *P* values for genetic crosses were obtained using Microsoft Excel, version 16.82.

### Study approval.

The study protocol (#21-471) was approved by the Animal Care and Usage Committee (ACUC) of the National Cancer Institute, Frederick. Experiments on all animals were done following the recommendations of the *Guide for the Care and Use of Laboratory Animals* (National Academies Press, 2011). Animals were housed and maintained under a 12-hour light/12-hour dark cycle at temperatures in the range of 20°C to 27°C and 30% to 70% relative humidity.

### Data availability.

RNA-Seq data were submitted to the NCBI’s Gene Expression Omnibus database (GEO GSE220973). WGS data were submitted to the NCBI’s Sequence Read Archive database (SRA BioProject PRJNA1030135). Values for all data points in graphs are reported in the Supporting Data Values file. For Supplemental Figure 12, A–D, and Supplemental Figures 13 and 14, graphs were directly downloaded from the respective databases, and we do not have access to the raw data points. However, based on the criteria described, these graphs can be generated using these databases.

## Author contributions

SK Sengodan performed most of the experiments and acquired and analyzed the data. XH and VKS analyzed the WGS data. VP and SSK helped with the DNA fiber assay. MEA helped with animal studies. AYM and TM helped with statistical analysis. RS provided breast cancer cell line protein lysates and helped with bioinformatics analysis. ES, KB, and LM provided PDX tumor samples. SSB performed cytogenetic experiments. YZ and BT performed RNA-Seq and WGS. SK Sharan conceived and supervised the study. SD supervised the WGS analysis. SK Sengodan and SK Sharan wrote the manuscript. All the authors reviewed and commented on the manuscript.

## Supplementary Material

Supplemental data

Unedited blot and gel images

Supporting data values

## Figures and Tables

**Table 1:**
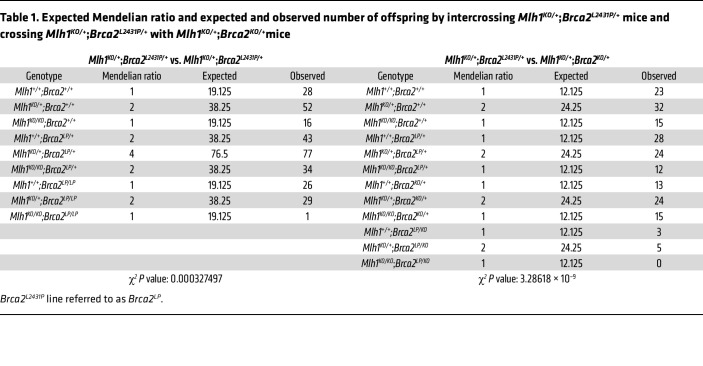
Expected Mendelian ratio and expected and observed number of offspring by intercrossing *Mlh1^KO/+^*;*Brca2^L2431P/+^* mice and crossing *Mlh1^KO/+^*;*Brca2^L2431P/+^* with *Mlh1^KO/+^*;*Brca2^KO/+^*mice

**Table 2:**
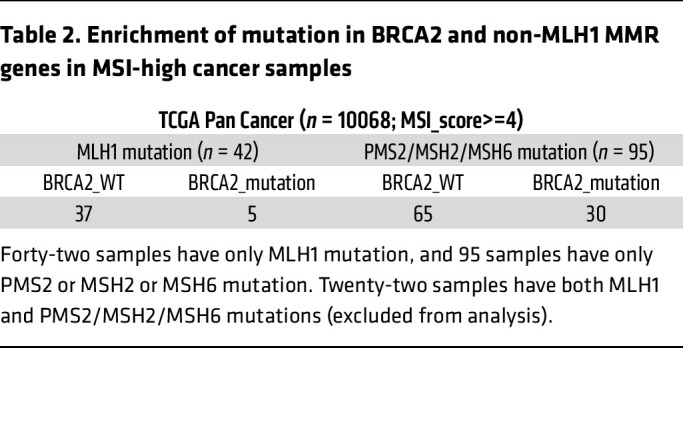
Enrichment of mutation in BRCA2 and non-MLH1 MMR genes in MSI-high cancer samples

## Version 1 01/25/2024

In-Press Preview

## Version 2 04/01/2024

Electronic publication
