# Supplementary material for: Mismatch repair protein MLH1 suppresses replicative stress in BRCA2-deficient breast tumors
Source: J Clin Invest. 2024 Jan 25;134(7):e173718. doi: 10.1172/JCI173718 (PMC10977984; doi:10.1172/JCI173718)
Supplement: Supplemental data [file jci-134-173718-s069.pdf]

Supplemental Information

Supplemental Methods

Cell culture and Transfection

PL2F7 mouse embryonic stem cells (*Brca2*$^{cko/ko}$ mESCs) derived from AB2.2 mouse embryonic stem cell line by flanking one allele of *Brca2* with two LoxP sites and knocking out other allele of *Brca2* were cultured in knockout DMEM (M15 media) supplemented with 15% FBS on mitotically inactive SNL feeder monolayer at 37^0C with 5% CO_2 as previously described (1). Mouse mammary tumor cell lines (KB2P1.21R2 and KB2P1.21) (a kind gift from Dr. Jos Jonkers) and human osteosarcoma cell line, U2OS (obtained from American Type Culture Collection (ATCC), Rockville, MD) were cultured in DMEM/F-12 (Life Technologies) supplemented with 10% FBS as described earlier (2). Human kidney cell line, HEK293T (obtained from American Type Culture Collection (ATCC), Rockville, MD) and human breast cancer cell line, MCF7 (obtained from American Type Culture Collection (ATCC), Rockville, MD) were cultured in DMEM (Life Technologies) supplemented with 10% FBS. Cell lines are mycoplasma free and STR profiled. For RNA interference, cells were transfected with indicated siRNA's using Lipofectamine™ RNAimax Transfection Reagent (ThermoFisher Scientific-13778075) for 48h as per manufacturer's protocol. For plasmid transfection, cells were either electroporated (PGK-Cre) or transfected with Lipofectamine 2000 for 48h as per manufacturer's protocol. For rescue experiments, 1×10^7 mESCs were electroporated with 20µg of PGK-Cre plasmid using gene pulser (Bio-Rad) and the cells were allowed to grow for 36h on feeder plate before being selected with HAT followed by colony picking as per the protocol described earlier (1).

Generation of stable cell lines

For generation of stable cell line for Dox inducible *Mlh1* shRNA, HEK293T cells were transfected with MLH1 SMARTvector Inducible Lentiviral shRNA vector (Dharmacon) using Trans lentiviral shRNA packaging system (Dharmacon) by calcium phosphate transfection reagent as per manufacturer's protocol. KB2P1.21 cells were then transduced with lentiviral particles from MLH1 SMARTvector Inducible Lentiviral shRNA vector along with polybrene (8ug/ml) and stable cells were generated by selecting the cells using puromycin (3ug/ml) for 5days. Dox induction (*in-vitro*) (10ug/ml) was given for 72hr before knockdown of MLH1 was analyzed by Immunoblot. For generation of *Rnaseh1* over expressing stable cell line, HEK293T cells were co-transfected with pLVX_control vector or pLVX_*Rnaseh1* vector along with packaging vectors. The media containing the lentiviral particles were collected after 48hr before being transduced on KB2P1.21 cells. The cells were selected with 3ug/ml of puromycin for 5days and stable clones were confirmed by RT-PCR. For MLH1 over expression in PL2F7-A10(puromycin marker deleted PL2F7) mESCs, MSCV_*Mlh1* lentiviral particles were generated by co-transfecting HEK293T cells with MSCV_*Mlh1* plasmid and packaging plasmids. PL2F7-A10 were transduced with empty MSCV (Clonetech; puromycin resistance) or MSCV_*Mlh1* lentiviral particles along with polybrene (8ug/ml) for 48hr without SNL feeder on gelatin coated 10cm plate. These cells were trypsinized and 10^4 cells were seeded on 10cm plate containing SNL feeder. Post 24 hr of seeding, these clones were selected with 3ug/ml of puromycin for 5days and the stable clones were confirmed by Immunoblot. For generation of *Mlh1* over expressing stable cell line in PL2F7-A10 mESC expressing a truncated allele of BRCA2 (Y3308X), cells were transduced with pLVX_control vector or pLVX_*Mlh1* lentiviral particles for 48 hr with polybrene (8ug/ml). The clones were selected with 3ug/ml of puromycin, and pool of resistant clones were used for whole genome sequencing and cytogenetics analysis.

ChIP-PCR and DRIP-PCR

For ChIP, cells crosslinked with 1% formaldehyde were lysed in cell lysis buffer (20mM Tris-HCl pH 8.0, 85mM KCl, 0.5% NP40) with protease inhibitor for 15min in ice. Following a centrifugation at 800g for 10min at 4^0C, the nuclear pellet was lysed with nuclear lysis buffer (10mM Tris-HCl pH 8.0, 1% NP40, 0.5% sodium deoxycholate, 0.1% SDS) for 30min on ice. The samples were sonicated at diagenode biorupter300 (30s ON and 30s OFF, 16cycles) to yield 300-600bp chromatin fragments.10ug of chromatin fragments were either treated with or without RNase H (1.5U per ug of DNA) for 4h at 37^0C. 10ul of samples were removed and used as input. Chromatin fragments were diluted with ChIP dilution buffer (16mM Tris-HCl pH 8.0, 1.2mM EDTA, 1.1% Triton X-100, 167mm NaCl, 0.01% SDS) and immunoprecipitated with 2-4ug of indicated antibodies and 20ul of pre-blocked Dynabeads overnight at 4^0C with rotation. Next day, beads were washed successively with Low salt buffer (20mM Tris-HCl pH 8.0, 1% Triton X-100, 2mM EDTA, 0.1% SDS, 150mM NaCl), high salt buffer (20mM Tris-HCl pH 8.0, 1% Triton X-100, 2mM EDTA, 0.1% SDS, 500mM NaCl), LiCl buffer (10mM Tris-HCl pH 8.0, 1mM EDTA, 1% sodium deoxycholate, 1% NP40, 0.25M LiCl) and TE buffer (20mM Tris-HCl pH 8.0, 1mM EDTA) for 5min at room temperature in rotation. Beads were separated by magnetic stand and antibody bound DNA was eluted by incubating with elution buffer (100mM $NaHCO_3$, 1% SDS) containing proteinase K for 2h at 65^0C followed by 30min at 95^0C. The DNA fragments were purified by PCR purification kit (QIAquick PCR Purification Kit; Qiagen-28104) and analyzed by RT-qPCR with indicated primers as mentioned elsewhere. For DRIP, the genomic DNA was isolated from uncross-linked cells by incubating with cell lysis buffer (20mM Tris-HCl pH 8.0, 85mM KCl, 0.5% NP40) and Proteinase K overnight at 65^0C and the DNA was purified by phenol/chloroform purification method. 10ug of purified DNA was treated with a cocktail of

restriction enzymes (20U *Xba*I, 20U *Eco*RI, 20U *Hind*III, 25U SSPI and 10U NotI) with and/or without RNase H in buffer 2.1 overnight at 37^{0}C to get 300-500bp chromatin fragments. The chromatin fragments were purified by QIAquick PCR purification kit, diluted with 1X DRIP buffer (10mM NaH_2PO_4, 0.14M NaCl and 0.05% Triton-X) and immunoprecipitated with 2-4ug of S9.6 antibody and 20ul of pre-blocked Dynabeads overnight at 4^{0}C with rotation. Next day, the beads were washed twice with 1X DRIP buffer and once with 1X DRIP buffer+330mM NaCl for 5min at room temperature in rotation.

Immunofluorescence

Immunofluorescence was carried out as described earlier (3). All siRNA transfections (48hr post transfection) for immunofluorescence were performed on cells seeded on coverslips and the cells were fixed using methanol: acetone (1:1). For RAD51 staining, PL2F7 mESCs seeded on coverslips were irradiated at 5 Gy and 3hr post recovery, cells were fixed and stained with anti-γH2AX (1:200, Millipore JBW301) and anti-RAD51 (1:200, Millipore PC130). Imaging was done in Zeiss LSM 710 confocal microscope and nuclear intensity were mapped and calculated using ImageJ. DAPI was used as nuclear stain.

iPOND

iPOND was performed as described earlier (1). Briefly, cells were fed with 10μM EdU for 10 min to label nascent DNA and DNA-Protein complexes were cross-linked using 1% formaldehyde at RT for 10 min. Cells were harvested, permeabilized with 0.25% Triton X-100 and click chemistry was carried out at dark using biotin azide (20 μM) in click reaction buffer (2mM $CuSO_4$ and 10mM sodium-L-ascorbate) for 2 hr at RT to biotin conjugate the EdU labelled nascent DNA. Click chemistry using DMSO was used as a negative control during click reaction. Cells were resuspended in lysis buffer and chromatin fragments were made by sonication using Bioruptor at

4^0C for 15 cycles (30sec on/off per cycle). Supernatant were incubated with streptavidin-MyOne C1 beads (Life Technologies) for 1hr and captured proteins were eluted by boiling the beads in 2x lamelli buffer containing 2-mercaptoethanol for 45 min at 95^0C. Proteins were resolved on 10% SDS PAGE and detected using western blot. 2% input sample was used as a control.

Flow cytometry and Immunofluorescence-based analysis of replication speed

For flow cytometry, asynchronous, subconfluent cells were labeled with 10μM EdU for 1h, harvested, fixed with formaldehyde for 15min, washed once with 3ml of 1% BSA in PBS before saponin based permeabilization and click-iT reaction was performed to detect EdU using Click-iT EdU Flow Cytometry Cell Proliferation Assay kit (ThermoFisher Scientific-C10419) as per manufacturer's protocol. Antibody staining for γH2AX (Biolegend-613404) was done at RT for 1h and total DNA was stained using FxCycle™ Violet (ThermoFisher Scientific-F10347). The cells were analyzed on Flow cytometer (BD LSRII) and the results were analyzed using FlowJo software. For Immunofluorescence, cells on coverslips were labeled with 10μM EdU for 1h, fixed and permeabilized using methanol: acetone (1:1) and click-iT reaction was performed using Click-iT™ EdU Cell Proliferation Kit for Imaging (ThermoFisher Scientific-C10340) as per manufacturer's protocol. Nuclear staining was done using DAPI. Imaging and analysis were performed using Zeiss LSM 710 and ImageJ as described previously.

Colony formation assay (CFA)

For CFA, a total of 2000 $Brca2^{ko/ko\text{-}r}$ or $Brca2^{cko/ko\text{-}mi}$ mES cells were seeded in 6-well plate and allowed to grow on feeder plate for 14 days. The colonies were then fixed and stained with 0.5% (w/v) crystal violet in methanol. The colonies were counted manually and plotted. For RNA interference, cells were transfected with respective siRNA for 48 h before being seeded for CFA.

For drug treatments, cells were continuously maintained in media containing different concentrations of respective drugs for 14 days before being stained and counted.

RT-qPCR

Total RNA isolation was performed using RNeasy kit (Qiagen-74104) as per manufacturer's protocol. 1000ng of total RNA was used for cDNA synthesis with the help of high-capacity cDNA synthesis kit (ABI- 4368814) as per manufacturer's protocol. RT-qPCR was performed in Bio-Rad CFX384 real-time system using iTaq universal SYBR green supermix (Bio-Rad- 172-5122) as per manufacturer's protocol and the results were quantitatively analyzed using CT values.

Cytogenetic analyses

Independent clones of $\mathit{Brca2}^{ko/ko\text{-}r}$ or $\mathit{Brca2}^{cko/ko\text{-}mi}$ mESCs cells were grown for 24hr and the cells were arrested in metaphase of mitosis by using colcemid (10 μg/ml, 15210-016, KaryoMAX) for 4 h. For drug treatments, cells were treated with 100nM olaparib (24 hr) or 200mM HU (8 hr) or 10nM camptothecin (24 hr) or 20ng/µl MMC (24 hr) or 0.6uM cisplatin (24 hr) before being arrested in metaphase. The cells were then fixed with methanol: acetic acid (3:1), stained with giemsa stain and visualized under microscope. Aberrations were blindly counted and quantified.

Western Blot and ChIP-Western Blot

Western Blot was carried out as described earlier (3). Protein extracts were prepared by cell lysis using RIPA lysis buffer (Millipore-20188) with cOmplete™, Mini Protease Inhibitor Cocktail (Millipore sigma-11836153001). Total proteins were estimated using Bio-Rad Protein Assay Dye Reagent Concentrate (Bio-Rad-5000006) and 20ug of protein extracts were resolved using 8-12% SDS-PAGE for 90min at 85V. Electro-transfer of proteins to nitrocellulose membrane was performed at 110V for 90min. Membrane was blocked using 5% non-fat milk in TBST and incubated with respective primary antibodies overnight at 4^0C. Next day, membranes were washed thrice using TBST, incubated with secondary antibody for 1h at RT and chemiluminescence was

detected using Cytiva Amersham™ ECL™ Prime Western Blotting Detection Reagent (Thermo-Fisher Scientic-45002401). For ChIP-IB, same protocol as ChIP-PCR was followed except antibody bound DNA-protein complexes were incubated with Benzonase nuclease (Millipore-70746) for 1h at 37^0C. Elution was performed by boiling the samples in lamelle buffer containing 0.5% β-mercaptoethanol for 20min at 95^0C before proceeding with Immunoblot for protein detection. RNase H treated samples acts as a negative control.

Proximity Ligation Assay (PLA)

Cells grown on chamber slides were treated with scrambled or respective siRNA's for 48h. Cells were fixed and permeabilized with methanol/acetone (1:1), washed twice with PBS and blocked using 3% BSA in PBST. The chamber slides were incubated with respective primary antibodies overnight at 4^0C. PLA was performed by using Duolink PLA fluorescence protocol. Cells were incubated with mouse PLUS (Sigma-Aldrich- DUO82001) and rabbit MINUS (Sigma-Aldrich-DUO82005) PLA probes for 1h at 37^0C. Ligation of probes were done at 37^0C for 1h using ligation buffer (Sigma-Aldrich- DUO82009) and ligase (Sigma-Aldrich- DUO82027). Amplication was performed at 37°C for 100 min using Amplification Buffer Far Red (Sigma-Aldrich-DUO82028) and Polymerase (Sigma-Aldrich-DUO82028). Slides were washed with wash buffers (Sigma-Aldrich-DUO82049) and stained with DAPI for 5min at RT. The slides were then mounted with prolong™ gold antifade reagent (ThermoFisher scientific-P36934), imaged using Zeiss LSM 710 confocal microscope and PLA foci per nuclei were counted manually using ImageJ.

***In vitro* binding assay**

The DNA and RNA oligos used in this study are listed in Supplemental Table 1. These oligos were used to form DNA: RNA hybrid (DR) or R-loop structures (DNA: RNA: DNA hybrid, DDR). The substrates were generated by mixing equimolar ratios of oligos and annealing was performed by

incubating in water bath at 95^{0}C and allowing to gradually cool to room temperature. The annealing of DR and DDR was confirmed by running 10% native polyacrylamide gel in 1X TBE buffer at 90V for 60min. Briefly, reactions were carried out at 37^{0}C for 60 min in nuclease buffer (25mM Tris-Cl, 1mM DTT, 5mM $MgCl_2$, 50mM KCl, 5mM ATP, 1X BSA) containing pure protein and substrates.

Chromatin fragments were diluted with ChIP dilution buffer (16mM Tris-HCl pH 8.0, 1.2mM EDTA, 1.1% Triton X-100, 167mm NaCl, 0.01% SDS) and immunoprecipitated with 2ug of IgG or S9.6 antibodies and 20ul of pre-blocked Dynabeads overnight at 4^{0}C with rotation. Next day, same protocol as ChIP-IB was followed and MLH1 antibody was used for immunoblotting.

Immunoprecipitation

Immunoprecipitation was performed as per manufacturer's protocol (Cell signaling technology, 9803). 1X Cell Lysis Buffer containing 20 mM Tris (pH 7.5), 150 mM NaCl, 1 mM EDTA, 1 mM EGTA, 1% Triton X-100, 2.5 mM Sodium pyrophosphate, 1 mM β-glycerophosphate, 1 mM Na_3VO_4, 1 µg/ml Leupeptin and 1X protease inhibitor cocktail was used for protein isolation on ice for 1hr. Protein concentration was estimated using Bradford assay and 0.5-1mg protein were used for immunoprecipitation using 1ug of respective primary antibody and 20ul of Protein A/G Dynabeads in 500ul of 1X Cell Lysis Buffer at 4^{0}C overnight in gentle rotor. Next day, the beads were washed 5 times with 500ul of 1X Cell Lysis Buffer using magnetic stand. Elution was done by boiling the samples at 95^{0}C for 30min in 20ul of lamelli buffer and the samples were analyzed by running 10% SDS PAGE.

Antibodies, Plasmids and Primers

Antibodies used for different experiment were follows. Immunofluorescence: γH2AX (07-164; Upstate, 1:500 dilution), S9.6 (MABE1095; Millipore; 1:1000 dilution); DNA Fiber Assay: anti-CldU (ab6326; Abcam, 1:500 dilution), anti-IdU (347580; BD Biosciences, 1:500 dilution); Immunoblot: β-actin (sc-1616; Santacruz Biotechnology, 1:10000 dilution), MLH1 (ab92312; Abcam, 1:1000 dilution), DNA2 (18727-1-AP; Proteintech, 1:1000 dilution), MRE11 (4895; Cell signaling technology, 1:5000 dilution), PARP1 (9532; Cell signaling technology, 1:5000 dilution), Histone H3 (4499; Cell signaling technology, 1:10000 dilution), MSH2 (2850; Cell signaling technology, 1:1000 dilution), MSH6 (5424; Cell signaling technology, 1:1000 dilution), CtIP (9201, Cell signaling technology, 1:5000 dilution), EXO1 (A302-639A; Bethyl laboratories, 1:2000 dilution), APE1 (ab92744; Abcam, 1:1000 dilution), ER-alpha (8644S; Cell signaling technology, 1:1000 dilution). DRIP: S9.6 (MABE1095; Millipore, 1 μg/IP), IgG (7076P2; Cell signaling technology, 1 μg/IP). ChIP: RNA pol II (05623; Millipore, 1 μg/IP), IgG (2729S; Cell signaling technology, 1 μg/IP).

Plasmids used in this study: Dna2_OMu22714D_pcDNA3.1+/C-(K)-DYK (Genscript); Mlh1_ OMu19454_pcDNA3.1+/C-(K)-DYK (Genscript); Fen1_OMu15830_pcDNA3.1+/C-(K)-DYK (Genscript). SMARTvector Inducible Mouse Mlh1 mEF1a-TurboGFP shRNA (V3SM11256-03EG17350, Horizon Discovery Bioscience); SMARTvector Inducible Non-targeting mEF1a-TurboGFP (VSC11654, Horizon Discovery Bioscience).

TCGA Dataset Analysis

TCGA dataset analysis was carried out using cBioportal (http://cbioportal.org). For survival and expression analysis, several different cancer datasets were analyzed using Cbioportal by querying the command (*MLH1* low: *MLH1*: exp<-1; *MLH1* high: *MLH1*: exp>1). Similarly, breast cancer

datasets (TCGA, Firehose legacy) were used to analyze different MMR genes such as *MLH3, MSH2* and *PMS2* on patient survival by querying the command (*MLH3* low: *MLH3*: exp<-1; *MLH3* high: *MLH3*: exp>1 and so on). *BRCA2* low and high samples were retrieved by querying the command (*BRCA2* low: *BRCA2*: exp<-0.75; *BRCA2* high: *BRCA2*: exp>0.75). These samples were analyzed with respect to *MLH1* and *MLH3* Low or high samples to analyze patient survival. For survival analysis, data from three different breast cancer datasets (TCGA, Nature; TCGA, Cell and TCGA, Firehose legacy) were pooled together and survival graph was plotted using GraphPad prism. Note: TCGA, Nature; TCGA, Cell and TCGA, Firehose legacy datasets could have some overlapping samples. For fraction genome alterations and phosphorylation status of DNA damage markers, we queried the command (*MLH1* low: *MLH1*: exp<-0.5; *MLH1* high: *MLH1*: exp>0.5; *BRCA2* low: *BRCA2*: exp<-0.75; *BRCA2* high: *BRCA2*: exp>0.75).

We defined the cut-offs based on Z-score wherein we used the command (for survival analysis- *MLH1*: exp<-1 for *MLH1* low and *MLH1*: exp>1 for MLH1 high samples); (for fraction genome altered- *MLH1*: exp<-0.5 for *MLH1* low and *MLH1*: exp>0.5 for MLH1 high samples); (for all analysis- *BRCA2*: exp<-0.75 for *BRCA2* low and *BRCA2*: exp>0.75 for *BRCA2* high samples) and (for all analysis- *ESR1*: exp<-0.5 for *ESR1* low and *ESR1*: exp>0.5 for *ESR1* high samples). Command values are Z-score values wherein 0 is the median value. As per Cbioportal guidelines, we picked these thresholds based on the overall *MLH1*, *BRCA2* and *ESR1* expression (using Plots tab) and compared survival data between expression high and low groups.

RNA sequencing and Analysis

Total RNA from *Brca2*$^{ko/ko-r}$ and *Brca2*$^{cko/ko-mi}$ mESCs was isolated using RNAeasy mini kit (Qiagen) and the integrity of RNA was analyzed using Bioanalyzer. 250ng of total RNA was using for library preparation using TruSeq Stranded mRNA library prep according to manufacturer's

instructions (Illumina, USA). 250ng or 500ng of total RNA was used as the input to an mRNA capture with oligo-dT coated magnetic beads. The mRNA was fragmented, and cDNA synthesis was performed using random primers. The resulting double-stranded cDNA was used as the input to a standard Illumina library prep with end-repair, adapter ligation, PCR amplification. The final purified library product is then quantitated by qPCR. The libraries were pooled and sequenced on a HiSeq4000 with 2x150bp read length using pair-end run. Sequencing was performed following the manufacturer's instructions.

The HiSeq Real Time Analysis software (RTA 1.18.64) was used for processing raw data files, the Illumina bcl2fastq2.17 was used to demultiplex and convert binary base calls and qualities to fastq format. The sequencing reads were trimmed adapters and low-quality bases using Cutadapt (version 1.18). The trimmed reads were mapped to mouse reference genome (mm10) and GENCODE annotation M9. The mapping was using STAR aligner (version 2.7.0f) with two-pass alignment option. RSEM (version 1.3.1) was used for gene and transcript quantification based on GENCODE M9 GTF file.

Loss of conditional allele of *Brca2* was confirmed by Integrative Genome Viewer (IGV) from Broad Institute. For analysis, genes that showed >2fold expression were considered upregulated and genes that showed <0.2 were considered down regulated. The genes that are altered were used for Pathway and Gene ontology analysis and the analysis were performed using DAVID software (https://david.ncifcrf.gov). Heat map clustering was performed using Heatmapper (http://www.heatmapper.ca).

Whole Genome Sequencing and Analysis

The TruSeq Nano DNA Library prep kit (Illumina, 15041110 D) was used to prepare samples for whole genome sequencing. The genomic DNA was fragmented to approximately 550 bp insert

size on Covaris. 75ng or 100ng of DNA was used as input for TruSeq Nano DNA libraries preparation according to manufacturer's protocols (Illumina, USA). The quality of all libraries was assessed by Tape Station instrument (Agilent) in combination with the High Sensitivity DNA Kit (Agilent, 5067-4626). The concentration of the TruSeq Nano DNA libraries for WGS was measured by qPCR. The whole genome libraries were pooled together and sequencing on a NovaSeq 6000 instrument at 2x150bp read length using the S2 configuration. Sequencing was performed following the manufacturer's instructions.

The sequencing run was demultiplexed using Illumina Bcl2fastq v2.20 software tool. The raw fastq files were used as input to run DRAGEN v3.9.5 (Illumina, USA) for mapping and variant calling. The mouse genome sequence mm10 (GRCm38) was used as reference. DRAGEN was run for each tumor and normal pairs using the somatic variant calling mode to call the structural variants and SNVs. For normal samples, the germline joint genotyping was used to call variants. Respective controls (for example, C57_Csi, $BRCA2^{Y3308X\_cvec}$ and $BRCA2^{\mathrm{Y3308X}}$) were implemented as the control for the structural variation calling for each group. For each group, we defined unique SV occurred in each sample as two break ends within 1kb regions across the genome for the same SV type. Circlize package was used to showcase the circos plot for each sample (4). We annotated the mutagenesis-related epigenomic (MRE) states and compared the MRE states with chromatin states identified by chromHMM (5) that are relevant for transcription regulation (6). This was used to combine genomic and epigenomic features that are not independent, and synergistically impact DNA damage and repair (6). In this analysis, we compared the proportions of the MRE states in each clone using deconstructSigs (7) between $Brca2^{ko/ko}$ and $Brca2^{cko/ko}$ mES clones.

Animal Studies

For mouse allograft studies, animals were randomly assigned into four groups (n=8-10). No selection criteria were applied to select the animals in each group. Doxycycline was administered by feeding mice with doxycycline-containing food pellets (625ppm, Special Diet Services, Witham, England) and replenished every week. Two groups were fed with doxycycline feed and Two groups were fed with control feed for 3 days prior to the start of the experiment. $2x10^6$ shControl and sh*Mlh1* KB2P1.21 cells in 200ul of L15 medium were used for injection into 6- to 8-week-old male athymic nude mice. Body weight of the animals was measured at Day5. The tumor volume was measured every 2days starting from Day13 using digital caliper. Tumor volume was calculated in mm^3 using $(L X W^2)/2$.

For genetic crossing experiments, $Brca2^{L2431P/L2431P}$ mice and $Mlh1^{ko/+}$ mice were used. Generation of $Brca2^{L2431P/L2431P}$ mice was previously reported from our laboratory (8). We have generated the $Mlh1^{ko/+}$ mice using cryopreserved sperm from $Mlh1^{ko/+}$ animal (NCI repository, Strain name: B6.129-$Mlh1^{tm1Rak/Nci}$, Strain code: 01XA2). $Mlh1^{ko/+}$ mice were inter-crossed with $Brca2^{L2431P/L2431P}$ mice to obtain mice of nine different genotypes. All animal procedures reported in this study that were performed by NCI-CCR affiliated staff were approved by the NCI Animal Care and Use Committee (ACUC) and in accordance with federal regulatory requirements and standards. All components of the intramural NIH ACU program are accredited by AAALAC International.

PDX models

The PDX models were obtained from Dr. Michael T. Lewis, Baylor College of Medicine and the Patient-Derived Xenograft and Advanced In Vivo Models Core. Pathology and genetic characterization of these models is available at https://pdxportal.research.bcm.edu/pdxportal (9). PDXs (BCM5097, -5471, -4888, -3887) were propagated in NOD/SCID/ILIIrg$^{-/-}$ (NSG) mice

(NCI) and tumors harvested as described (10). Experiments were performed with transplant generations 9–10. Tumor volumes were calculated as $V = (W^2 \times L)/2$.

Organoid generation

The organoids were generated by following the protocol from Sachs et al (11) with few modifications. The tumors from BCM5097 (transplant generation 9-10) were washed and with minced with 5ml of Advanced DMEM/F12+Glutamax containing 10mM HEPES (AdDF+++) and antibiotics and digested in 4ml of BC organoid media containing 2mg/ml of collagenase on orbital shaker at 37^0C for 2h. The cell suspension was strained using 100um filter and centrifuged at 400g. Visible red blood cells were lysed by incubating the cell pellet with ammonium chloride solution for 5min at RT. The cells were washed with AdDF+++ by centrifuging at 400g and the cell pellet were resuspended in 10mg/ml cold cultrex growth factor reduced BME type 2 (Trevigen, 3533-010-02). 40ul of BME-cell suspension were solidified on prewarmed 24-well low attachment culture plate at 37^0C for 20min. Upon gelation, 400ul of BC organoid medium was added and plates were maintained at 37^0C for organoid formation. Media was changed every 4 days and passaging of organoids were done after 10 days by dissociating the organoids using TrypLE express (Invitrogen, 12605036). Alternatively, the cell pellet was resuspended in 2-10% Matrigel for some experiments and new BC organoid media was added every 3days with or without drugs.

Genotyping

Genotyping was done using the standard protocol (12). Briefly, genomic DNA from tail biopsies were isolated by lysing the cells using ProteinaseK at 55^0C overnight. 50-100ng of tail DNA was used for PCR using Taq DNA polymerase. Primers used for genotyping are listed in Supplemental Table 1. For Mlh1 genotyping, *Mlh1* Forward 1 and *Mlh1* Reverse 2 was used to confirm the

knockout (amplicon size:500bp) and *Mlh1* Forward 1 and *Mlh1* Reverse 3 was used to confirm the wildtype Mlh1 (amplicon size:350bp). For L2431P locus genotyping, *Brca2 L2431P* Forward and *Brca2 L2431P* Reverse primers were used. Amplicon size of 204bp corresponds to wildtype and Amplicon size of 320bp corresponds to *L2431P* mutant.

Statistical analysis: Blocks of samples and P-value adjustment for multiple pairwise comparisons using R-studio

To perform P-value adjustment for multiple comparisons between pairs of samples, we divided the samples into groups (here termed "blocks") using concepts from basic graph theory. A group of statistical samples can be viewed as an undirected graph, where the nodes correspond to the samples and the edges correspond to the pairwise comparisons performed. In our analysis, P-value adjustment for multiple comparisons was performed separately on each block comprising ≥3 samples, and a block of samples was defined as a group of samples whose corresponding graph is ***connected***. By definition, a connected graph is a graph where every node is reachable from every other node (i.e., for every pair of nodes, there is a path connecting them). Also, we assume that our connected graphs are maximal in the sense that they are not part of a connected graph that has more nodes.

Our approach can be justified as follows. The simplest situation where a P-value adjustment for multiple comparisons is needed is when there are 2 samples (say, #1 and #3), both of which are compared with a third sample (call it #2). (The P-value adjustment is needed because one sample – sample #2 – is involved in more than one comparison.) Imagine now that there is a sample #4 that is compared with sample #3. Then, the samples #4, #3, #2 correspond to the simplest situation requiring a P-value adjustment – just like the samples #3, #2, and #1. Note that both sample #4 and sample #1 here need to be analyzed together with samples #2 and #3. Therefore, all

these four samples need to be analyzed together, and the corresponding P-values need to be adjusted together (i.e., the multiple-comparisons adjustments need to be performed for all the individual P-values after they are computed for the pairwise comparisons described). We can extend this argument to the case when there is also a sample #5 that is compared with sample #4, and a sample #6 that is compared with sample #5, etc. In other words, the samples in any block whose graph is a path should be analyzed, and P-value-adjusted, together. Now, consider the general case of a block of samples (whose corresponding graph, call it graph G, is connected). Select an arbitrary node in graph G, call it node A. Because the graph is connected, its every node is reachable from node A, i.e., there is a path between that node and node A. Therefore, every sample corresponding to a node in graph G needs to be analyzed together with the sample corresponding to node A. Therefore, all the samples corresponding to graph G need to be analyzed together, and all the raw, individual P-values for the pairwise comparisons corresponding to the edges in graph G need to be adjusted for multiple comparisons together, as one group.

Supplemental Figures

Supplemental Fig. S1. Data supporting the enrichment of mismatch repair pathway and no effect of *Mlh3* or *Pms2* on viability of *Brca2$^{ko/ko-r}$* mES cells.

A) Representative Southern blots showing genotyping of HAT resistant mESC to identify *Brca2$^{ko/ko-r}$* clones obtained from DMSO (left panel, top) and two independent mirin treated *Brca2$^{cko/ko}$* cells (left panel, middle and bottom). Upper band (4.8kb) corresponds to conditional allele of *Brca2* and lower band (2.2kb) corresponds to knockout allele of *Brca2*. Stars represent the rescued *Brca2$^{ko/ko-r}$* clones. Quantitation of the percentage rescue (based on number of HAT resistant clones that were confirmed to be *Brca2$^{ko/ko}$*) of *Brca2$^{ko/ko-r}$* clones (n=4 experiments) (right panel). B) RNA seq analysis using IGV viewer (Broad Institute) showing the deletion of exon 11 from conditional allele of Brca2 in *Brca2$^{ko/ko-r}$* (n=3 clones) and *Brca2$^{cko/ko-mi}$* (n=1 clone). C) RNA seq analysis using IGV viewer (Broad Institute) showing the expression of MRE11 in *Brca2$^{ko/ko-r}$* (n=3 clones) and *Brca2$^{cko/ko-mi}$* (n=1 clone). D) Immunoblot analysis showing the expression of MRE11 in *Brca2$^{ko/ko-r}$* (n=2 clones) and *Brca2$^{cko/ko-mi}$* (n=1 clone). E) KEGG pathway analysis using DAVID database showing the pathway enrichment in *Brca2$^{ko/ko-r}$* clones. Each dot represents the independent *Brca2$^{ko/ko-r}$* clones (n=15). F) Heatmap showing the expression of mismatch repair pathway genes in *Brca2$^{ko/ko-r}$* (n=22 clones) and *Brca2$^{cko/ko-mi}$* (n=2 clones). G) Immunoblot analysis showing the expression of MLH1, MSH2 and MSH6 in *Brca2$^{ko/ko-r}$* (n=1 clones) and *Brca2$^{cko/ko-mi}$* (n=2 clones). β-actin was used as control. H) RT-qPCR analysis showing the knockdown of *Mlh1* and *Msh2* in *Brca2$^{ko/ko-r}$* (n=3) and *Brca2$^{cko/ko-mi}$* (n=1) clones shown in Fig. 1E. I) Colony formation assay upon Control, *Mlh3, Pms2* and/or combination of *Mlh3 and Pms2* silencing in *Brca2$^{ko/ko-r}$* (n=3) and *Brca2$^{cko/ko-mi}$* clones (n=1) (left panel). Quantitation of (I), showing the normalized cell viability of Control, *Mlh3, Pms2* and/or combination of *Mlh3 and Pms2* silenced *Brca2$^{ko/ko-r}$* (n=3) and *Brca2$^{cko/ko-mi}$* clones (n=1) (right panel). Data were analyzed using unpaired, two-tailed student's t-test (A,I) and unpaired, two-tailed student's t-test with Holm-sidak multiple-comparison test (H). *p<0.05; **p<0.01; ***p<0.001.n.s: non-significant.

Supplemental Figure 1

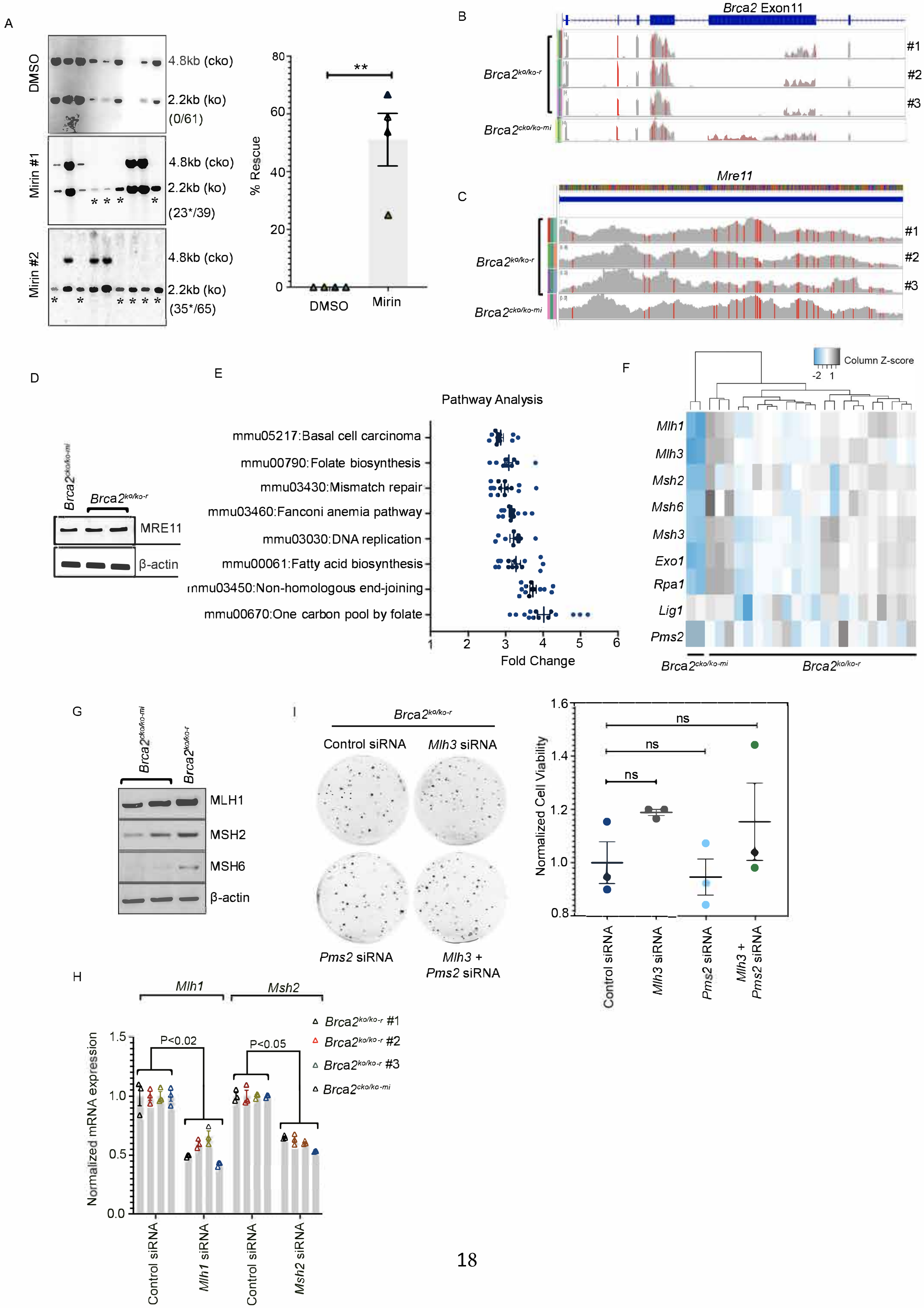

Supplemental Fig. S2.
MLH1 but not MSH2 or MLH3 rescues *Brca2* null ES cells.

A) Immunoblot showing the knockdown of MLH1 in *Mlh1* silenced *Brca2*$^{cko/ko}$ cells used in (B) and (C). B) Pictorial representation of rescue assay in control and/or *Mlh1* silenced *Brca2*$^{cko/ko}$ cells. After the confirmation of MLH1 knockdown (48h later), mirin pretreatment was given for 3h, followed by electroporation of Cre to knockout conditional *Brca2* allele in *Brca2*$^{cko/ko}$ cells. 100nM Olaparib was used to identify the dropout of rescued colonies. C) Quantitation of the number of surviving colonies in rescue assay from (B) after treating with or without Olaparib (100nM) for 96hr in control and/or *Mlh1* silenced *Brca2*$^{cko/ko}$ cells. Data are from three independent experiments. D) Southern blot confirmation from (C) showing the dropout of *Brca2*$^{ko/ko\text{-}r}$ rescued clones in mirin pretreated Control or *Mlh1* silenced *Brca2*$^{cko/ko}$ cells upon exposure to DMSO or Olaparib (100nM). E) Immunoblot analysis showing the knockdown confirmation of MLH1 and MSH2 in *Brca2*$^{cko/ko}$ cells (Left panel). RT-qPCR analysis showing the knockdown confirmation of *Mlh3* in *Brca2*$^{cko/ko}$ cells (Right panel). These cells after knockdown for MLH1, MSH2 and MLH3 were used in (F). F) Quantitation of dropout of *Brca2*$^{ko/ko\text{-}r}$ rescued clones in Control or *Mlh1* or *Msh2* or *Mlh3* silenced *Brca2*$^{cko/ko}$ cells upon exposure to DMSO or Olaparib. Colonies were counted manually, and plotted. Experiments were repeated two times, and all experiments were performed in duplicates. Data are presented as mean±SEM for (F). Data were analyzed using paired, two-tailed student's t-test (C, F) and unpaired, two-tailed student's t-test (E). *p<0.05; **p<0.01; ***p<0.001.n.s: non-significant.

Supplemental Figure 2

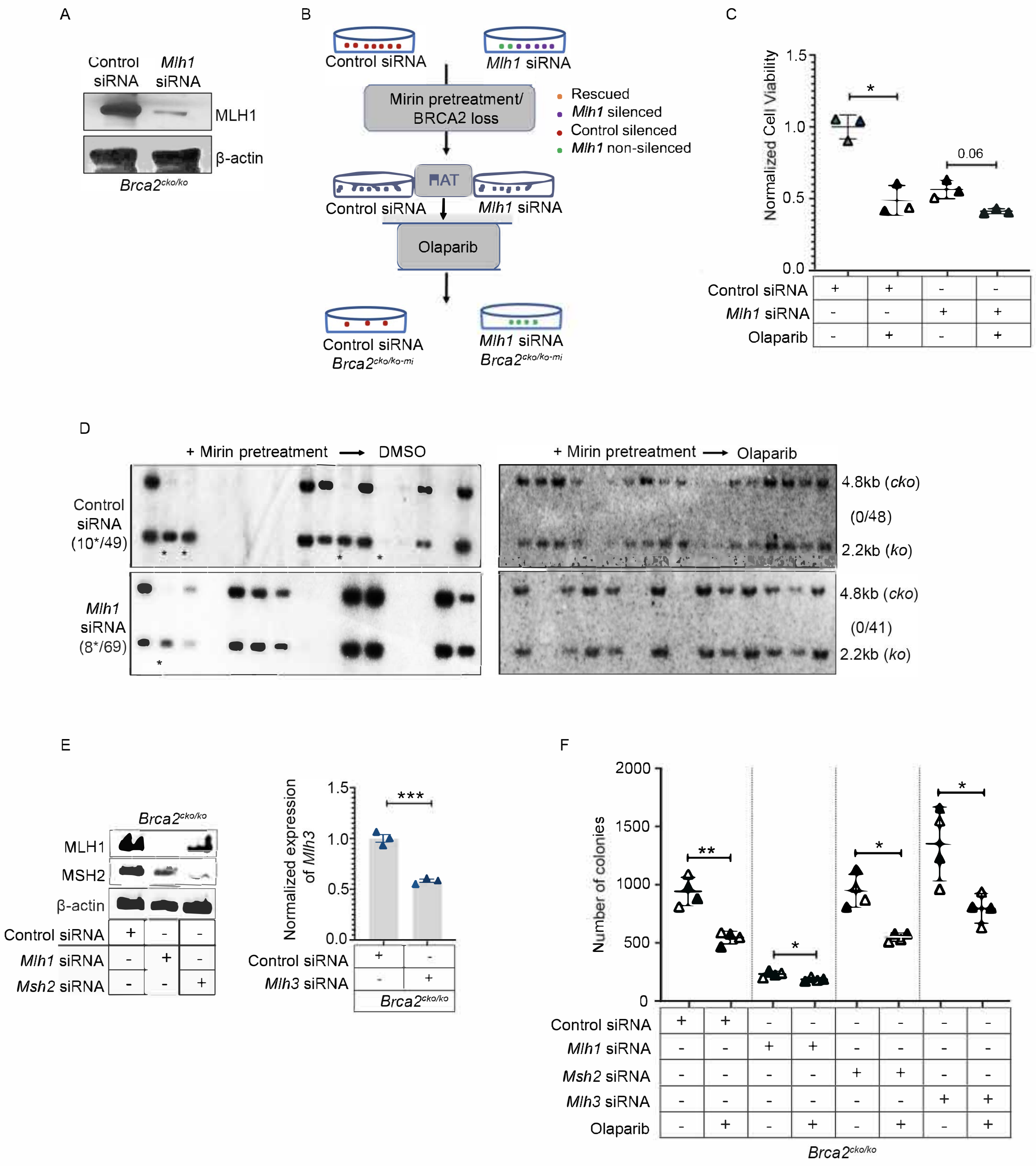

Supplemental Fig. S3.

MLH1 affects the patient survival differentially in breast, colon and other cancer types

Kaplan-Meier analysis showing the survival status of patients from *MLH1* High vs *MLH1* Low breast cancer patient samples (A) (TCGA, Firehose legacy) and (B) (TCGA, Cell) obtained from TCGA breast cancer datasets. Kaplan-Meier analysis showing the survival status of patients from High vs Low *MSH2* (C) or *MLH3* (D) or *PMS2* (E) breast cancer patient samples obtained from TCGA breast cancer datasets (TCGA, Firehose legacy). Kaplan-Meier analysis showing the survival status of patients from *MLH1* High vs *MLH1* Low obtained from different TCGA cancer datasets (such as Colorectal (F), Stomach (G), Acute myeloid leukemia (H), Cervical (I), Skin (J), Bladder (K), Liver (L), Thyroid (M), Ovarian (N) and Prostate (O). P) Representative images showing the IR induced RAD51 foci and γH2AX in $Brca2^{ko/ko\text{-}r}$ and $Brca2^{cko/ko\text{-}mi}$ clones. Data were analyzed using Log-rank (Mantel-Cox) test (A-O). *p<0.05; **p<0.01; ***p<0.001.n.s: non-significant.

Supplemental Figure 3

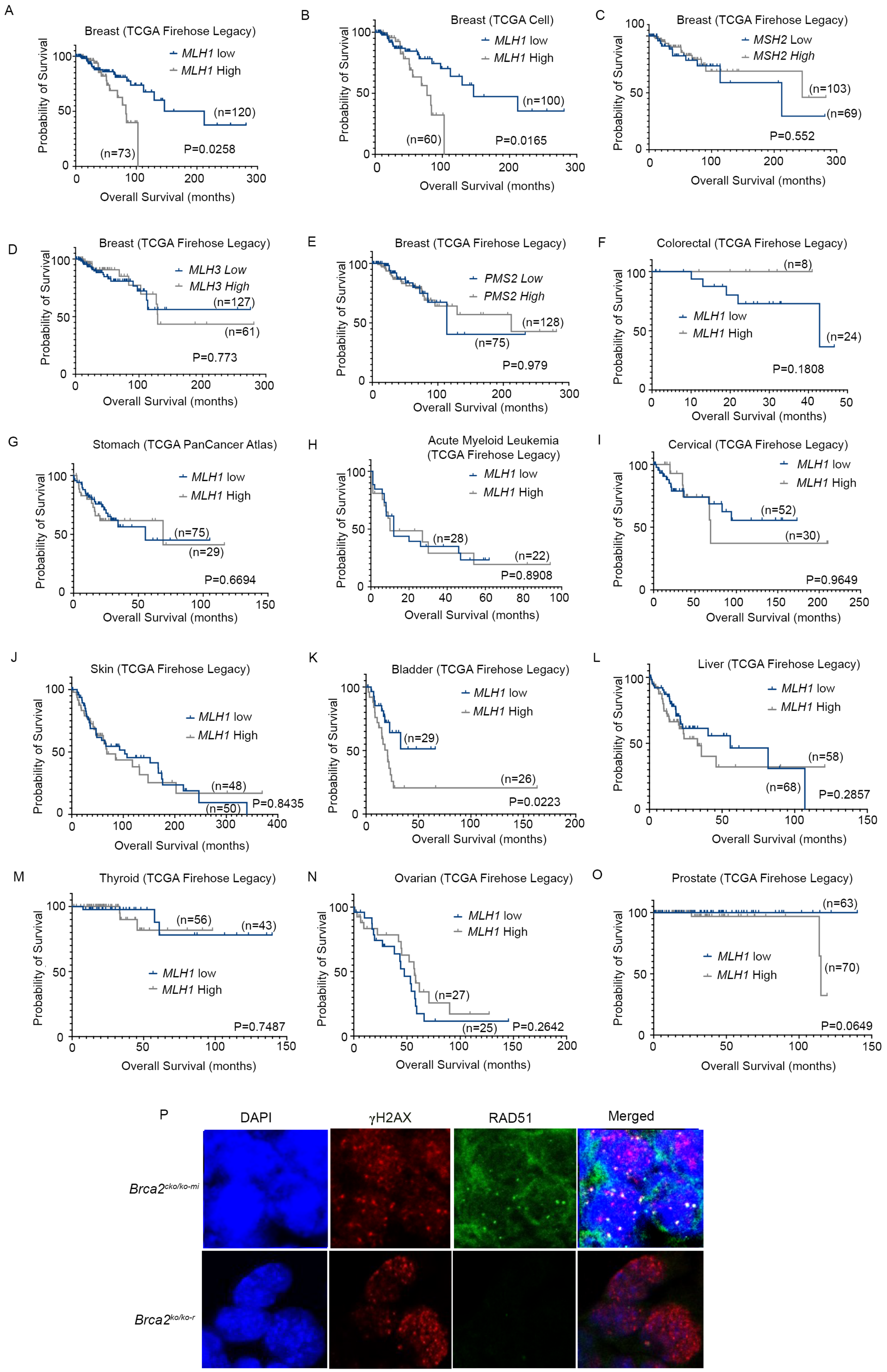

Supplemental Fig. S4.

High RF speed in *Brca2$^{ko/ko-r}$* is promoted by MLH1, but not by MLH3 or MSH2

A) Experimental procedure depicting the EdU labeling for 1 hour, followed by click chemistry and immunofluorescence (Left Panel). Immunofluorescence showing the intensity of EdU in *Brca2$^{ko/ko}$*(n=3) and *Brca2$^{cko/ko}$* (n=2) clones (Right Panel). B) Quantitation of mean intensity of EdU from (A) using ImageJ. Atleast 100 nuclei were measured for each sample from 3 independent experiments. C) Experimental procedure depicting the EdU labeling for 1h, followed by click chemistry and flow cytometry (Left Panel). Flow cytometric analysis of mean intensity of EdU (n=3 biological replicates) and γH2AX (n=3 biological replicates) in EdU positive and EdU negative *Brca2$^{ko/ko-r}$* and *Brca2$^{cko/ko-mi}$* cell populations (right panel). D) RT-qPCR analysis showing the knockdown confirmation of *Mlh1, Mlh3* and *Msh2* in *Brca2$^{ko/ko-r}$* and *Brca2$^{cko/ko-mi}$* clones. E) Quantitation of RF speed marked by dual track length in *Brca2$^{ko/ko-r}$*, *Brca2$^{cko/ko-mi}$* and *Brca2$^{cko/ko}$* clones upon silencing *Mlh1, Msh2* and *Mlh3*. n>150 nuclei from three independent experiments were measured using ImageJ and quantified (Right panel). Representative images are shown in left panel. F) Representative immunoblot showing the level of MLH1 on EdU labelled active RFs by iPOND assay in *Brca2$^{ko/ko-r}$* clones. RFs were labelled with EdU for 10min. Click chemistry without Biotin azide was used as a negative control. Histone H3 was used as an internal control. Experiments were performed three times with similar results. Data are presented as mean±SEM for (B, C, E). Data were analyzed using unpaired, two-tailed student's t-test (B, C, E) and Wilcoxon's rank sum test (C). *p<0.05; **p<0.01; ***p<0.001; ****p<0.0001. n.s: non-significant.

Supplemental Figure 4

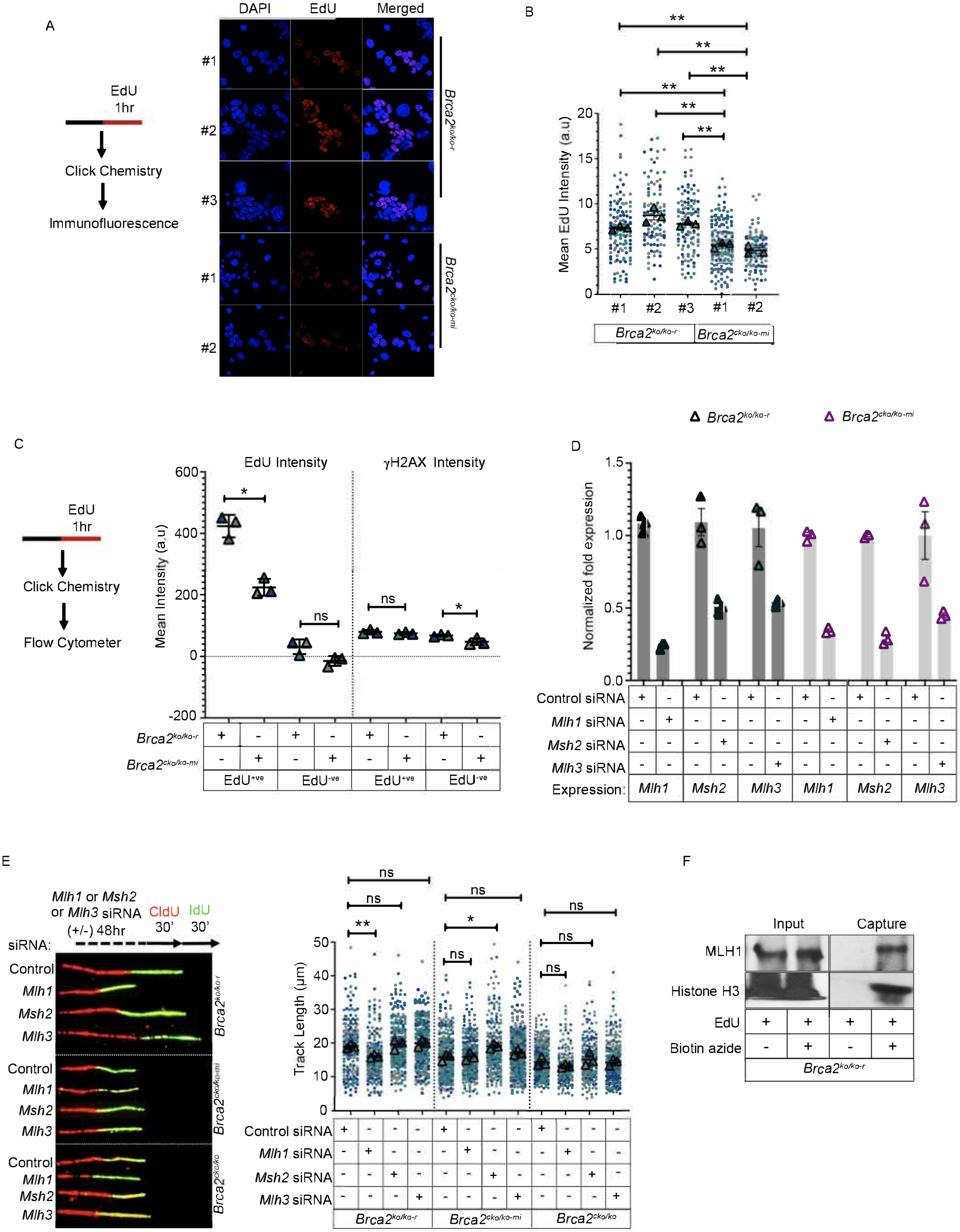

Supplemental Fig. S5.

High Fork restart in *Brca2*$^{ko/ko-r}$ is promoted by MLH1.

A) S1 nuclease assay showing the replication gaps in Brca*2*$^{ko/ko-r}$ (n=2) and *Brca2*$^{cko/ko-mi}$ (n=1) clones in unperturbed (Control) and perturbed (10µM Olaparib treatment for 2hr) conditions. Reduction in tract length upon S1 nuclease treatment is read as replication gaps. B) Representative images of DNA fibers showing fork restart in *Brca2*$^{ko/ko-r}$ (n=2) and *Brca2*$^{cko/ko-mi}$ clones (n=1). Right panel shows the quantitation of fork restart from (B) using ImageJ. Each dot in the graph represents the percentage restarted forks per individual field (n>20 fields from 3 independent experiments (n>1000 fibers). C) Representative images of DNA fibers showing fork restart in control and *Mlh1* silenced *Brca2*$^{ko/ko-r}$ and *Brca2*$^{cko/ko-mi}$ clones. Restarted forks are marked by white arrows. RFs labelled with CldU for 30min were stalled by hydroxyurea (HU) treatment for 3hr and restart was marked by labelling the nascent DNA with IdU for 30min. Right panel shows the quantitation of fork restart from (C) using ImageJ. Each dot in the graph represents the percentage restarted forks per individual field (n>1000 fibers). The experiment was repeated 3 times and data are presented as mean±SEM. D) Flow cytometry analysis showing the fork restart in *Brca2*$^{ko/ko-r}$ and *Brca2*$^{cko/ko-mi}$ clones upon silencing *Mlh1*. RFs were stalled by 4mM HU for 3 hr and fork start was analyzed by labeling the cells with EdU for 1h. Percentage of EdU positive cells after release into fresh media from HU media is a direct measure of fork restart. No EdU was used as a negative control. The experiment was repeated 3 times with similar results and representative images from one independent experiment is depicted. Data are presented as mean±SEM for (A, B, C). Data were analyzed using paired, two-tailed student's t-test (A) and unpaired, two-tailed student's t-test (B,C). *p<0.05; **p<0.01; ***p<0.001.n.s: non-significant.

Supplemental Figure 5

A

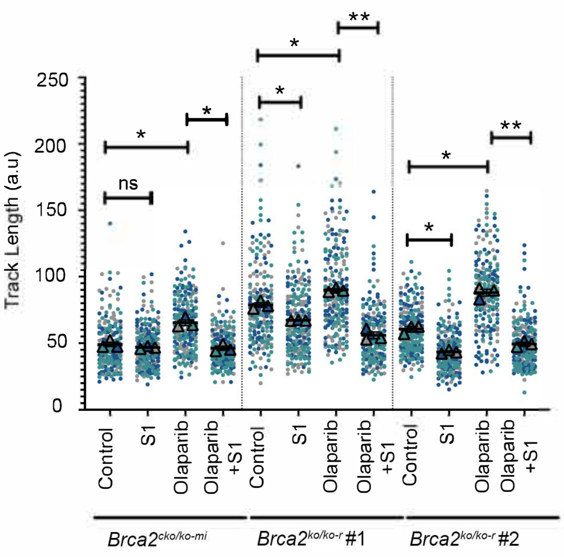

D

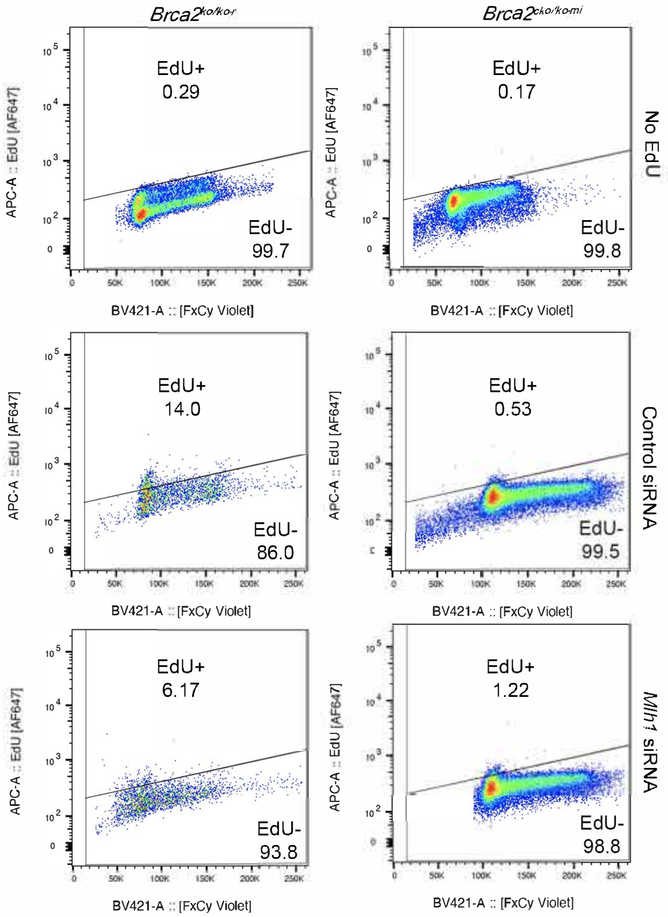

B

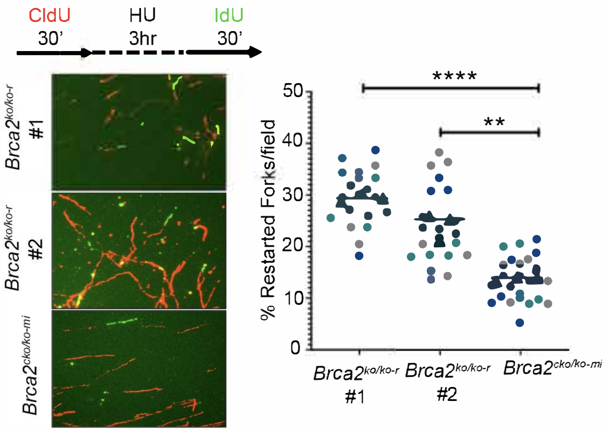

C

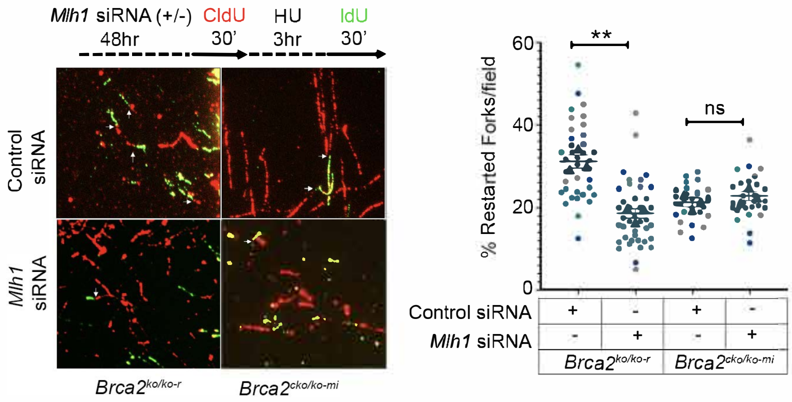

Supplemental Fig. S6.

MLH1 protects stalled reversed RFs from DNA2.

A) Quantitation of DNA fibers showing the IdU/CldU tract ratios of DMSO treated *Brca2*$^{ko/ko\text{-}r}$ (n=2 clones) and *Brca2*$^{cko/ko\text{-}mi}$ clones. BRCA2 variant, *BRCA2*^{Y3308X} (known to have fork degradation) was used as a positive control. This panel acts as a control for Fig. 2B. B) Quantitation of DNA fibers showing the IdU/CldU tract ratios in HU treated Control or *Mlh1* or *Msh2* silenced *Brca2*$^{cko/ko\text{-}mi}$ (n=1) and *Brca2*$^{ko/ko\text{-}r}$ (n=2) clones. C) Knockdown confirmation of MLH1 in KB2P1.21R2 and KB2P1.21 by Immunoblot (left panel). Quantitation of DNA fibers showing the IdU/CldU tract ratios in Control or *Mlh1* silenced KB2P1.21 R2 and KB2P1.21 mouse mammary cancer cells (Right Panel). D) Quantitation of IdU/CldU tract ratios of HU treated Control or *Hltf* or *Smarcal1* or *Zranb3* silenced *Brca2*$^{cko/ko\text{-}mi}$ mESCs. E) Knockdown confirmation of nucleases, EXO1, MLH1, CtIP, DNA2 and APE1 in U2OS cells by Immunoblot. F) Quantitation of DNA fibers showing the IdU/CldU tract ratios of HU treated Control or *MLH1* silenced U2OS upon *EXO1, CtIP, DNA2, MUS81* and *APE1* knockdown. G) Quantitation of DNA fibers showing IdU/CldU tract ratios of HU treated Control or *MLH1* silenced mirin treated and/or untreated U2OS. Data were analyzed using unpaired, two-tailed student's t-test and Wilcoxon's rank sum test (A, C); unpaired, two-tailed student's t-test (B, D, F, G). *$p<0.05$; **$p<0.01$; ***$p<0.001$; ****$p<0.0001$. n.s: non-significant.

Supplemental Figure 6

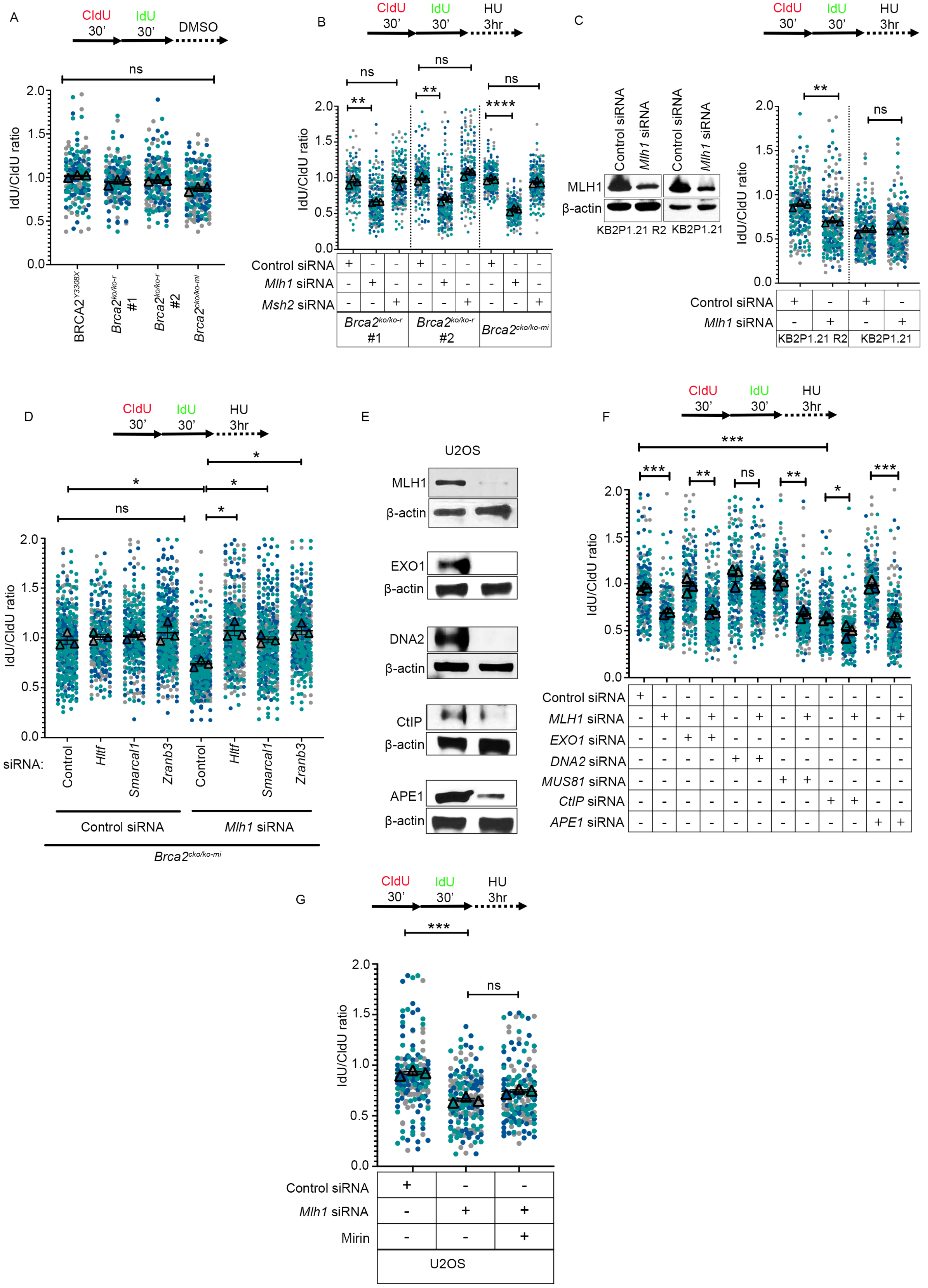

Supplemental Fig. S7.

DNA2 depletion rescues the MLH1 loss induced RF degradation in BRCA2 proficient MEFs and mouse mammary tumor cells.

A) RT-qPCR analysis showing the knockdown confirmation of *Mlh1* and *Dna2* in *Brca2*$^{ko/ko\text{-}r}$ clones. B) Quantitation of DNA fibers showing the IdU/CldU tract ratios of DMSO treated control or *Mlh1* or *Dna2* silenced *Brca2*$^{cko/ko\text{-}mi}$ or *Brca2*$^{ko/ko\text{-}r}$ or *Brca2*$^{cko/ko}$ clones. This panel acts as a control for Fig. 3A. C) Quantitation of IdU/CldU tract ratios of control or *Dna2* silenced in *Mlh1*$^{ko/+}$(n=1) or *Mlh1*$^{ko/ko}$(n=2) MEFs. D) Quantitation of DNA fibers showing the IdU/CldU tract ratios of HU treated Control or *Mlh1* or *Dna2* silenced KB2P1.21R2 treated with or without Mirin. E) Quantitation of DNA fibers showing the IdU/CldU tract ratios of DMSO treated Control or *Mlh1* or *Dna2* silenced KB2P1.21R2 treated with or without Mirin. All experiments were repeated three times and fibers n>150 pooled from three independent experiments were used to plot the graph. Data are presented as mean±SEM. Data were analyzed using unpaired, two-tailed student's t-test (A-E). *p<0.05; **p<0.01; ***p<0.001; ****p<0.0001. n.s: non-significant.

Supplemental Figure 7

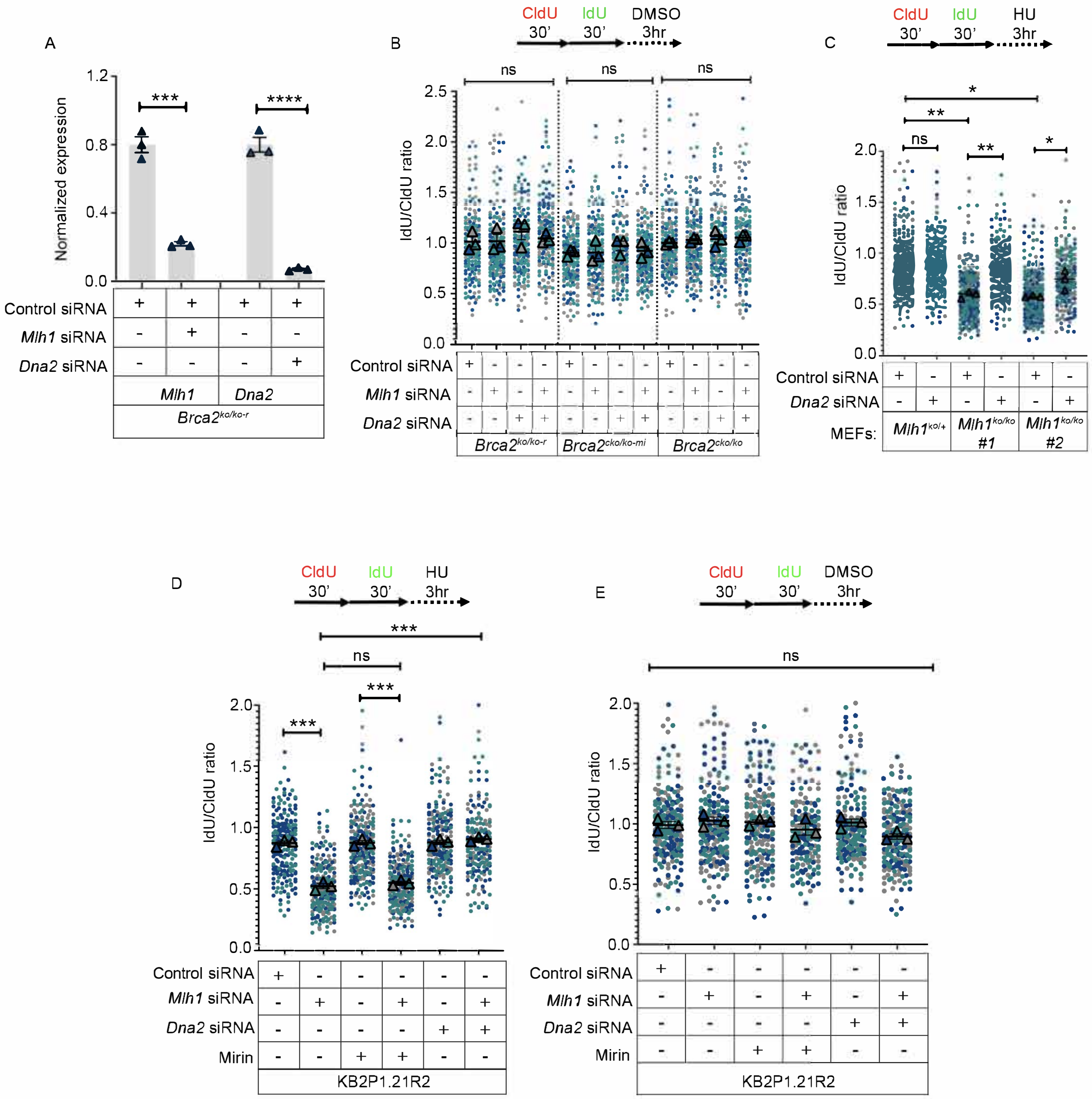

Supplemental Fig. S8

MLH1 reduces FEN1 mediated processing of long 5' DNA flaps of Okazaki fragments.

A) Quantitation of DNA fibers showing the IdU/CldU ratios of HU treated Control or *Fen1* over expressing KB2P1.21 cells. B) Quantitation of DNA fibers showing the IdU/CldU tract ratios of HU treated Control or *Mlh1* or *Fen1* silenced *Brca2*$^{ko/ko-r}$ clones. C) Pictorial representation of ChIP-immunoblot using S9.6 antibody for ChIP and Immunoblot for MLH1 (left panel). ChIP-Immunoblot showing the level of MLH1 on *invitro* developed DNA: RNA hybrid (DR) pulled down with IgG or S9.6 (right panel). MLH1 pure protein was used for this assay. IgG pulldown was used as a negative control. D) Immunoblot showing physical interaction between FEN1 and MLH1. MLH1 was present in KB2P1.21 cell lysates immunoprecipitated using FEN1 antibody. IgG was used as control. 5% protein lysates were used as input. Experiment was repeated two times with similar results and representative blot from one independent experiment is shown in (C, D). E) *In vitro* nuclease assay showing the 60nt DNA flap processing of MLH1(varying concentration of 250ng and 500ng used), FEN1(fixed concentration of 50ng used) alone and in combination in reaction for 15min at 37^0C. Along with the flap's, different sized products are shown (representative blot). F) *In vitro* nuclease assay showing the 30nt DNA flap processing of 250ng MLH1, 50ng FEN1 alone and in combination in reaction for 15min at 37^0C. Along with the flap's, different sized products are shown (representative blot). Experiment was repeated three times with similar results for (E) and (F). G) Immunoblot showing the knockdown confirmation of MLH1 and FEN1 for (Fig 3G). H) Immunoblot showing the knockdown confirmation of MLH1 and FEN1 for (Fig 3H). I) Quantitation of DNA fibers showing the IdU/CldU tract ratios of HU treated Control or *Mlh1* or *Fen1* or *Smarcal1* silenced *Dna2* over-expressing KB2P1.21clones. J) RT-qPCR showing the normalized expression levels of *Mre11* in Control or *Brca2* or *Dna2*

silenced cells. K) TCGA dataset analysis (TCGA, Firehose Legacy) showing the protein expression levels (RPPA) of MRE11 in *DNA2* median or *DNA2* low breast cancer patient samples. The experiments were repeated three times for (A, B, I) and fibers n>150 pooled from each independent experiment were used to plot the graph. Data are presented as mean±SEM. Data were analyzed using unpaired, two-tailed student's t-test (A, B, I, J). *p<0.05; **p<0.01; ***p<0.001; ****p<0.0001. n.s: non-significant.

Supplemental Figure 8

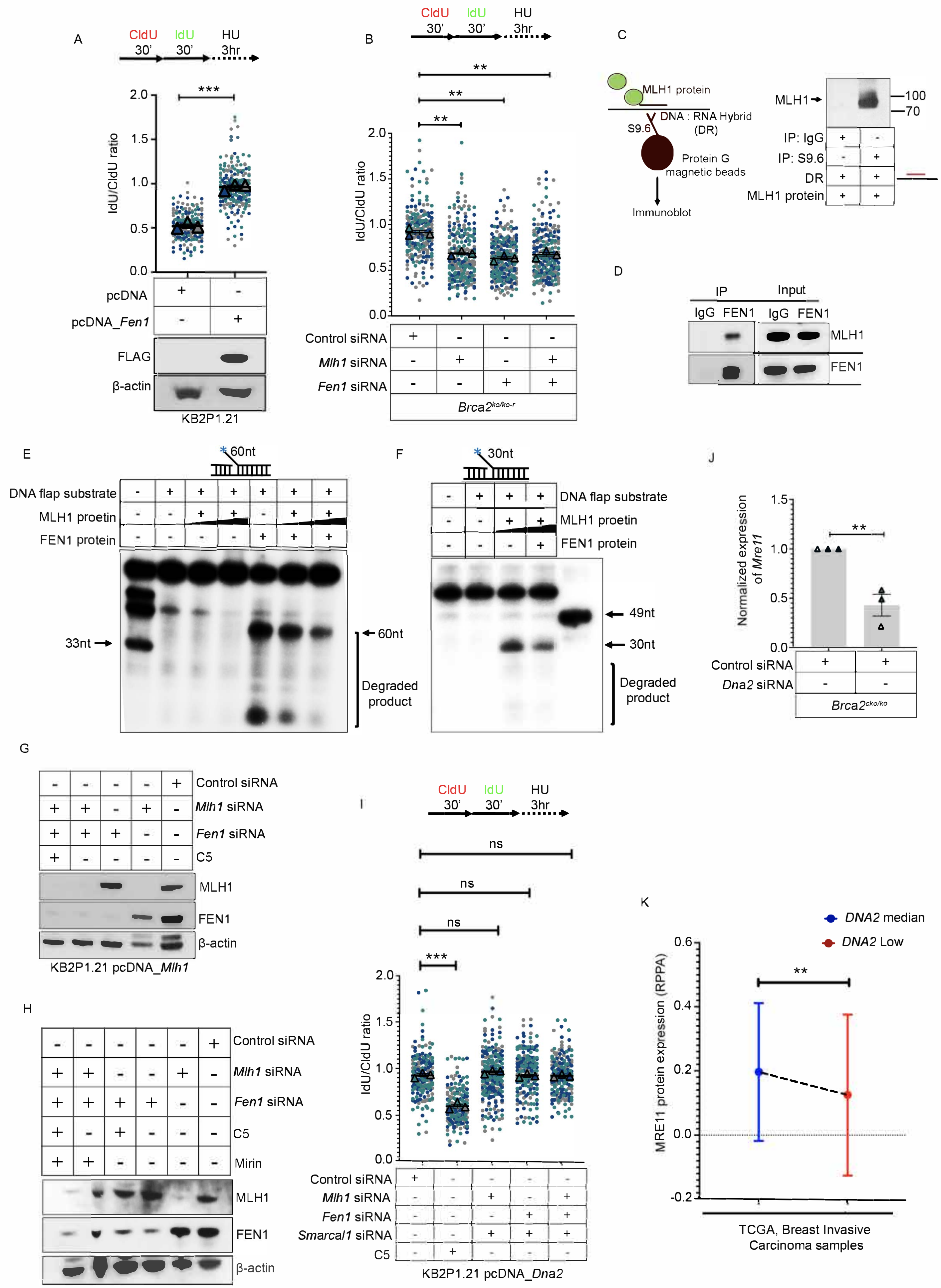

Supplemental Fig. S9.

Increase in transcription but no R-loop accumulation in *Brca2$^{ko/ko-r}$* cells

A) Representative images of immunofluorescence showing the nuclear staining of S9.6 and γH2AX in Control or *Brca2* silenced *Brca2$^{cko/ko}$* mESCs. DAPI was used as a nuclear stain. B) Quantification of immunofluorescence data showing the normalized nuclear intensity of S9.6 and γH2AX in Control or *Brca2* silenced *Brca2$^{cko/ko}$* mESCs. C) Gene Ontology analysis showing the molecular function associated with the genes enriched in *Brca2$^{ko/ko-r}$* clones. Significant enrichments were plotted with respect to *p*-values. Each dot in the graph represents the enrichment in individual *Brca2$^{ko/ko-r}$* clones. D) Gene Ontology analysis showing the biological process associated with the genes enriched in *Brca2$^{ko/ko-r}$* clones. Significant enrichments were plotted with respect to *p*-values. Each dot in the graph represents the enrichment in individual *Brca2$^{ko/ko-r}$* clones. E) ChIP assay showing the normalized enrichment of RNA polymerase II (RNApol2) in gene portion of *Actb* in *Brca2$^{ko/ko-r}$* or *Brca2$^{cko/ko-mi}$* clones by RT-qPCR. IgG was used as a negative control. The values were normalized with respect to corresponding input values. F) DRIP assay showing the enrichment of R-loops in gene portion of *Tet1* in *Brca2$^{ko/ko-r}$* or *Brca2$^{cko/ko-mi}$* clones by RT-qPCR. IgG was used as a negative control and RNaseH treatment was used as a control to confirm the R-loops. The values were expressed as percentage of input. The gene portion of *Tet1* analyzed were depicted (insert). G) RT-qPCR analysis showing the normalized mRNA expression levels of *Rnaseh1* and *Mlh1* in *Brca2$^{ko/ko-r}$* or *Brca2$^{cko/ko-mi}$* clones upon *Mlh1* knockdown. H) DRIP assay showing the enrichment of R-loops in gene portion of *Tet1* in Control or *Mlh1* silenced *Brca2$^{ko/ko-r}$* or *Brca2$^{cko/ko-mi}$* clones by RT-qPCR. Experimental procedure as detailed in (4A) was followed. Insert depicts the gene portion of *Tet1* analyzed. I) DRIP assay showing the enrichment of R-loops in gene portion of *Has2* in Control or *Mlh1* silenced *Brca2$^{ko/ko-r}$* or *Brca2$^{cko/ko-mi}$* clones by RT-qPCR. Experimental procedure as detailed in (4A) was followed. Insert depicts the gene portion of *Has2* analyzed. All the experiments were repeated three times, and the data are presented mean±SEM. Data were analyzed using unpaired, two-tailed student's t-test (B) and unpaired, two-tailed student's t-test with Holm-sidak multiple-comparison test (E-I). *$p<0.05$; **$p<0.01$; ***$p<0.001$.n.s: non-significant.

Supplemental Figure 9

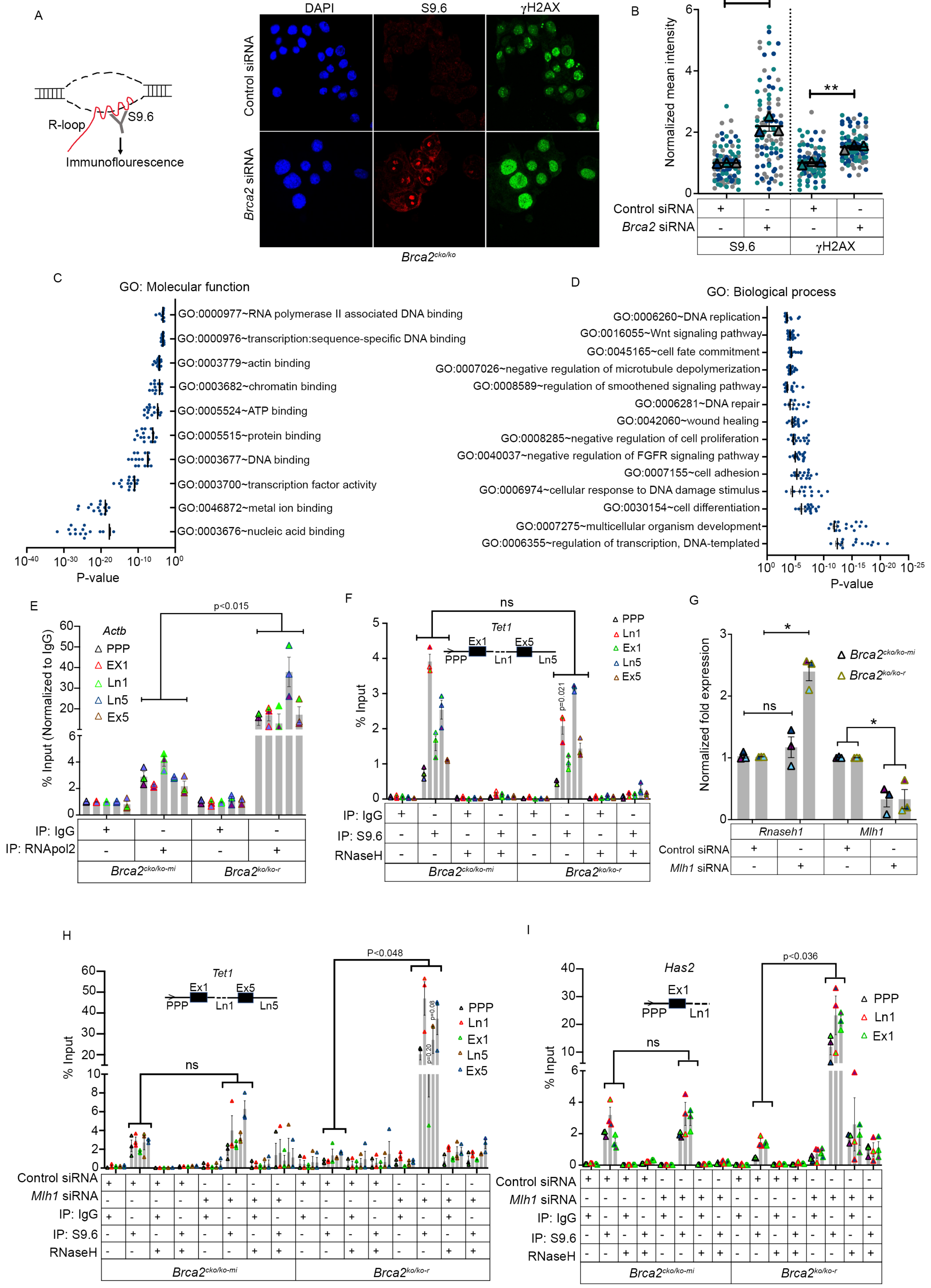

Supplemental Fig. S10.

Induction of R-loops and R-loop associated DNA damage in *Brca2$^{ko/ko-r}$* and camptothecin treated KB2P1.21 upon MLH1 knockdown.

A) Quantification of mean nuclear intensity of S9.6 and γH2AX using ImageJ in Control or *Mlh1* silenced *Brca2$^{ko/ko-r}$* or *Brca2$^{cko/ko-mi}$* clones. Nuclear intensity was measured from ~150 nuclei from three independent experiments. B) Quantification of mean nuclear intensity of S9.6 using ImageJ in Control or *Mlh1* silenced KB2P1.21 cells. Nuclear intensity was measured from ~150 nuclei from three independent experiments. C) Immunofluorescence analysis showing the representative images of S9.6 and γH2AX in Camptothecin treated Control or *Mlh1* or *Mlh3* or combination of *Mlh1/3* silenced KB2P1.21 cells. DAPI was used as a nuclear stain. D) Quantitation of mean nuclear intensity of S9.6 in Camptothecin untreated Control or *Mlh1* silenced KB2P1.21 cells and Camptothecin treated Control or *Mlh1* or *Mlh3* or combination of *Mlh1/3* silenced KB2P1.21 cells. E) Quantitation of γH2AX foci positive and γH2AX foci negative nuclei in Camptothecin treated Control or *Mlh1* or *Mlh3* or combination of *Mlh1/3* silenced KB2P1.21 cells. F) Representative images showing the colony formation assay (9 days) of control or *Mlh1* silenced KB2P1.21 treated with or without Camptothecin (left panel). Quantitation of crystal violet intensity in Control silenced or *Mlh1* silenced KB2P1.21 treated with or without Camptothecin (right panel). G) Quantitation of percentage cell viability in *Rnaseh1* over expressing Control silenced or *Mlh1* silenced KB2P1.21 treated with or without camptothecin. All experiments were repeated three times and data are presented as mean±SEM. Data were analyzed using unpaired, two-tailed student's t-test (A, B, D, F) and unpaired, two-tailed student's t-test with Holm-sidak multiple-comparison test (E). *$p<0.05$; **$p<0.01$; ***$p<0.001$; ****$p<0.0001$. n.s: non-significant.

Supplemental Figure 10

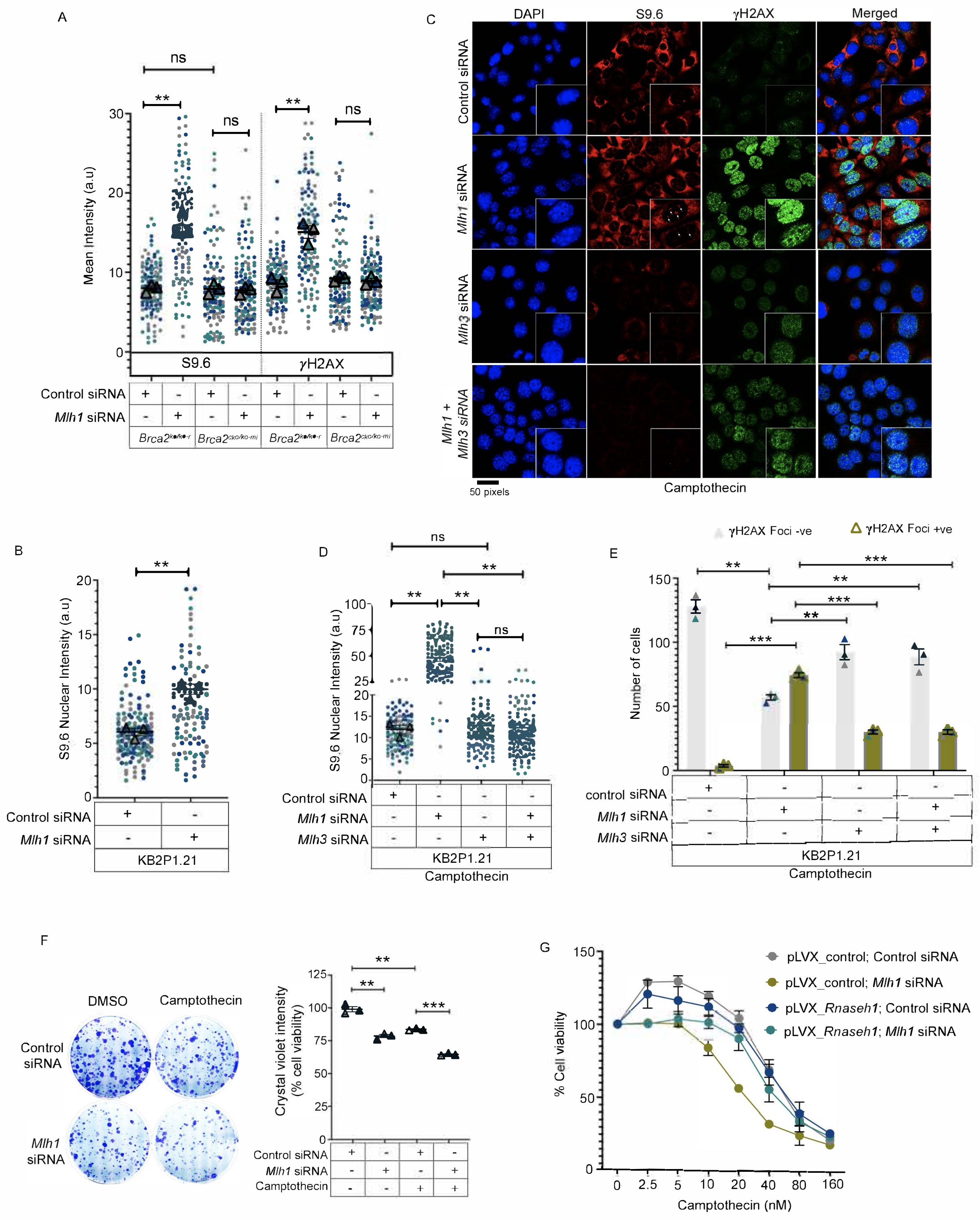

Supplemental Fig. S11.

MLH1 over-expression reduces genomic instability in *BRCA2*^{Y3308X} whereas its loss induces genomic instability in *Brca2*$^{ko/ko\text{-}r}$ cells.

A) Quantitation of chromosomal aberrations in DMSO or Olaparib (100nM) or camptothecin (10nM) or MMC (20ng/μl) or cisplatin (0.6uM) treated *Brca2*$^{ko/ko\text{-}r}$ clones. Aberrations per nuclei were plotted. B) Immunofluorescence analysis showing the representative images of γH2AX foci in Control or *Mlh1* silenced *Brca2*$^{ko/ko\text{-}r}$ and *Brca2*$^{cko/ko\text{-}mi}$ clones. DAPI was used as a nuclear stain. Experiments were repeated three times and representative image from one independent experiment is shown. C) Quantitation of chromosomal aberrations in hydroxyurea treated and/or untreated Control or *Mlh1* silenced *Brca2*$^{ko/ko\text{-}r}$ clones. Aberrations per nuclei were plotted. D) Quantitation of chromosomal aberrations in Hydroxyurea treated and /or untreated Control or *MLH1* silenced U2OS cells. Aberrations per nuclei were plotted. E) WGS analysis showing a total of 185 unique SVs for Y3308X clone, and 29/23 unique SVs for *Mlh1* over expressing *BRCA2*^{Y3308X} clone (pLVX_*Mlh1* #1), which were both predominantly by deletions. The biological repeats pLVX_*Mlh1* #2 also showed similar levels and *BRCA2*^{Y3308X} clone had increased SVs when compared to MLH1 over expressed. F) Quantitation of mutation density observed in *Brca2*$^{cko/ko\text{-}mi}$ (n=2; marked by red arrow) and *Brca2*$^{ko/ko\text{-}r}$ (n=18; unmarked). All experiments were repeated three times (A, B, C, D). Experiments from WGS (E, F) were performed only once with multiple clones. Data are presented as mean±SEM for (A, C, D). Data were analyzed using unpaired, two-tailed student's t-test (A, C, D). *p<0.05; **p<0.01; ***p<0.001; ****p<0.0001. n.s: non-significant.

Supplemental Figure 11

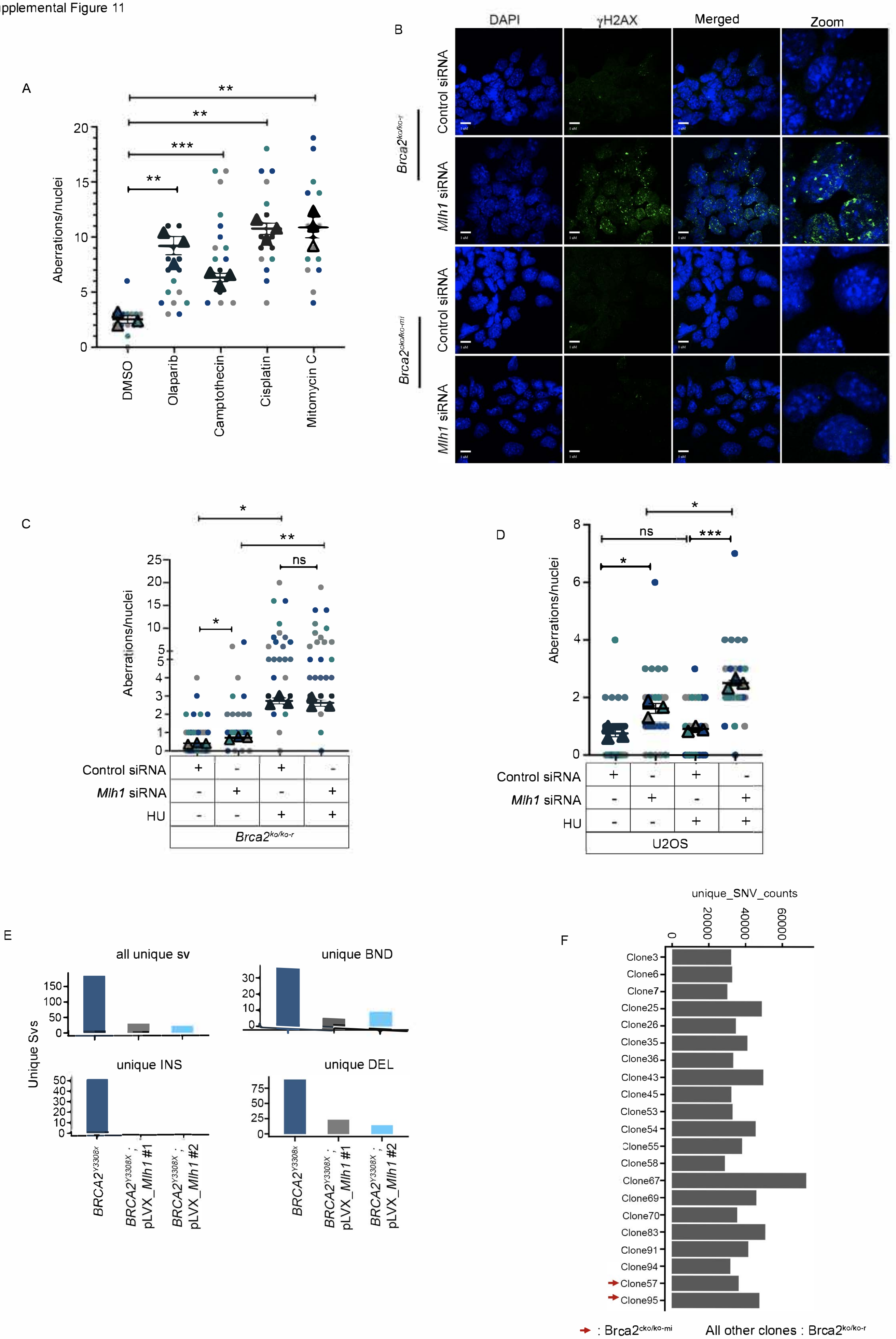

Supplemental Fig. S12.

Low expression of MLH1 induces genome alterations and DNA damage response in patient samples.

A) TCGA dataset analysis (TCGA, Cell) showing the percentage genome altered in *BRCA2* Low *MLH1* median or *MLH1* low or *MLH1* high breast cancer patient samples. B) TCGA dataset analysis (TCGA, Cell) showing the phosphorylation status of CHEK1 and CHEK2 in *BRCA2* Low; *MLH1* median or *MLH1* low or *MLH1* high breast cancer patient samples. The levels of total CHEK1 and CHEK2 acts as a control. C) TCGA dataset analysis (TCGA, Firehose Legacy) showing the percentage genome altered in *BRCA2* High; *MLH1* median or *MLH1* low or *MLH1* high breast cancer patient samples. D) TCGA dataset analysis (TCGA, Cell) showing the percentage genome altered in *BRCA2* High; *MLH1* median or *MLH1* low or *MLH1* high breast cancer patient samples. E) TCGA dataset analysis (TCGA, Firehose Legacy) showing the phosphorylation status of CHEK1 and CHEK2 in *BRCA2* high; *MLH1* median or *MLH1* low or *MLH1* high breast cancer patient samples. The levels of total CHEK1 and CHEK2 acts as a control. F) TCGA dataset analysis (TCGA, Cell) showing the phosphorylation status of CHEK1 and CHEK2 in *BRCA2* high; *MLH1* median or *MLH1* low or *MLH1* high breast cancer patient samples. The levels of total CHEK1 and CHEK2 acts as a control. G) TCGA dataset analysis (TCGA, Firehose Legacy) showing the percentage genome altered in *BRCA2* High; *MLH3* median or *MLH3* low or *MLH3* high breast cancer patient samples. H) TCGA dataset analysis (TCGA, Firehose Legacy) showing the phosphorylation status of CHEK1 and CHEK2 in *BRCA2* Low; *MLH3* median or *MLH3* low or *MLH3* high breast cancer patient samples. The levels of total CHEK1 and CHEK2 acts as a control. I) TCGA dataset analysis (TCGA, Cell) showing the phosphorylation status of CHEK1 and CHEK2 in *BRCA2* Low; *MLH3* median or *MLH3* low or

MLH3 high breast cancer patient samples. The levels of total CHEK1 and CHEK2 acts as a control. Note: TCGA breast dataset (TCGA Cell and Firehose Legacy) have some over-lapping samples. Sample overlaps were observed between (E) and (F) ; (H) and (I). Data are presented as mean±s.d for (A-I). cBioportal Query used: (BRCA2 Low-BRCA2: exp<-0.75; BRCA2 High-BRCA2: exp>0.75; MLH1 Low-MLH1: exp<-0.5 and MLH1 High-MLH1: exp>0.5). Data were analyzed using Wilcoxon's rank sum test (A, C, D, G) and unpaired, two-tailed student's t-test (B, E, F, H, I). *p<0.05; **p<0.01; ***p<0.001; ****p<0.0001. n.s: non-significant.

Supplemental Figure 12

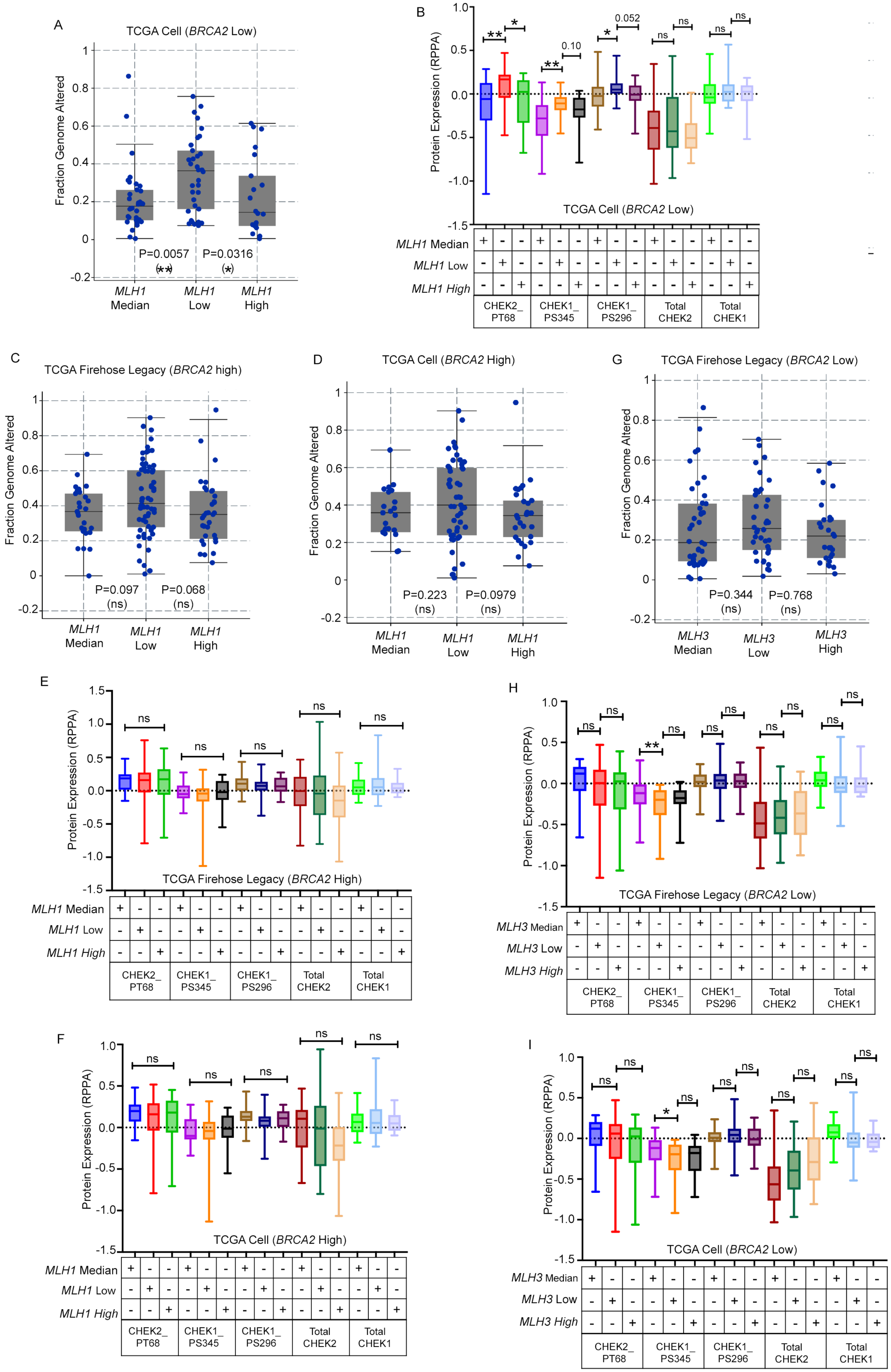

Supplemental Fig. S13.

***ESR1* positively correlated with *MLH1* expression in breast cancer patients and MLH1 is downstream of ERα signaling.**

A) Box plot comparing the expression analysis of *MLH1* between tumor vs normal samples in TCGA- BRCA dataset with matched TCGA normal and GTEx data (p-value= 0.01). B) Box plot showing the expression analysis of *MLH1* transcript among various breast cancer subtypes in TCGA samples; *MLH1* RNA expression is predominantly higher in luminal subtype. C) Jitter plot showing the expression analysis of MLH1 protein among various breast cancer subtypes accessed from Clinical Proteomic Tumor Analysis Consortium (CPTAC); MLH1 protein expression is predominantly higher in luminal subtype. Z-values represent standard deviations from the median across samples. D) Scatter plot showing the positive correlation between *ESR1* and *MLH1* gene expression in TCGA-BRCA tumor dataset (p-value= 0). E) Scatter plot analysis via UALCAN tool showing *ESR1-MLH1* (mRNA) positive correlation among tumor samples (Pearson Correlation coefficient: 0.4). F) Colored bar graphs showing the gene expression comparison between *ESR1* and *MLH1* between tumors vs normal samples suggesting a probable positive correlation between them. Analysis included the matched TCGA normal and GTEx data. BRCA represents breast cancer. G) TCGA dataset analysis (TCGA, Firehose Legacy) showing the percentage of samples expressing low (expression<-0.5) or high (expression>0.5) *ESR1* and *MLH1* in ER negative, ER positive and Triple negative breast cancer patient samples. H) TCGA analysis showing the co-occurrence of *MLH1* and *ESR1* in breast cancer patient samples. I) ERE consensus sequence (GGTCAnnnTGACC) retrieved from Jaspar database. J) Presence of ERE (Estrogen Receptor Elements) within the *MLH1* promoter region spanning from -2kb to +100bp. Cartoon diagram (showing the ER alpha binding sites), is generated from the information retrieved from

the EPD database with p-value set at 0.001. Orange EREs shows moderate whereas red ERE shows maximum similarity (0.826046) similarity between the genomic regions and the known ESR1 consensus sequence (0.1 being least and 1 being max similarity). EPD, TFBIND and JASPAR are used for datamining.

Supplemental Figure 13

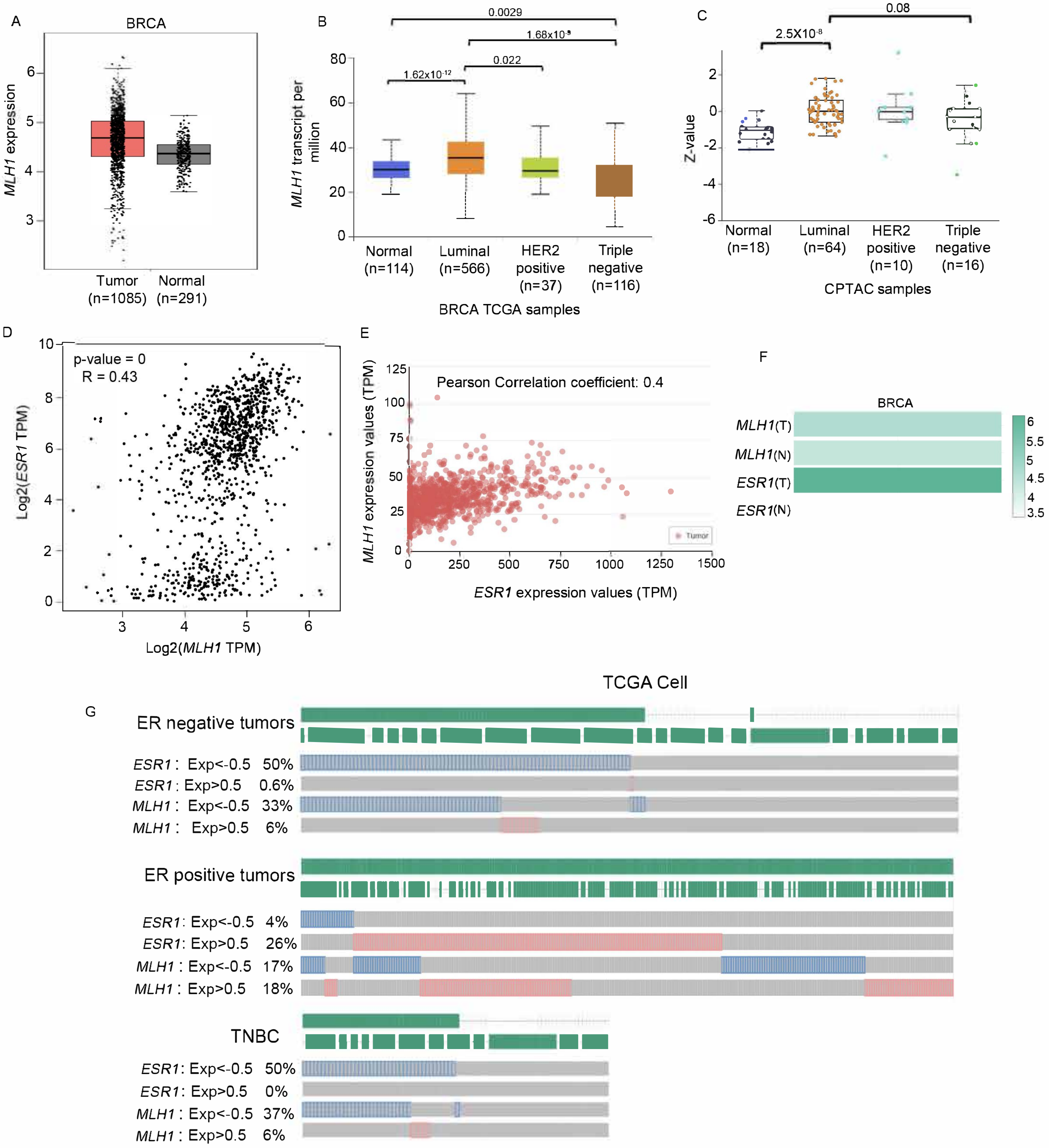

H

A	B	Neither	A Not B	B Not A	Both	Log2 Odds Ratio	p-value	q-value	Tendency
MLH1	*ESR1*	66	4	2	2	>3	0.031	0.031	Co-occurece

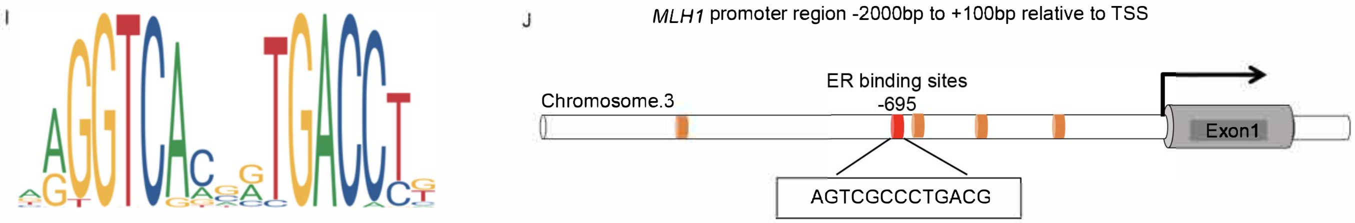

Supplemental Fig. S14.

MLH1 expression in different PDX models.

A) MLH1 mRNA expression across PDX models, ER/PR/HER2 and BRCA1/2 mutation status as retrieved from the BCM PDX portal (see Methods).

Supplemental Figure 14

A

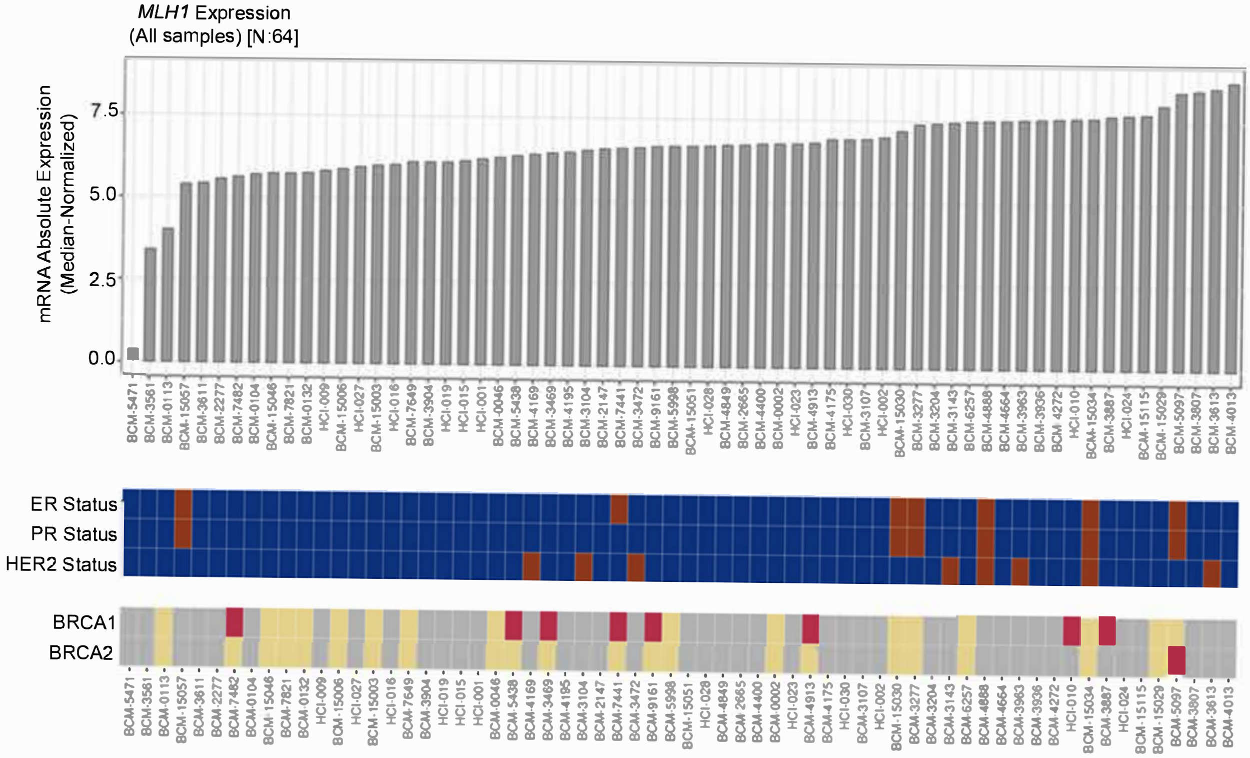

Tables S1

Table S1: Primers used in the study.

**Oligos used for RT-qPCR**	
**Primers**	**5'-Sequence-3'**
Mlh1 Forward	TTGGCACAGCACCAGACCAA
Mlh1 Reverse	ACTGTCTAAGGCCAGCATGG
Msh2 Forward	GGGATTCACGTGGCTGAGCT
Msh2 Reverse	CCAGGCAGCGTCTCTTTGCA
Mlh3 Forward	CATGTGTGGCCATCAGAGTG
Mlh3 Reverse	TGCTGGGTTCTCCAAGTCCC
Rnaseh1 Forward	GCCATCATGCAAGCCAAG
Rnaseh1 Reverse	GTCCAGCTCCATGAAGTC
Gapdh Forward	TGCCCCCATGTTTGTGATG
Gapdh Reverse	TGTGGTCATGAGCCCTTCC
Dna2 Forward	TGCAGCTGGCGCACTTGA
Dna2 Reverse	GCAGTTGCCACTCTCCTC
Mre11 Forward	AGTGCTGAGCGGGAACGA
Mre11 Reverse	TTTTGTTCCCTGTGCCTG
**Oligos used for DRIP and ChIP**	
**Primers**	**5'-Sequence-3'**
Actb PPP Forward	ATATGCTGGGGTGATGGTCA
Actb PPP Reverse	GCATAGGTAGGTGTTCACTG
Actb Ln1 Forward	ATCCGGCCCTCAACCCTGAC
Actb Ln1 Reverse	TGAGTTGTGCACTAGTCCAT
Actb Ex1 Forward	ATGCACTGAGGCACGGCAGG
Actb Ex1 Reverse	GCCAGCTATTTGGCTGAGAG
Actb Ln5 Forward	CAGTGCTGTGGGTTTAGG
Actb Ln5 Reverse	GACTGTGCTGGGAGTCTC
Actb Ex5 Forward	CCGGGACCTGACAGACTA

Actb Ex5 Reverse	GGATGCGGCAGTGGCCAT
Tet1 Ex1 Forward	ATGTCTCGGTCCCGCCCC
Tet1 Ex1 Reverse	CCGGGGTTCACTGCCTTC
Tet1 PPP Forward	CACTGAAAGAGATGGTGA
Tet1 PPP Reverse	TCGGAGCCGGACTCCTCT
Tet1 Ln1Forward	GTCACATGCCACTAAGCC
Tet1 Ln1 Reverse	GACAGGCATGTTGGCTTG
Tet1 Ex5 Forward	CAGATGGAGGTACACAAA
Tet1 Ex5 Reverse	GGGAAGCAGAGTTCCTCA
Tet1 Ln5 Forward	GCGTCAGATCCGCTTACA
Tet1 Ln5 Reverse	GGGAGTGGACACATTAGA
Has2 PPP Forward	TGAATGGCCAATTTCTCT
Has2 PPP Reverse	GTGAGCCGGTGGGTGTTT
Has2 Ln1Forward	ATTGTGTGCACTCGCTCT
Has2 Ln1 Reverse	GCCTGGAGGAAGACTGCA
Has2 Ex1 Forward	ATGCATTGTGAGAGGTTT
Has2 Ex1 Reverse	CCGTACAGTCCAAATGAG
**Oligos used for *in vitro* binding studies (5'-Sequence-3')**	
DNA oligo #1	5'GCCAGGGACGAGGTGAACCTGCAGGTGGGCGGCTACTACTTAGATGTC ATCCGAGGCTTATTGGTAGAATTCGGCAGCGTCATGCGACGGC3'
DNA oligo #2	5'GCCGTCGCATGACGCTGCCGAATTCTACCACGCGATTCATACCTGTCGT GCCAGCTGCTTTGCCCACCTGCAGGTTCACCTCGTCCCTGGC3'
RNA oligo	5'GCAGCUGGCACGACAGGUAUGAAUC3'
RNA oligo with tail	5'CUGGAUCUAAGCAUCAAGUAGCAGCUGGCACGACAGGUAUGAAUC3'
**Oligos used for genotyping (5'-Sequence-3')**	
Mlh1 Forward 1	TGTCAATAGGCTGCCCTAGG
Mlh1 Reverse 2	TGGAAGGATTGGAGCTACGG
Mlh1 Reverse 3	TTTTCAGTGCAGCCTATGCTC
Brca2 L2431P Forward	TTAGCCTCACAGCCTCTCCTCGT

Brca2 L2431P Reverse	AGAGGGCCCTATCAGGTGTT

References

1. Ding X, Chaudhuri AR, Callen E, Pang Y, Biswas K, Klarmann KD, et al. Synthetic viability by BRCA2 and PARP1/ARTD1 deficiencies. *Nature Communications.* 2016;7(1):12425.
2. Kharat SS, Ding X, Swaminathan D, Suresh A, Singh M, Sengodan SK, et al. Degradation of 5hmC-marked stalled replication forks by APE1 causes genomic instability. *Sci Signal.* 2020;13(645).
3. Sengodan SK, Nadhan R, Nair RS, Hemalatha SK, Somasundaram V, Sushama RR, et al. BRCA1 regulation on β-hCG: a mechanism for tumorigenicity in BRCA1 defective breast cancer. *Oncogenesis.* 2017;6(9):e376.
4. Gu Z, Gu L, Eils R, Schlesner M, and Brors B. circlize Implements and enhances circular visualization in R. *Bioinformatics.* 2014;30(19):2811-2.
5. Ernst J, and Kellis M. ChromHMM: automating chromatin-state discovery and characterization. *Nat Methods.* 2012;9(3):215-6.
6. Singh VK, Rastogi A, Hu X, Wang Y, and De S. Mutational signature SBS8 predominantly arises due to late replication errors in cancer. *Commun Biol.* 2020;3(1):421.
7. Rosenthal R, McGranahan N, Herrero J, Taylor BS, and Swanton C. DeconstructSigs: delineating mutational processes in single tumors distinguishes DNA repair deficiencies and patterns of carcinoma evolution. *Genome Biol.* 2016;17:31.
8. Mishra AP, Hartford SA, Sahu S, Klarmann K, Chittela RK, Biswas K, et al. BRCA2-DSS1 interaction is dispensable for RAD51 recruitment at replication-induced and meiotic DNA double strand breaks. *Nat Commun.* 2022;13(1):1751.
9. Zhang X, Claerhout S, Prat A, Dobrolecki LE, Petrovic I, Lai Q, et al. A renewable tissue resource of phenotypically stable, biologically and ethnically diverse, patient-derived human breast cancer xenograft models. *Cancer Res.* 2013;73(15):4885-97.
10. Balamurugan K, Poria DK, Sehareen SW, Krishnamurthy S, Tang W, McKennett L, et al. Stabilization of E-cadherin adhesions by COX-2/GSK3β signaling is a targetable pathway in metastatic breast cancer. *JCI Insight.* 2023;8(6).
11. Sachs N, de Ligt J, Kopper O, Gogola E, Bounova G, Weeber F, et al. A Living Biobank of Breast Cancer Organoids Captures Disease Heterogeneity. *Cell.* 2018;172(1-2):373-86.e10.
12. Sharan SK, Morimatsu M, Albrecht U, Lim D-S, Regel E, Dinh C, et al. Embryonic lethality and radiation hypersensitivity mediated by Rad51 in mice lacking Brca2. *Nature.* 1997;386(6627):804-10.
