# Supplementary material for: Mismatch repair protein MLH1 suppresses replicative stress in BRCA2-deficient breast tumors
Source: J Clin Invest. 2024 Jan 25;134(7):e173718. doi: 10.1172/JCI173718 (PMC10977984; doi:10.1172/JCI173718)

Full unedited blot Figure 1F

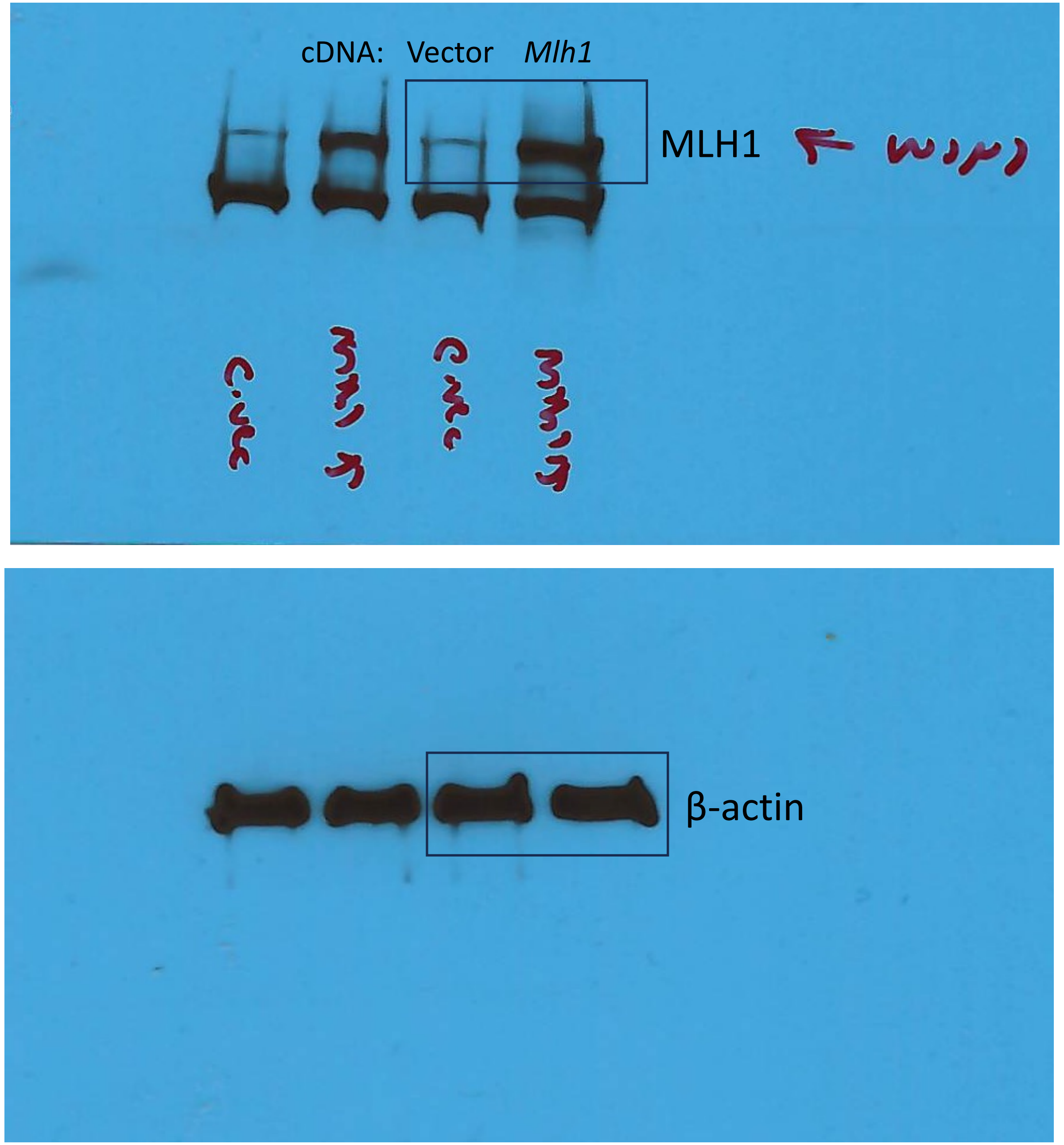

Full unedited blot Figure 1G

cDNA: Vector

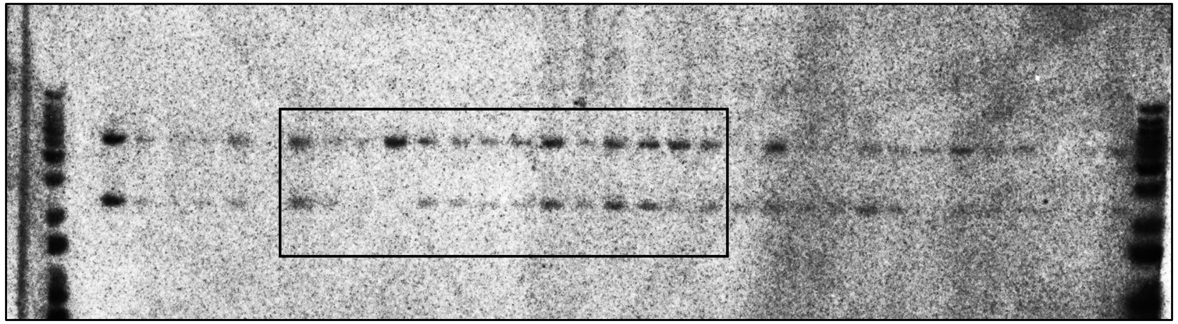

cDNA: *Mlh1*

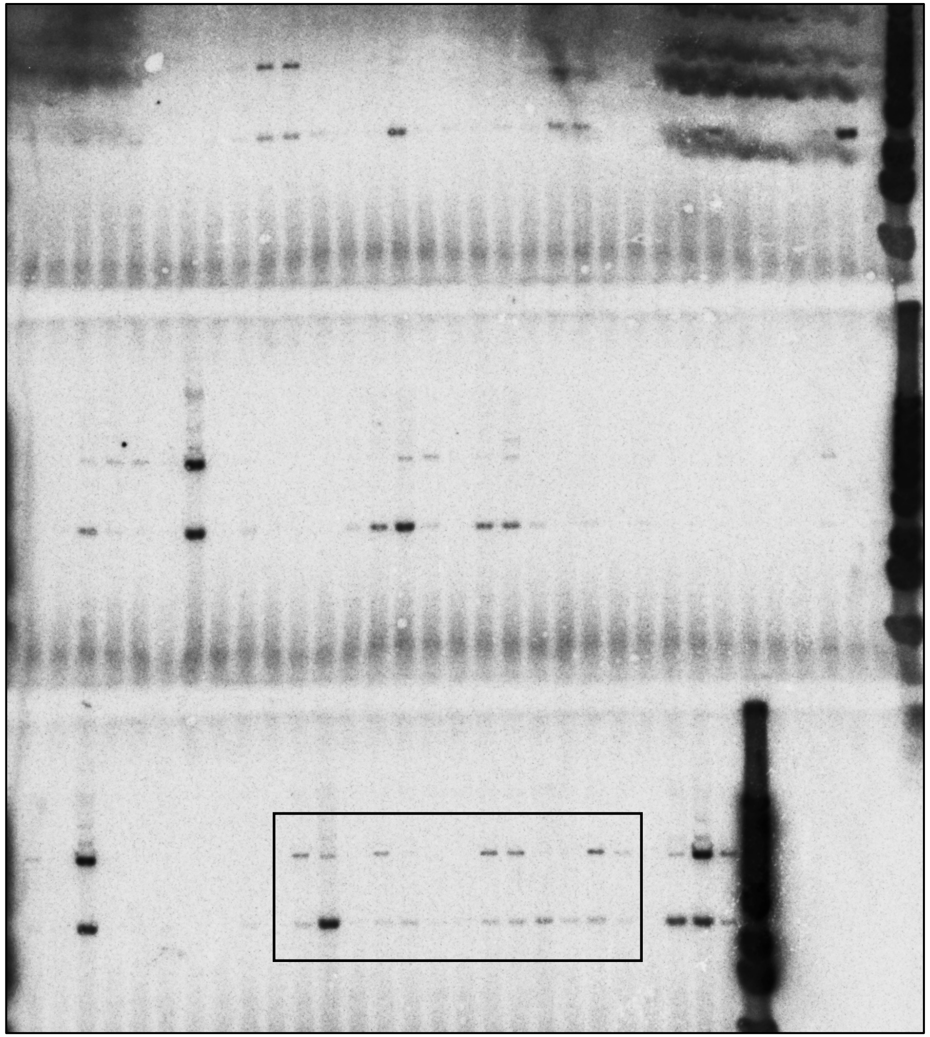

Full unedited blot for Supplemental Figure 1A

DMSO

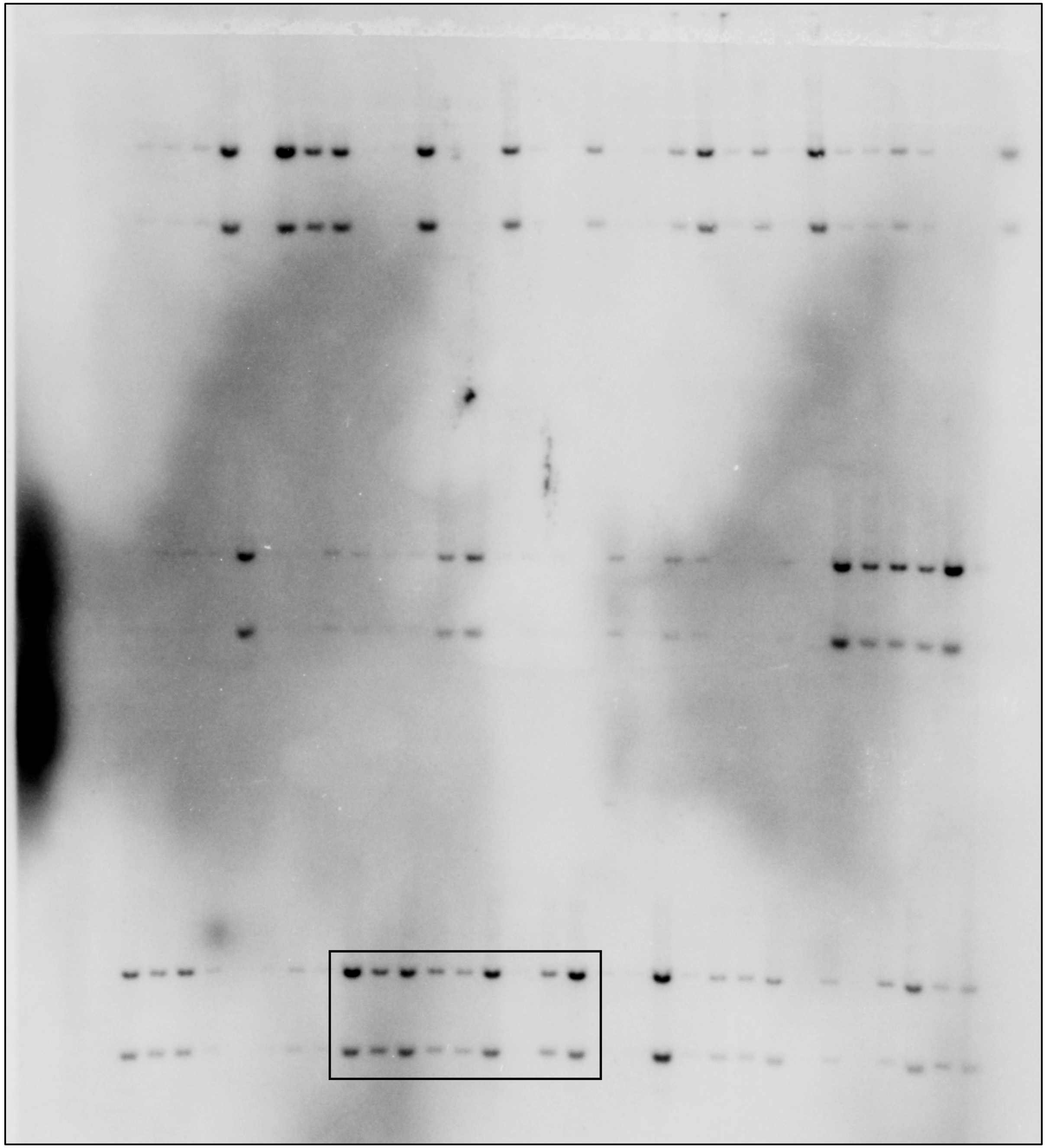

Full unedited blot for Supplemental Figure 1A

Mirin#1

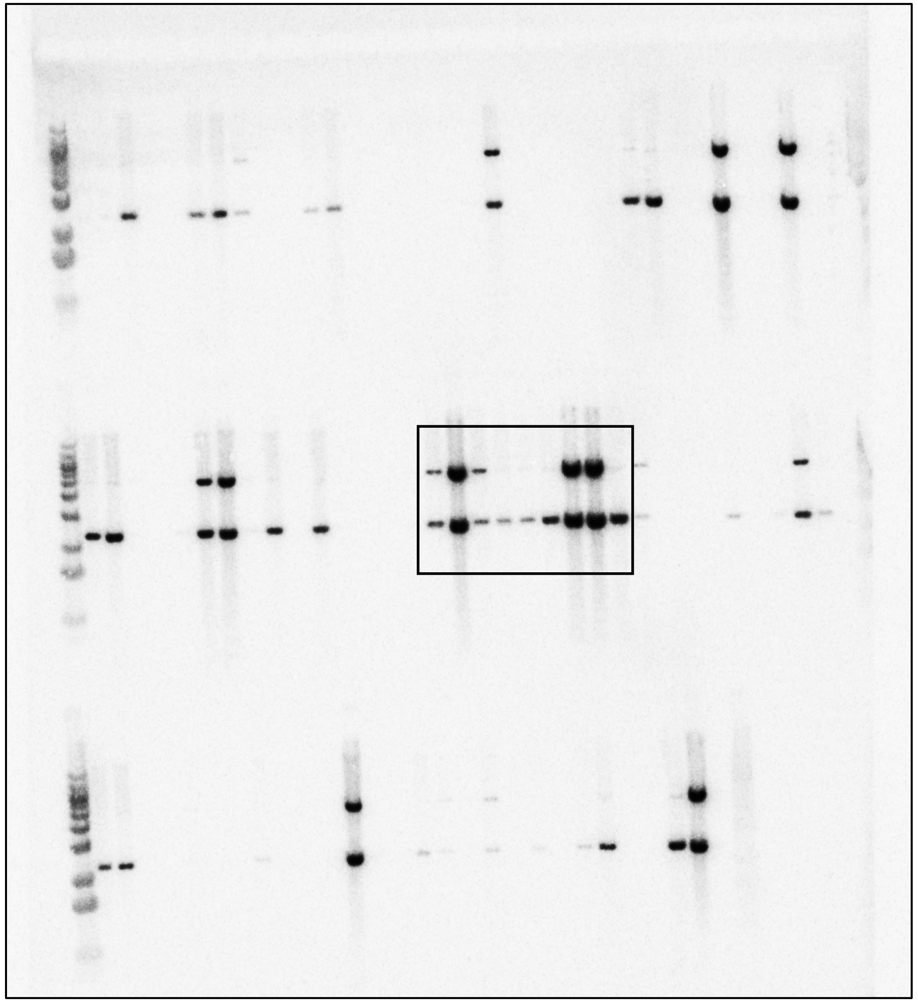

Mirin#2

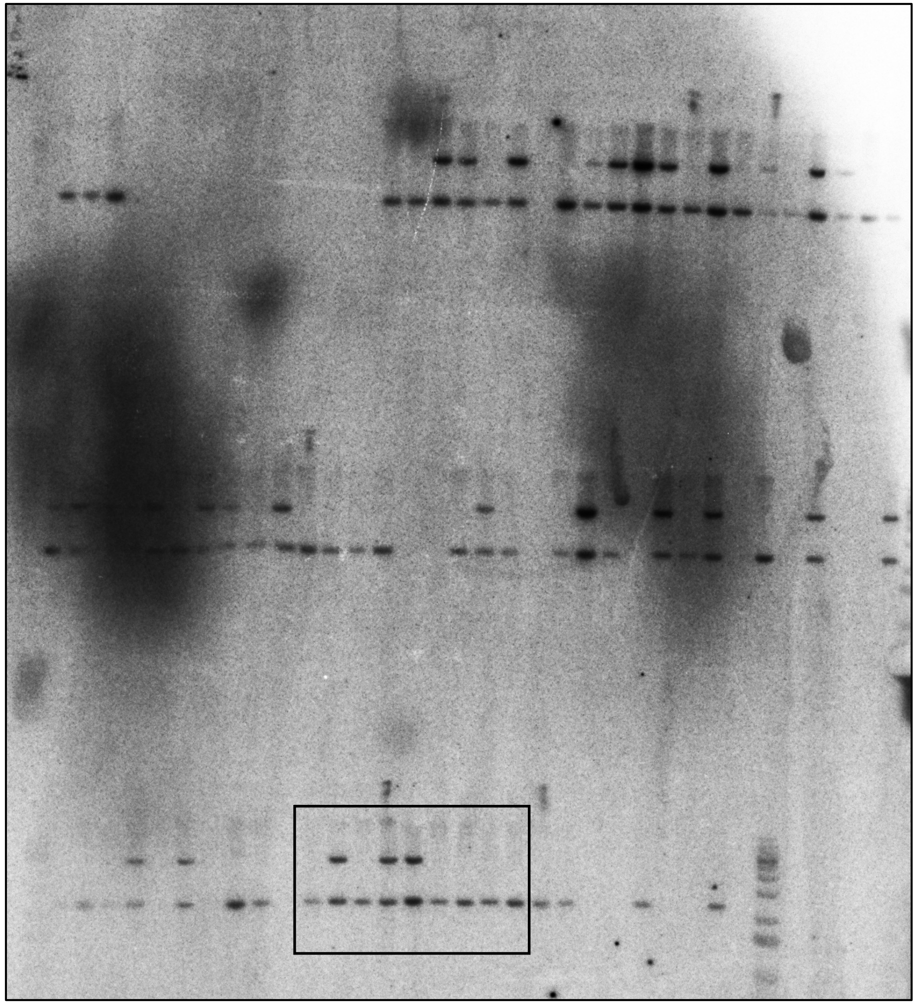

Full unedited blot for Supplemental Figure 1D

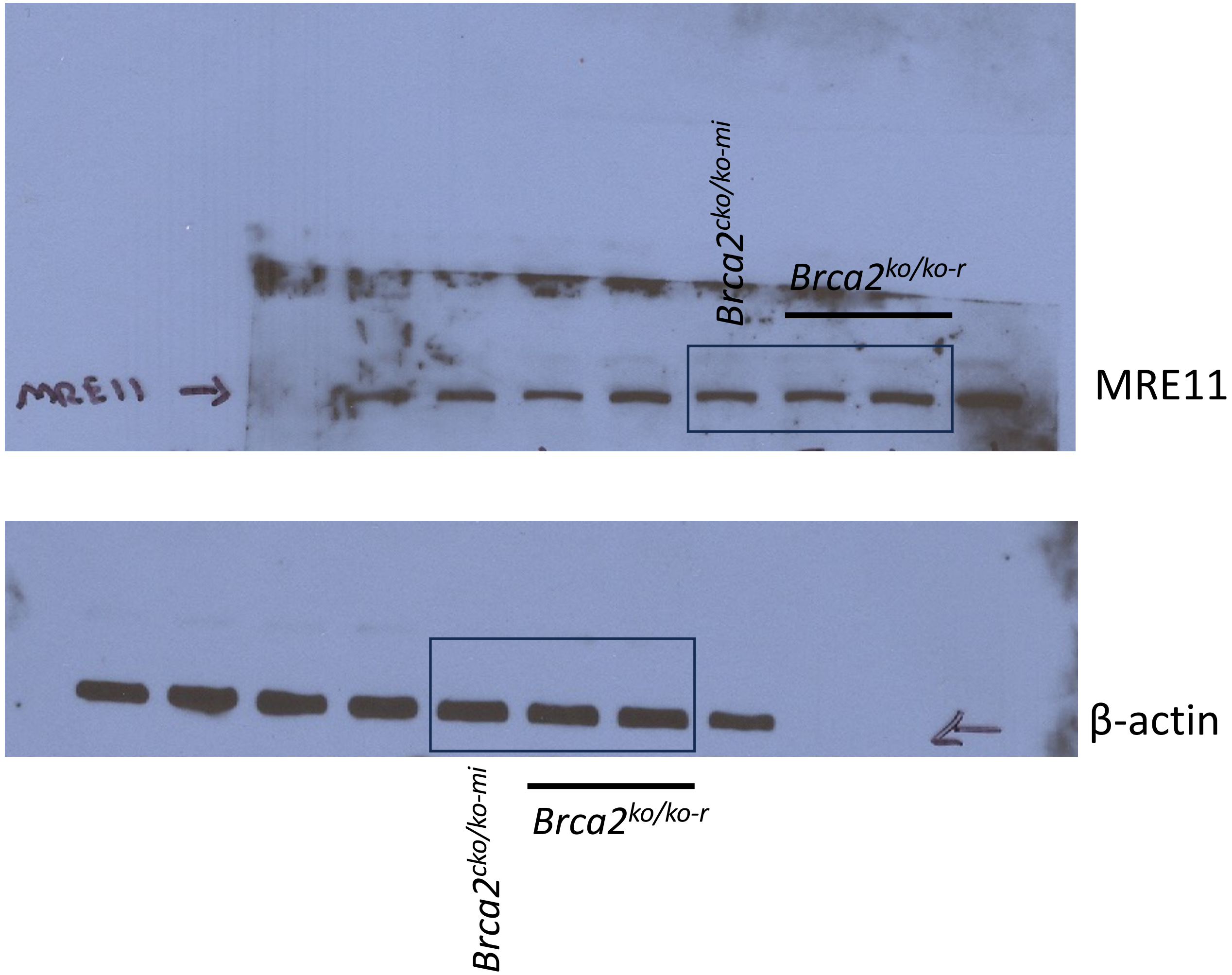

Full unedited blot for Supplemental Figure 1E

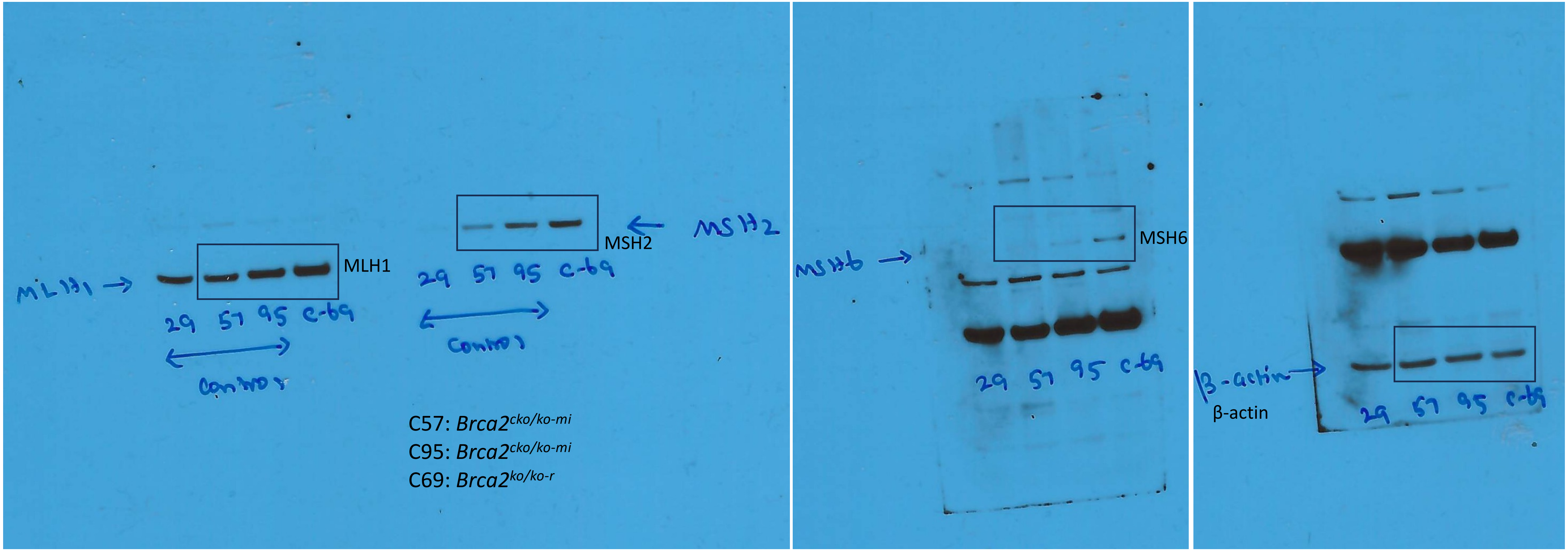

Full unedited blot for Supplemental Figure 2A

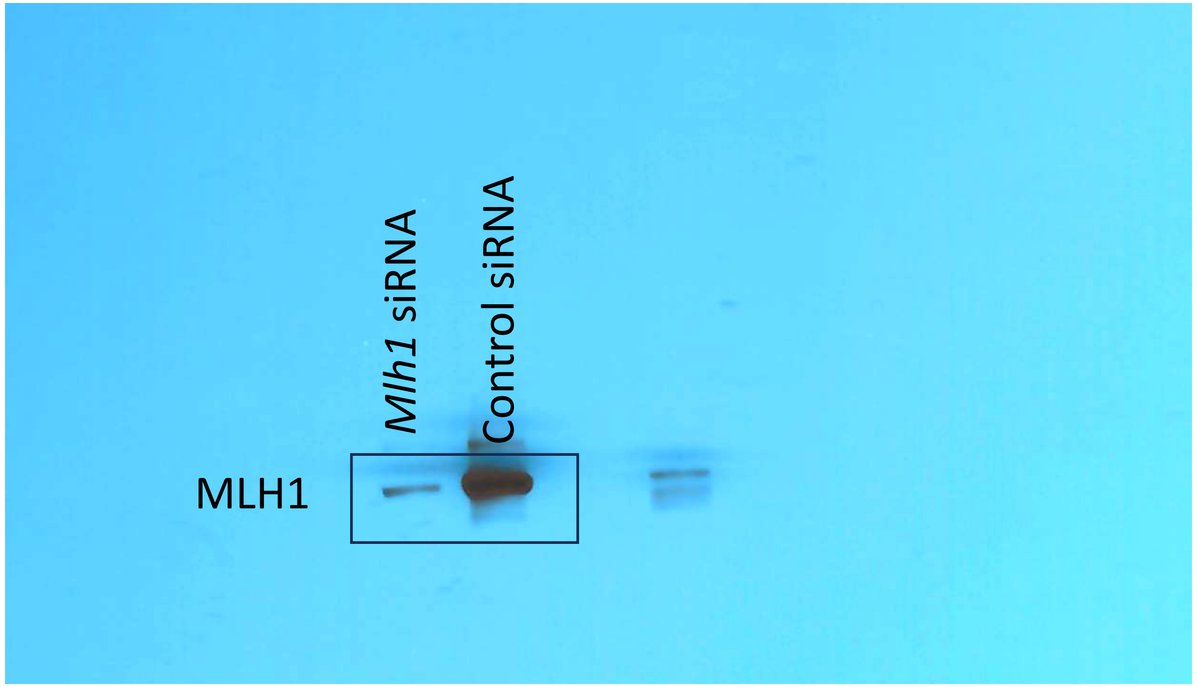

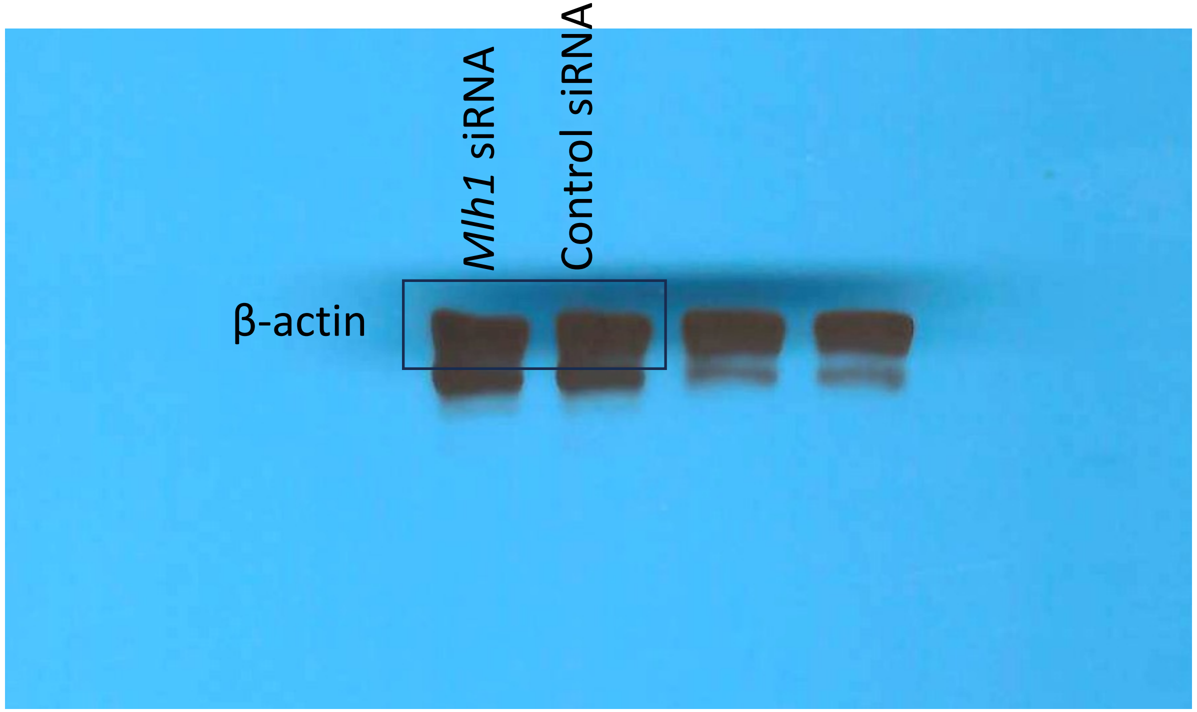

Full unedited blot for Supplemental Figure 2D

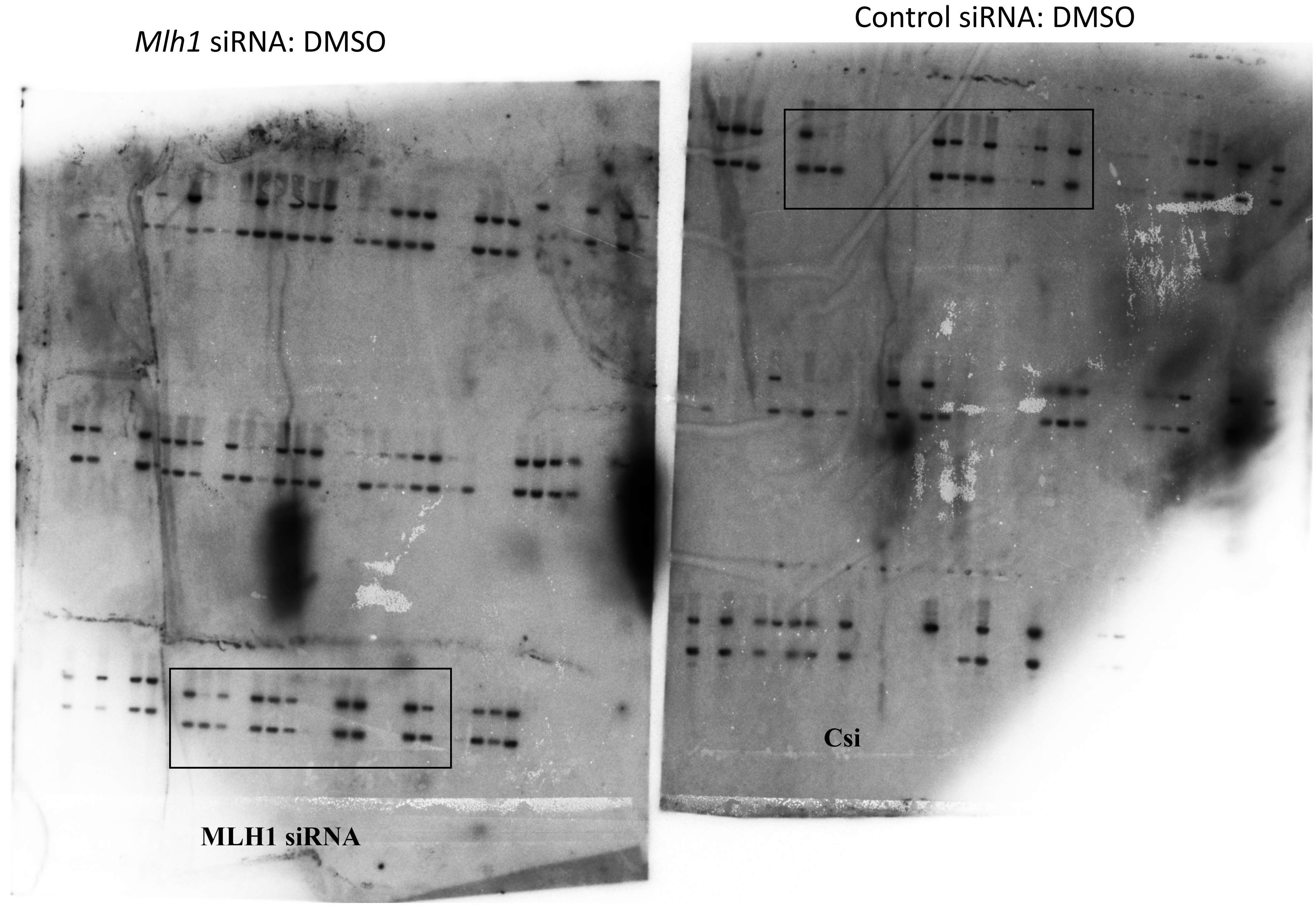

Full unedited blot for Supplemental Figure 2D

Mlh1 siRNA: Olaparib

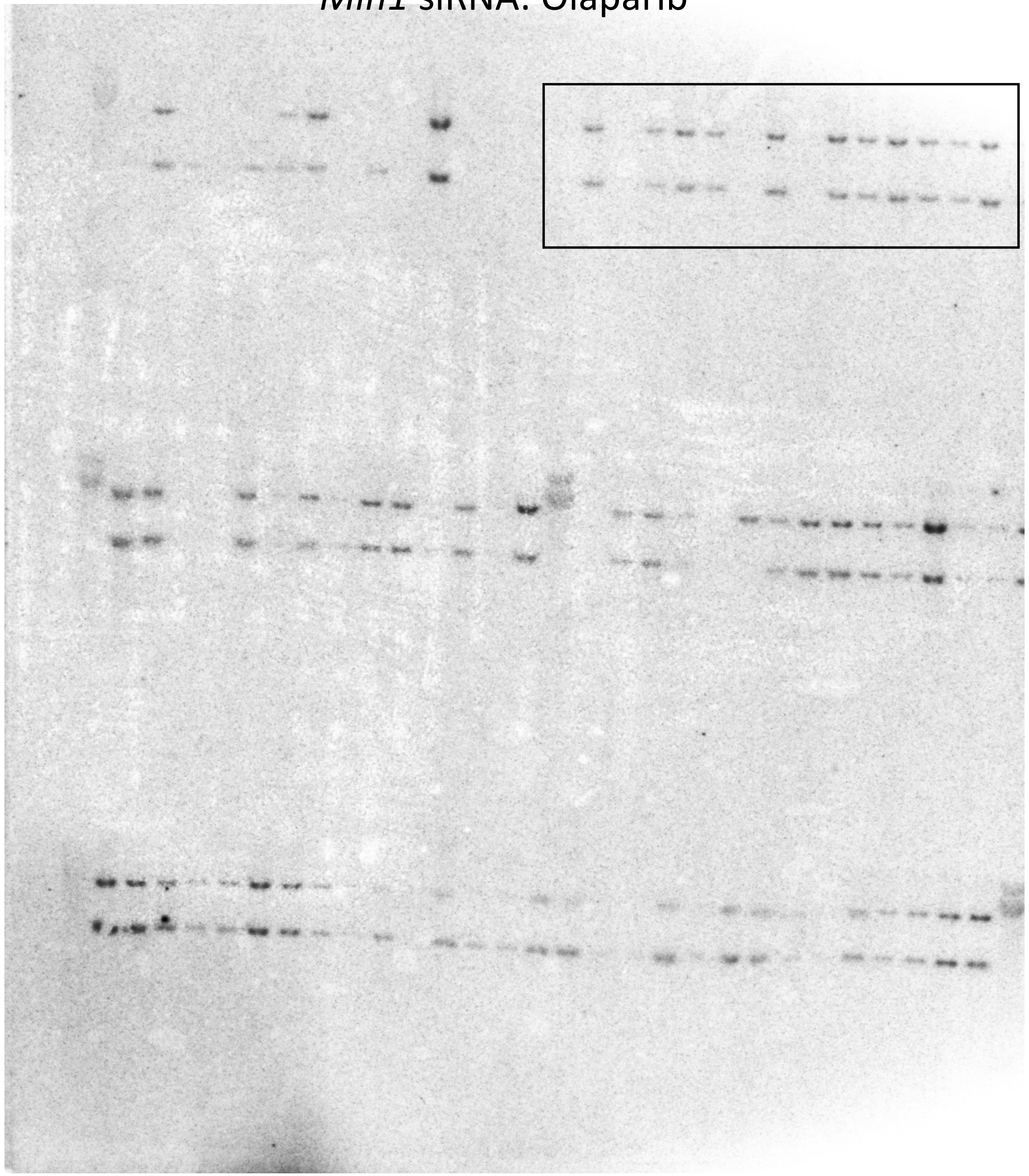

Control siRNA: Olaparib

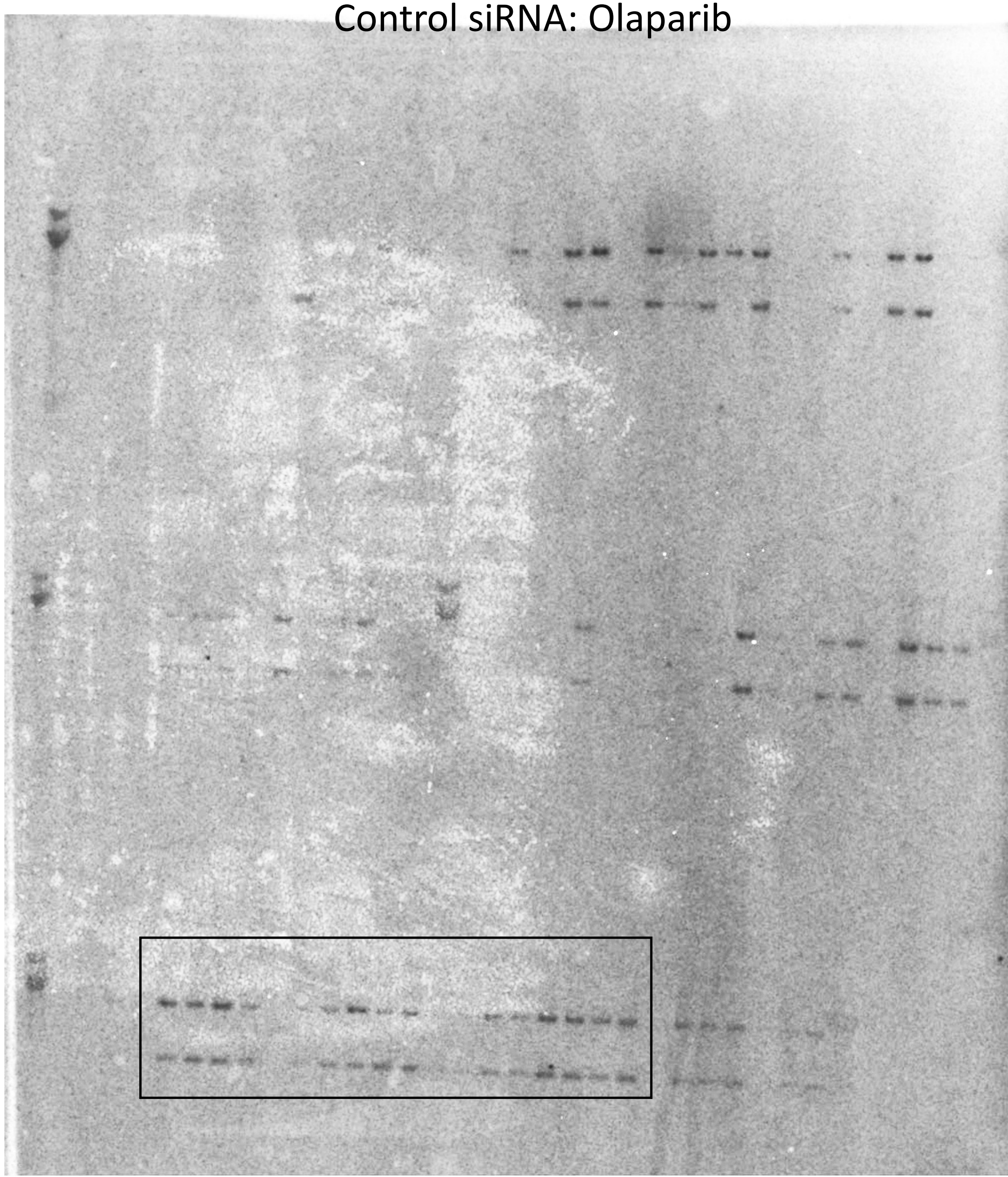

Full unedited blot for Supplemental Figure 2E

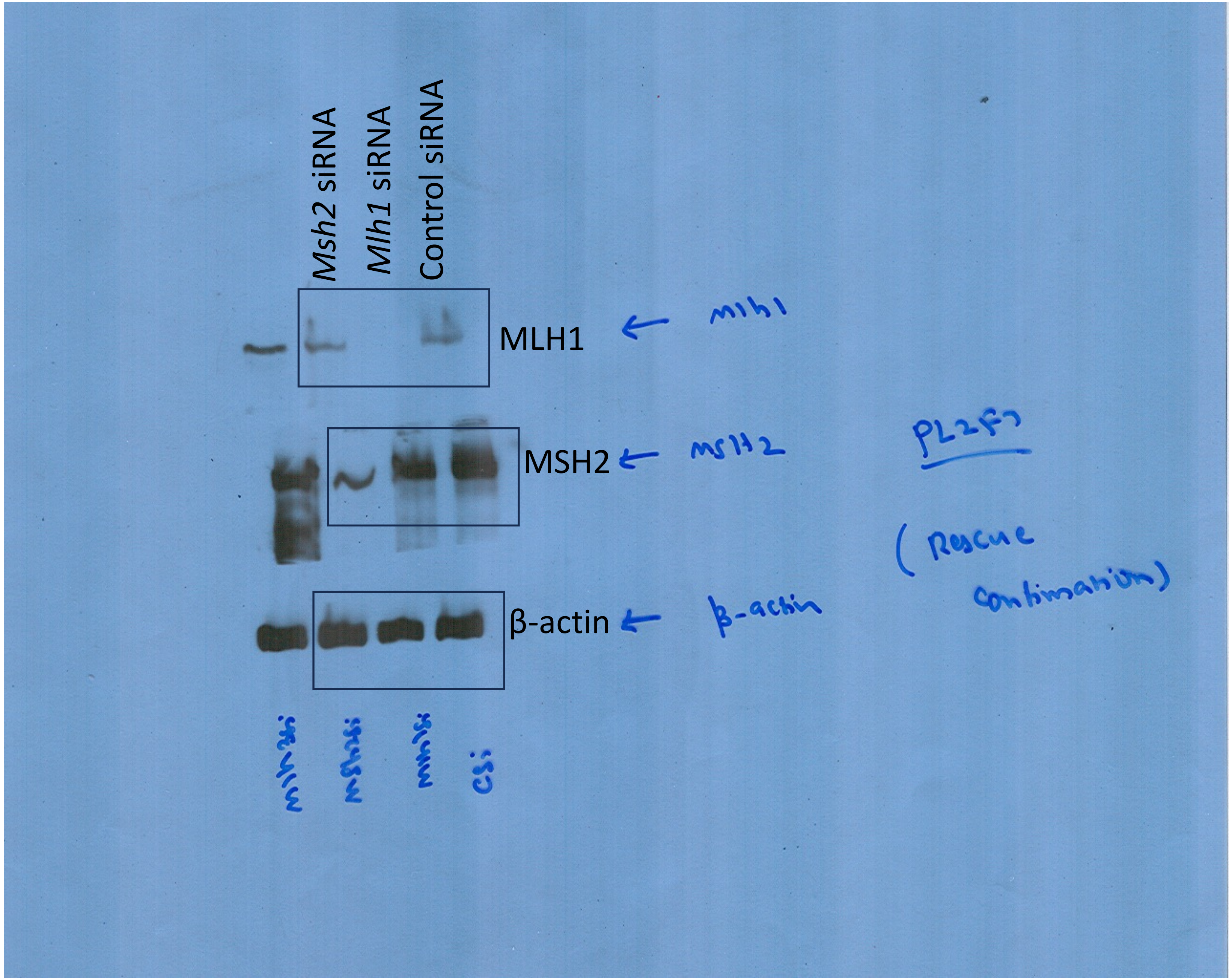

Full unedited blot for Figure 2D

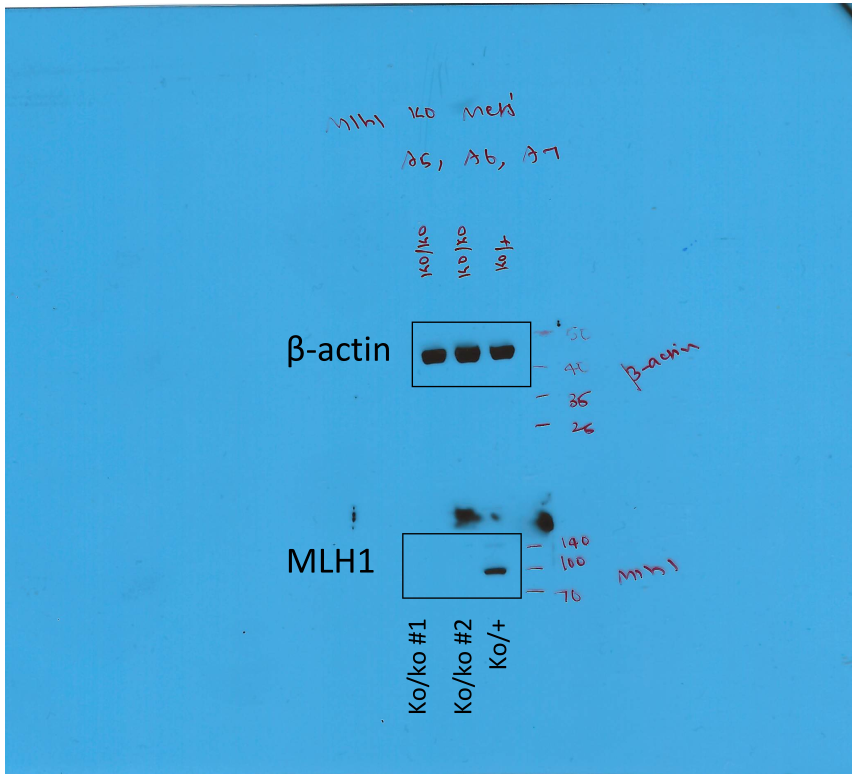

Full unedited blot for Supplemental Figure 4F

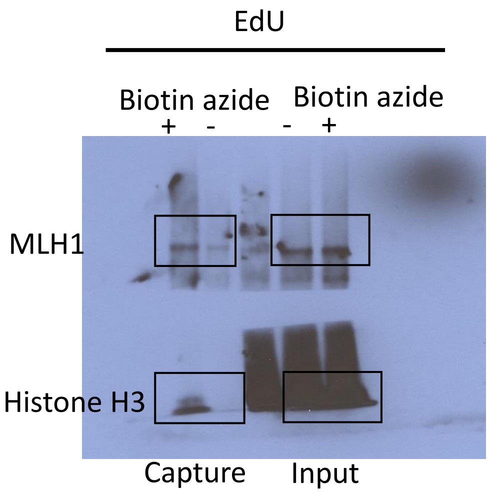

Full unedited blot for Figure 3C

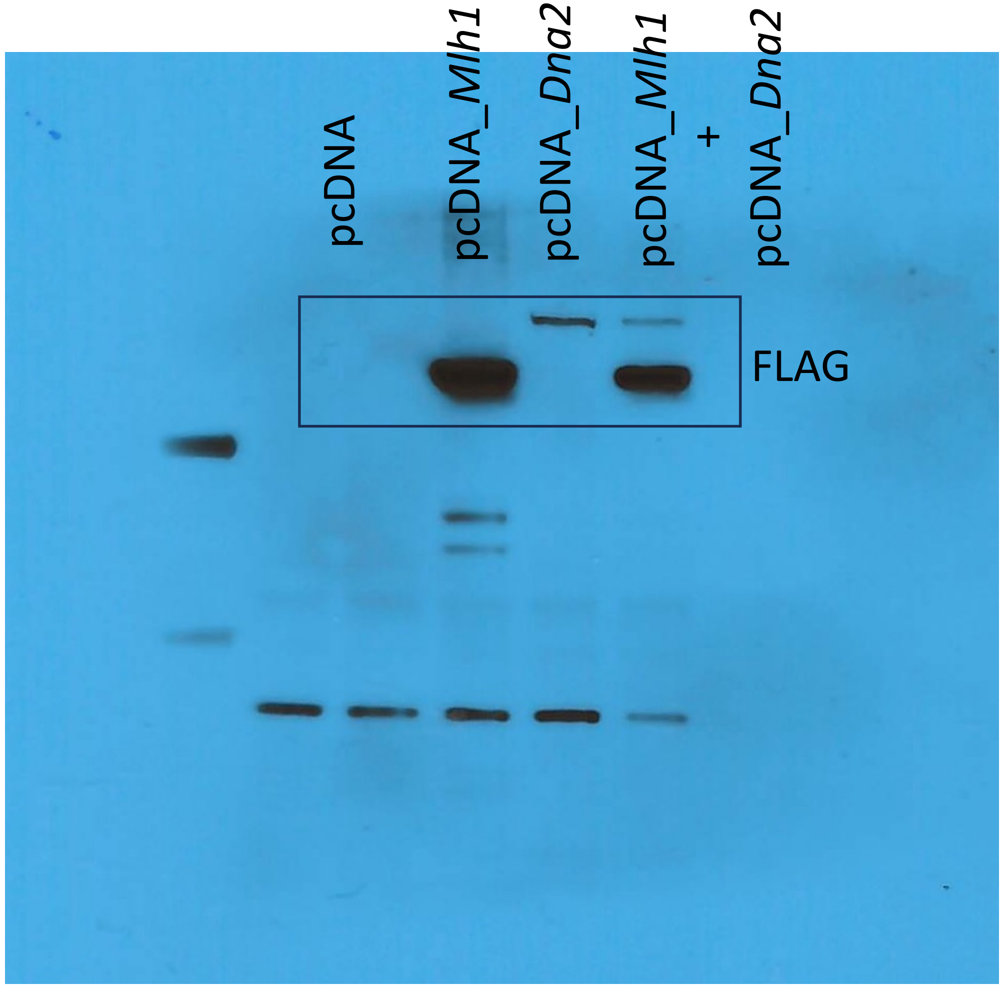

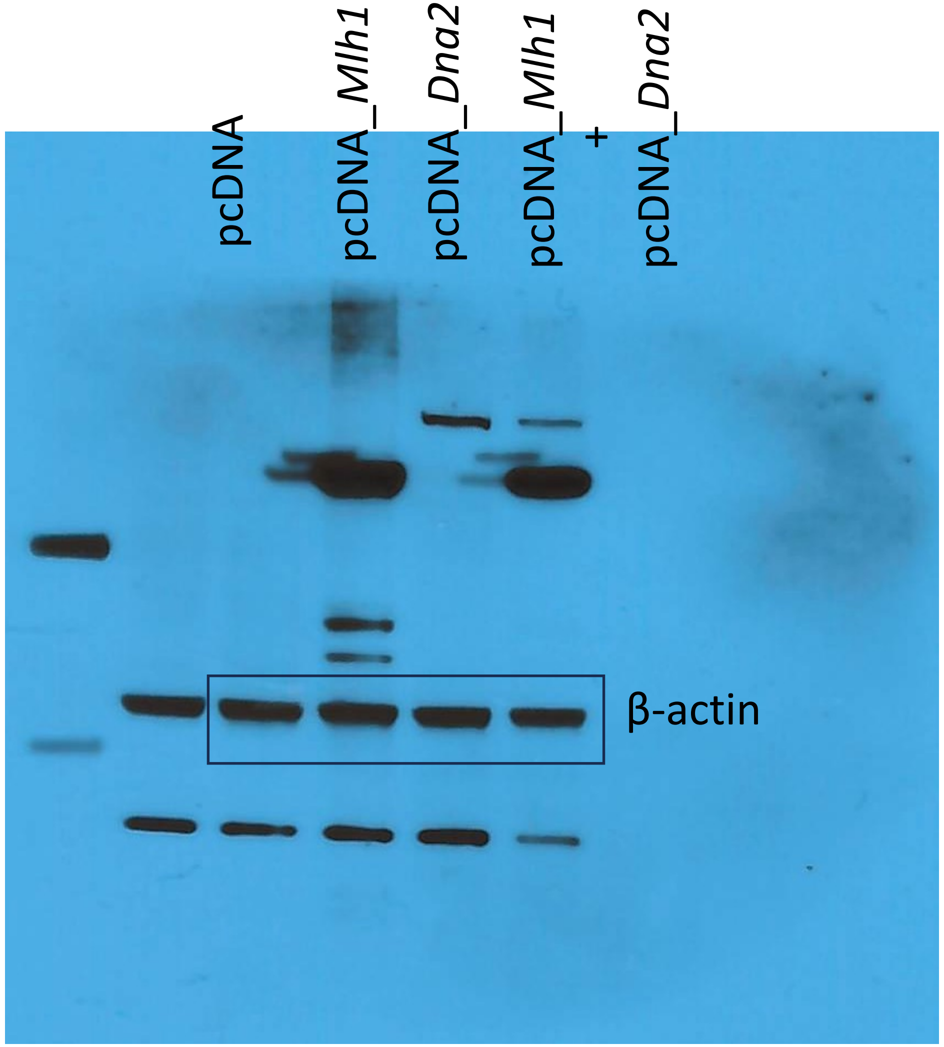

Full unedited blot for Figure 3F

-	+	+	+	+	+	RNA flap substrate
-	+	+	-	+	+	MLH1
-	-	-	+	+	+	FEN1

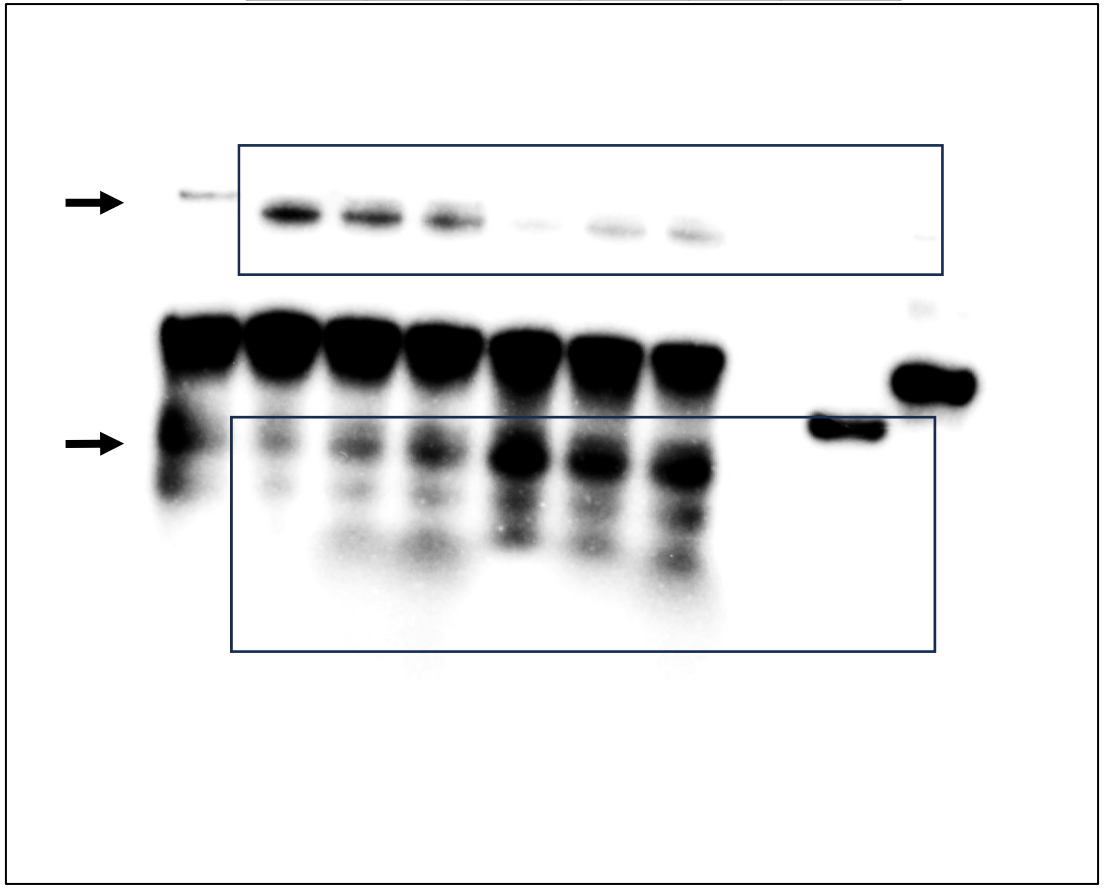

Full unedited blot for Supplemental Figure 6C

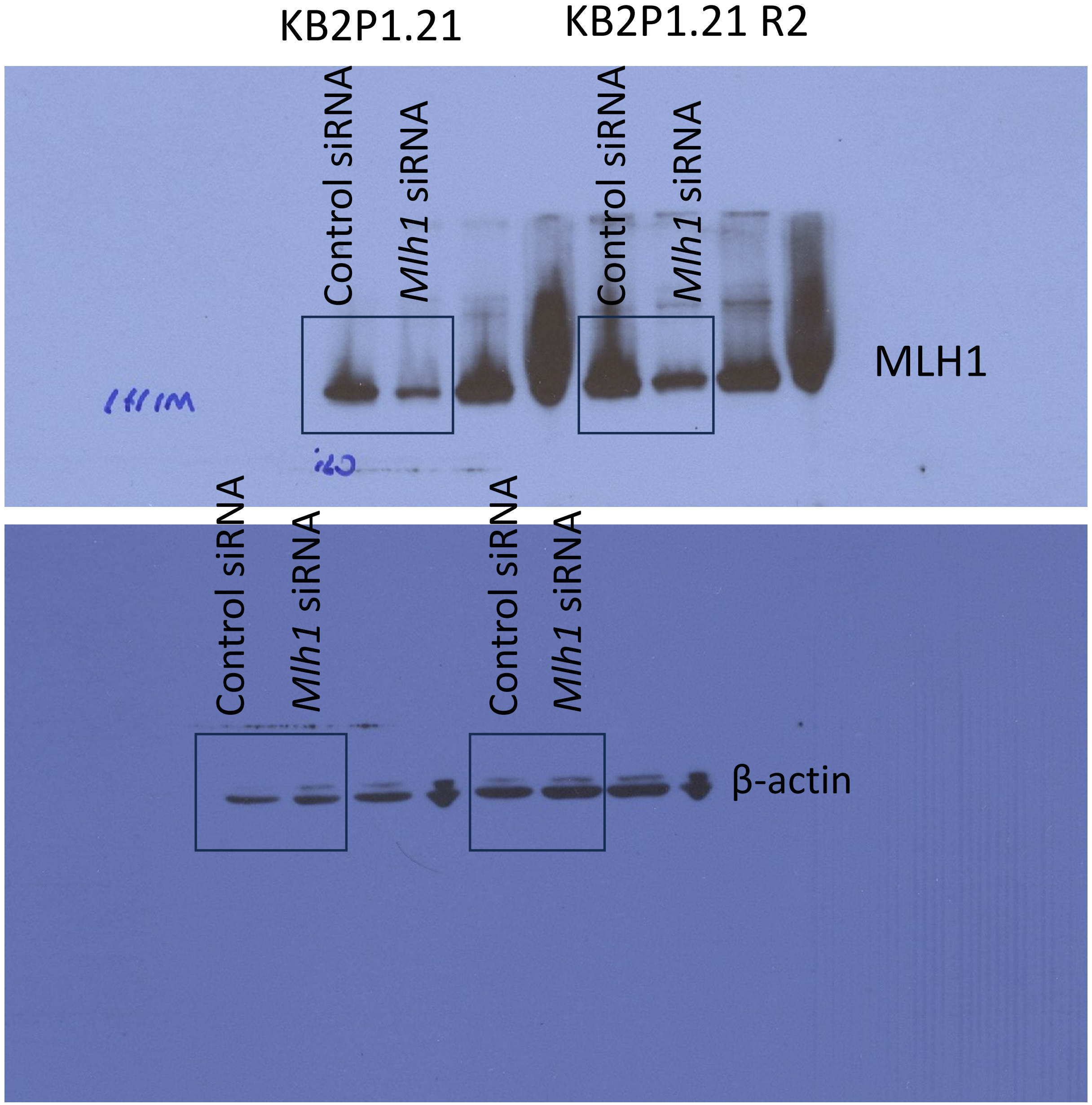

Full unedited blot for Supplemental Figure 6E

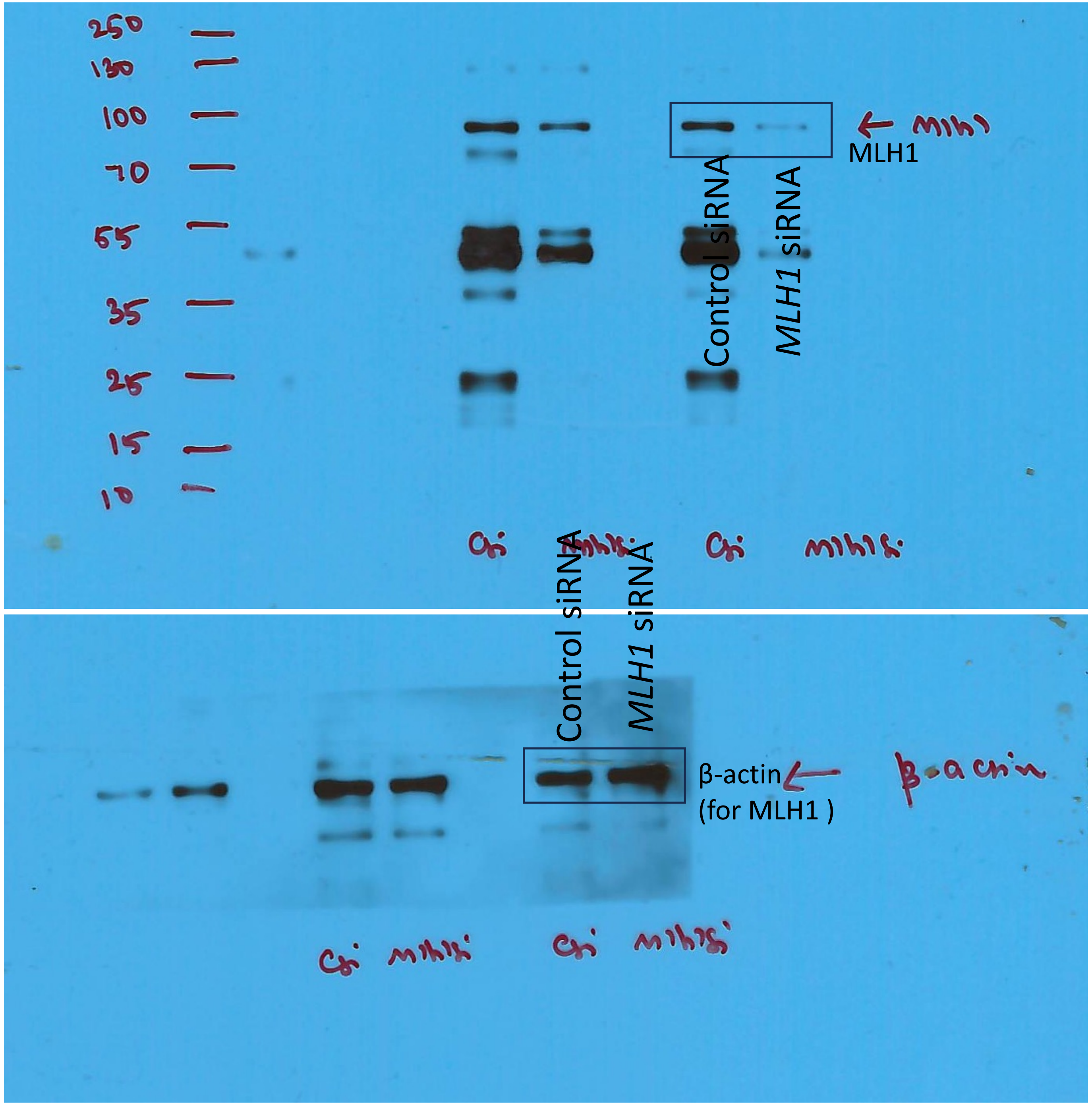

Full unedited blot for Supplemental Figure 6E

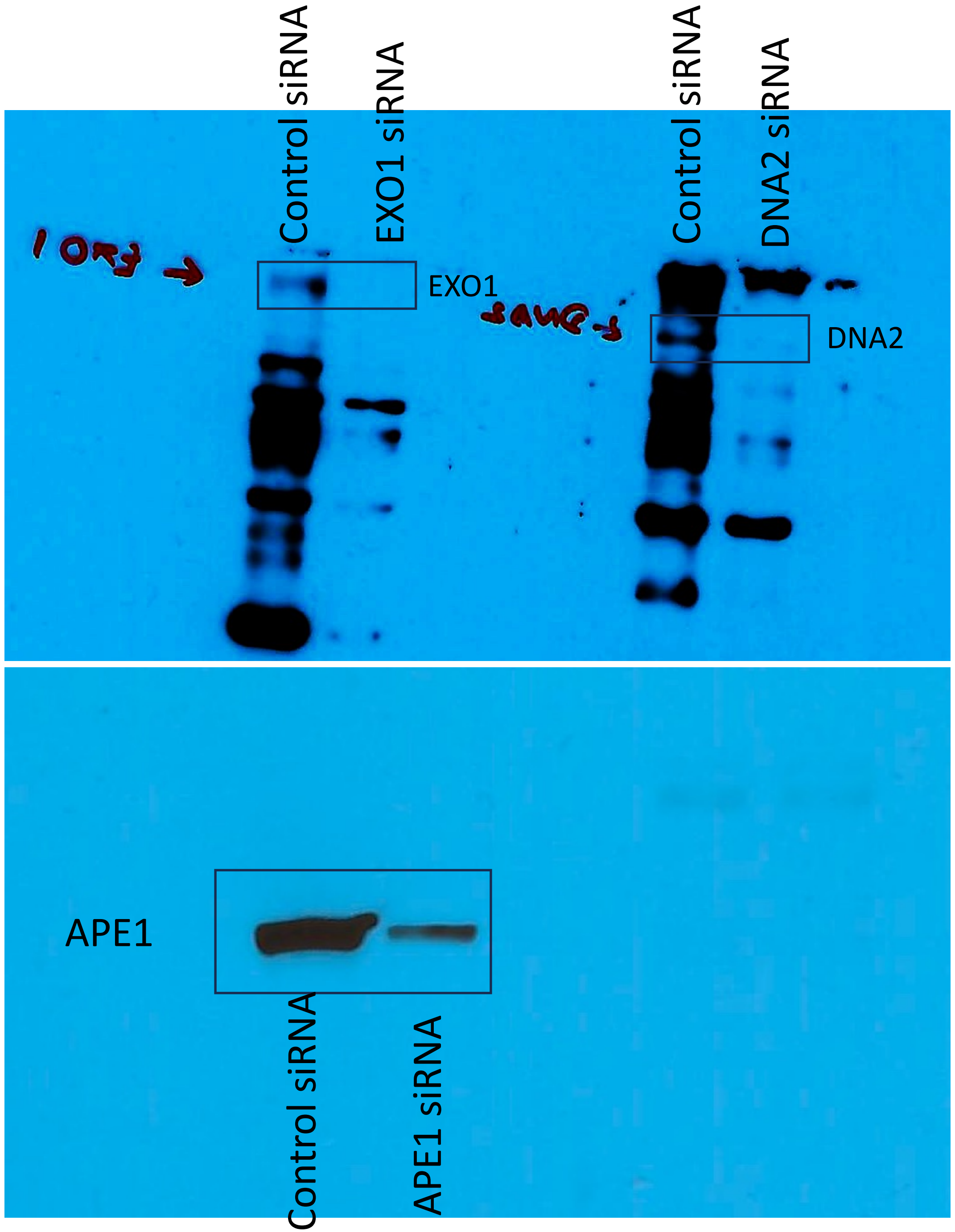

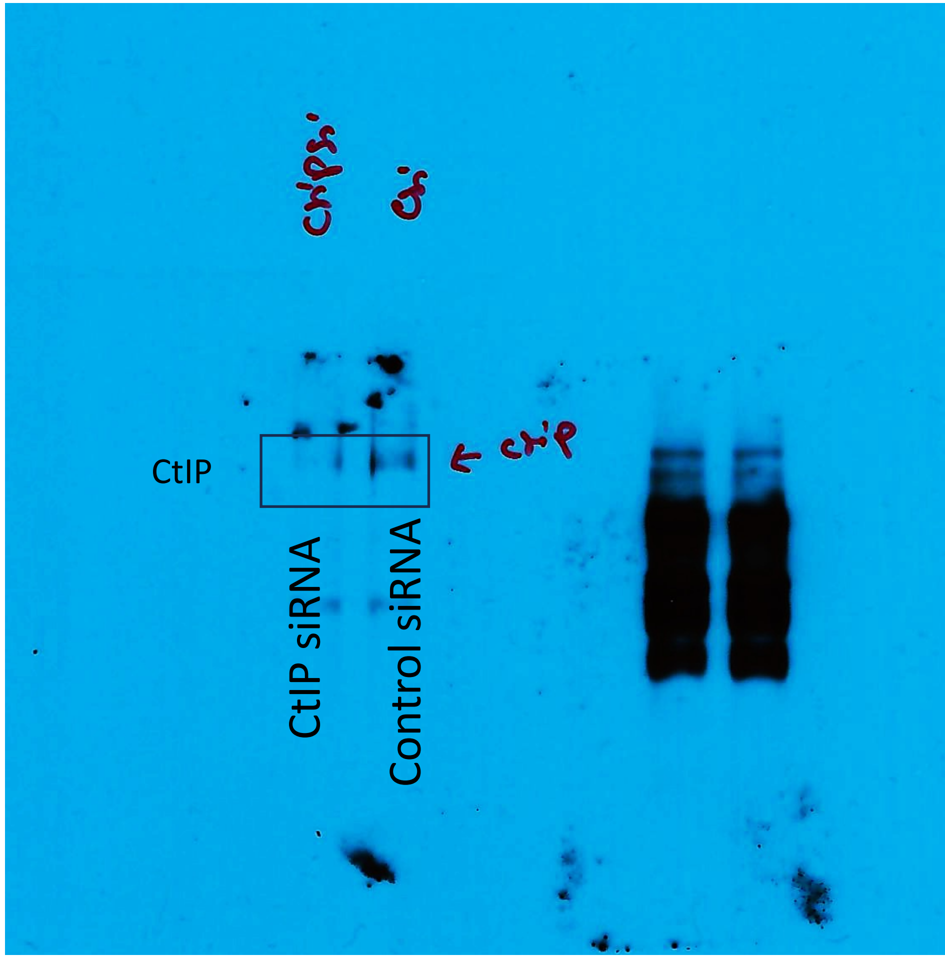

Full unedited blot for Supplemental Figure 6E

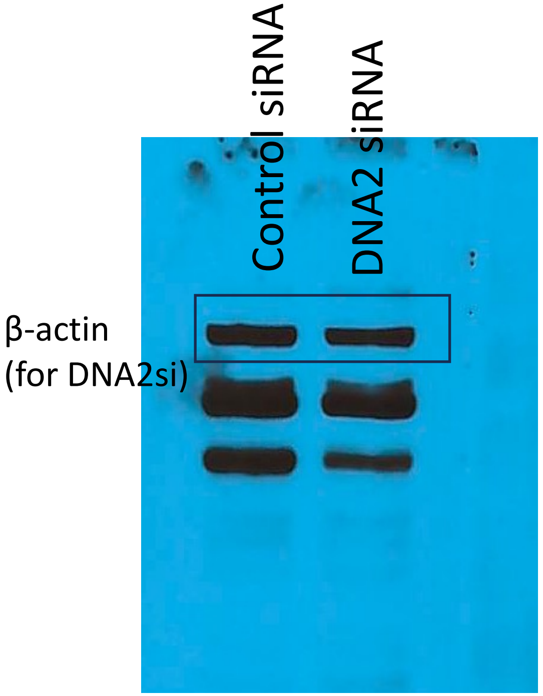

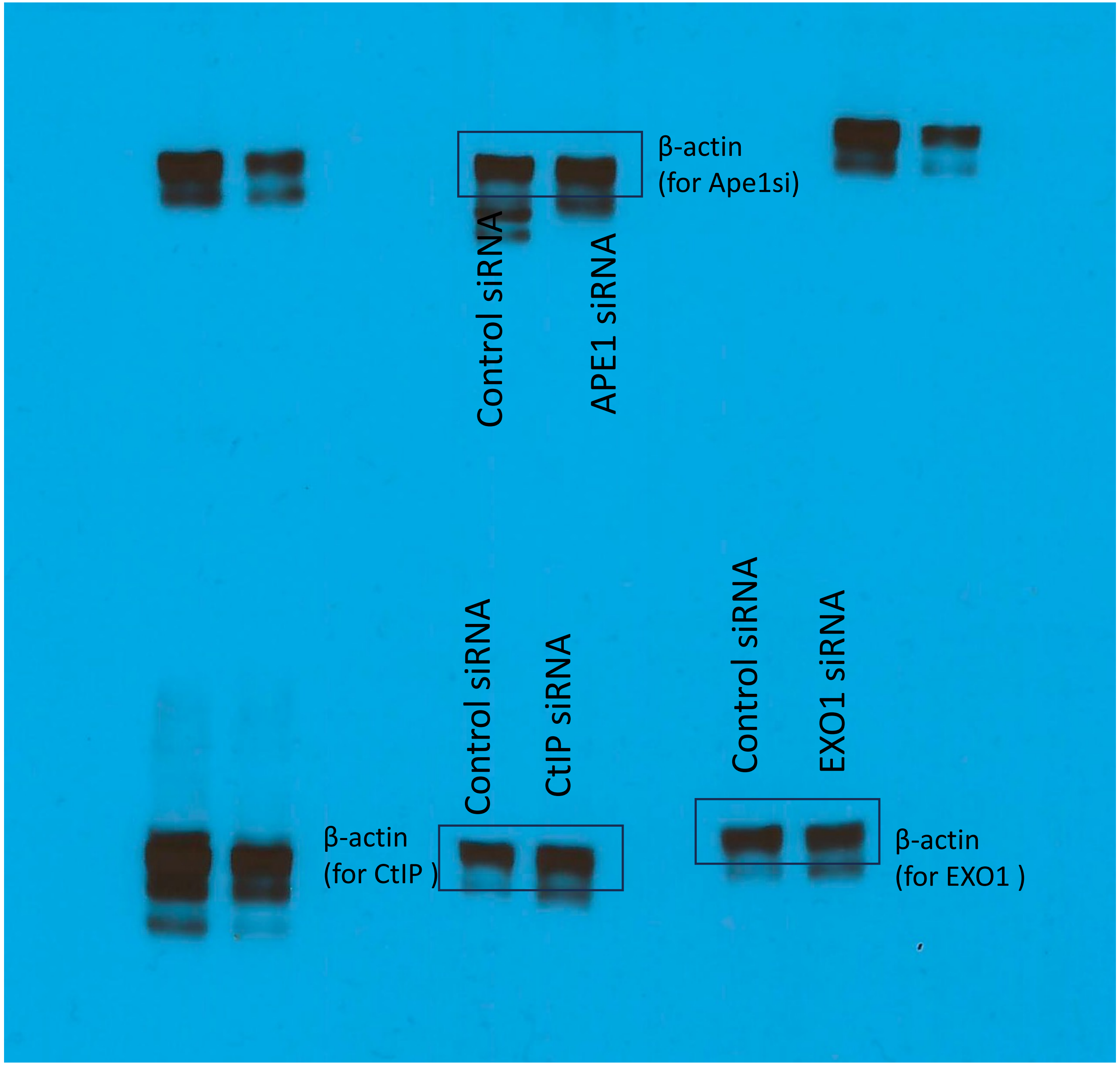

Full unedited blot for Supplemental Figure 8A

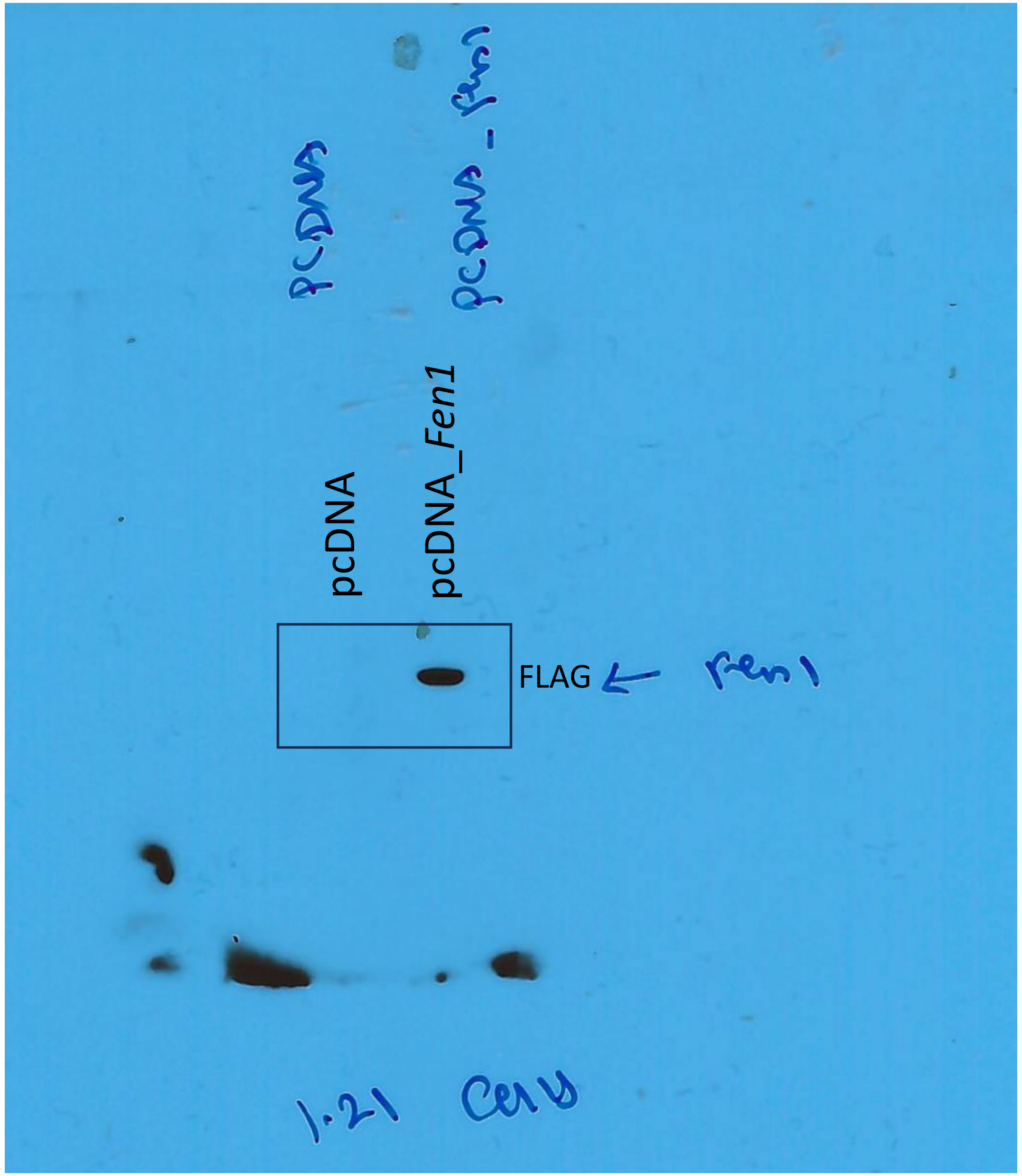

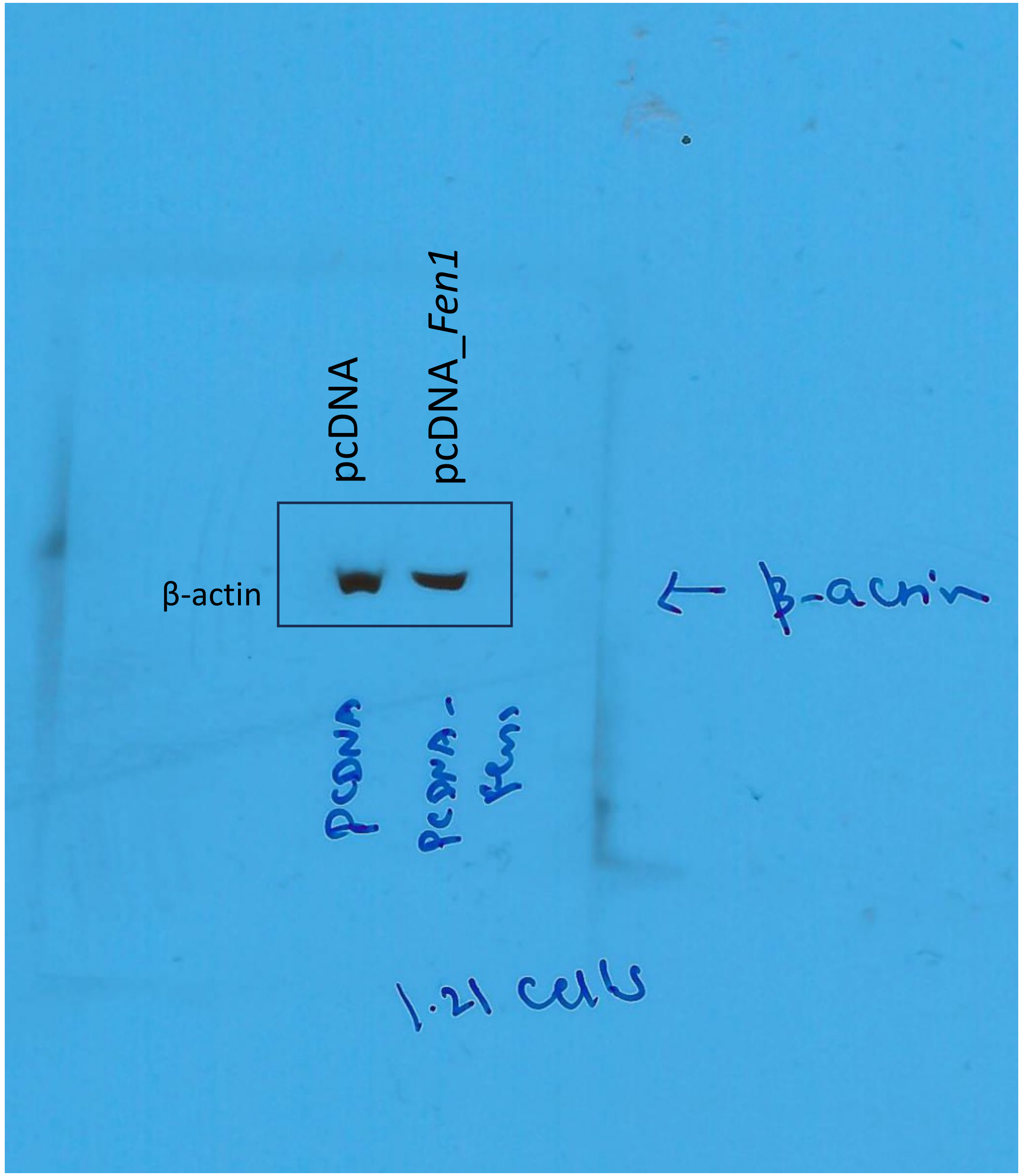

Full unedited blot for Supplemental Figure 8C

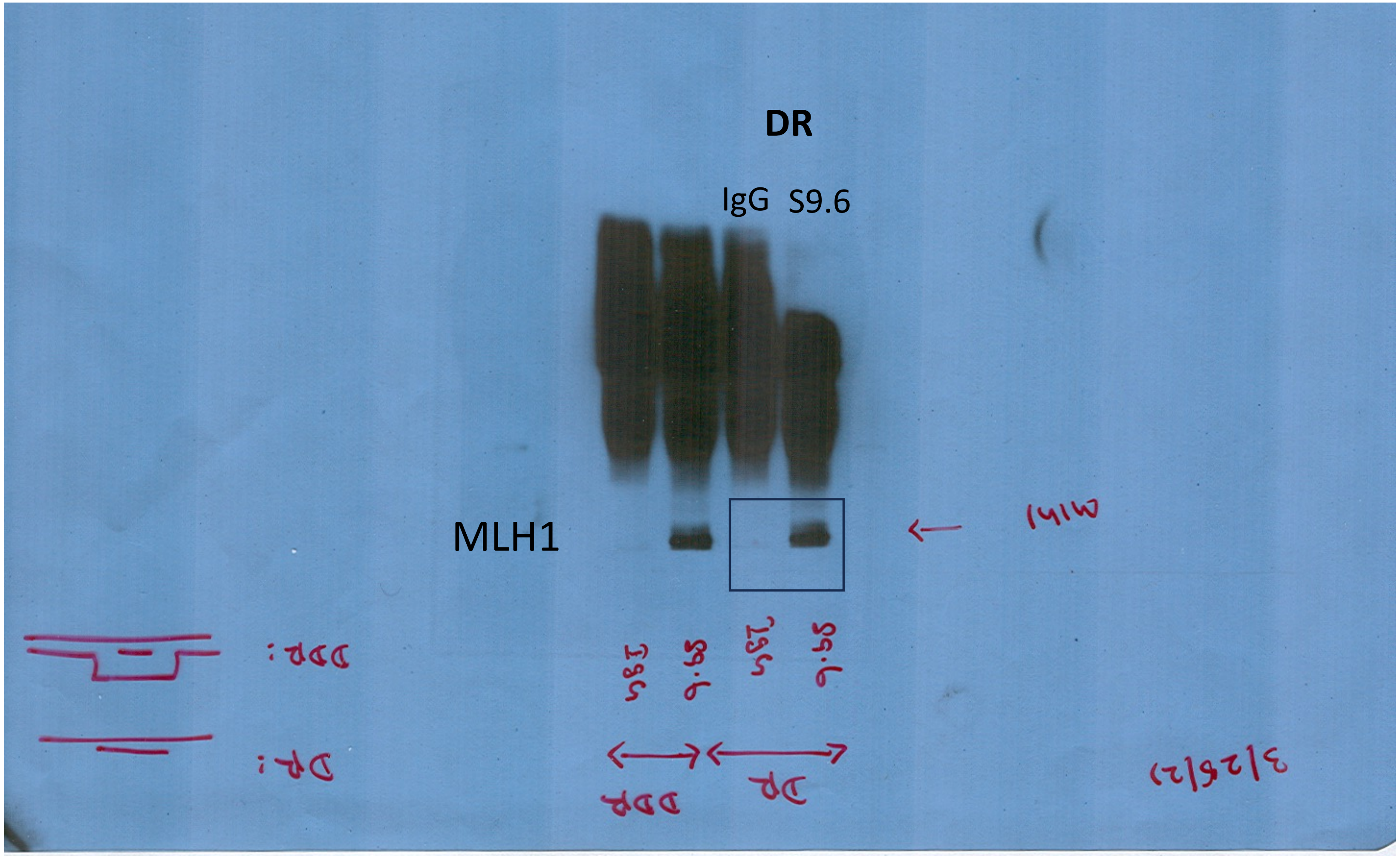

Full unedited blot for Supplemental Figure 8D

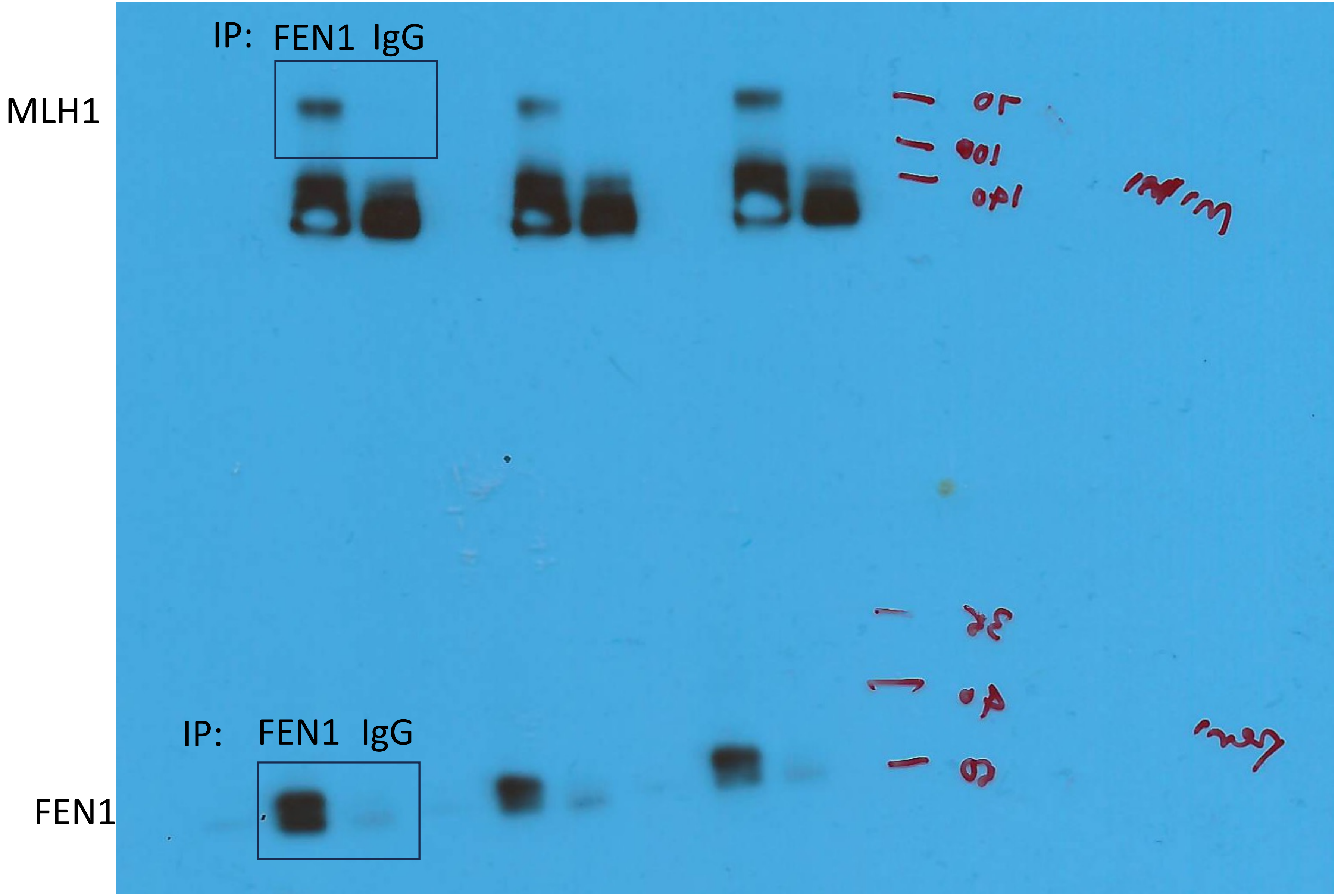

Full unedited blot for Supplemental Figure 8D

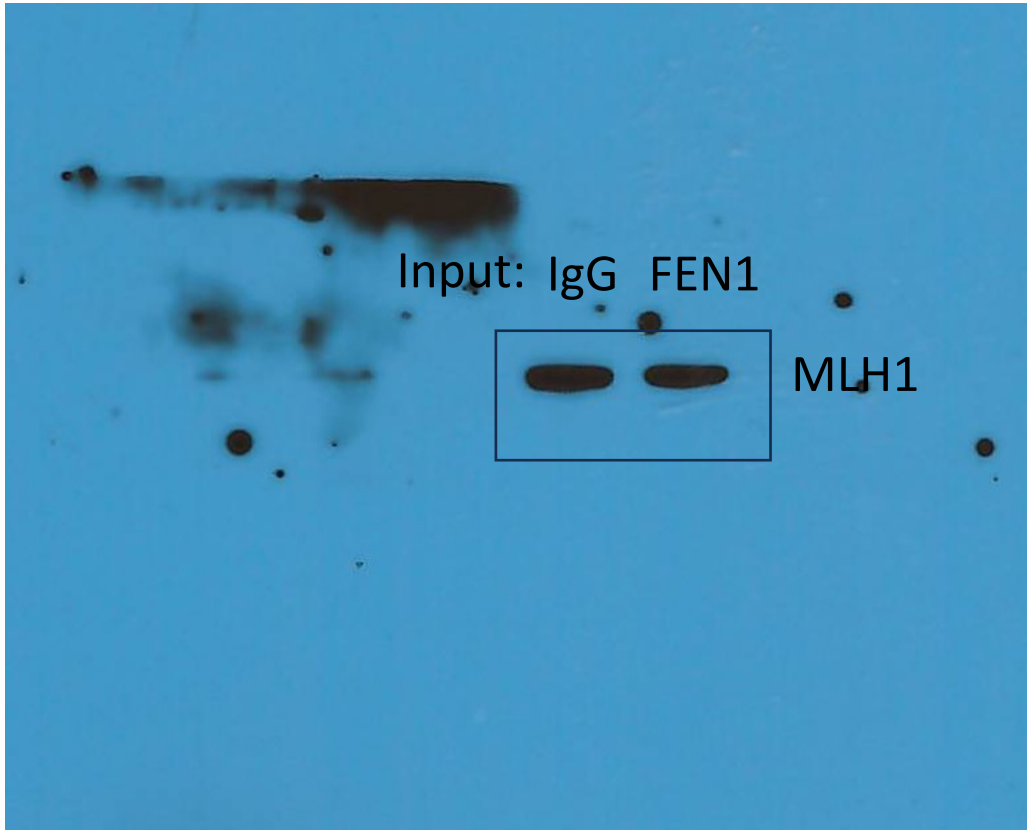

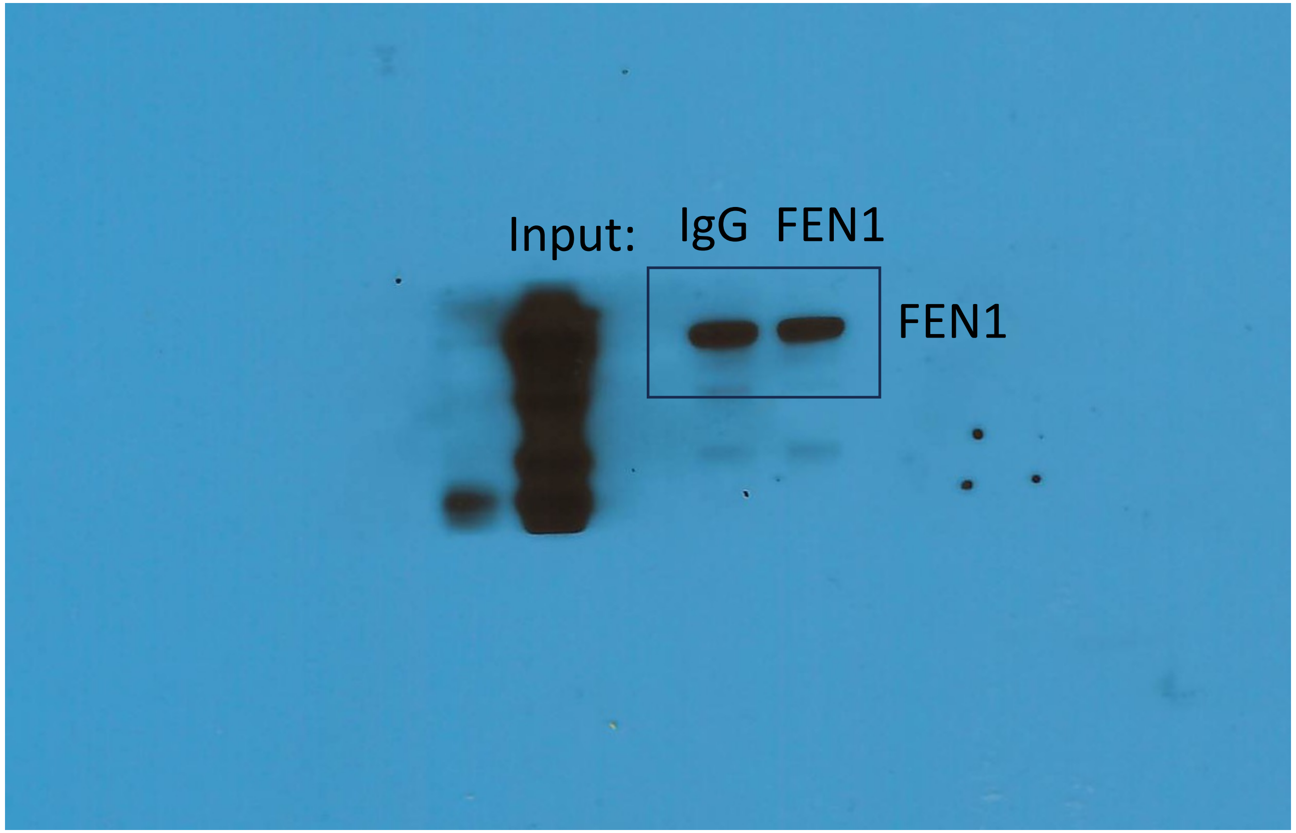

Full unedited gel for Supplemental Figure 8E

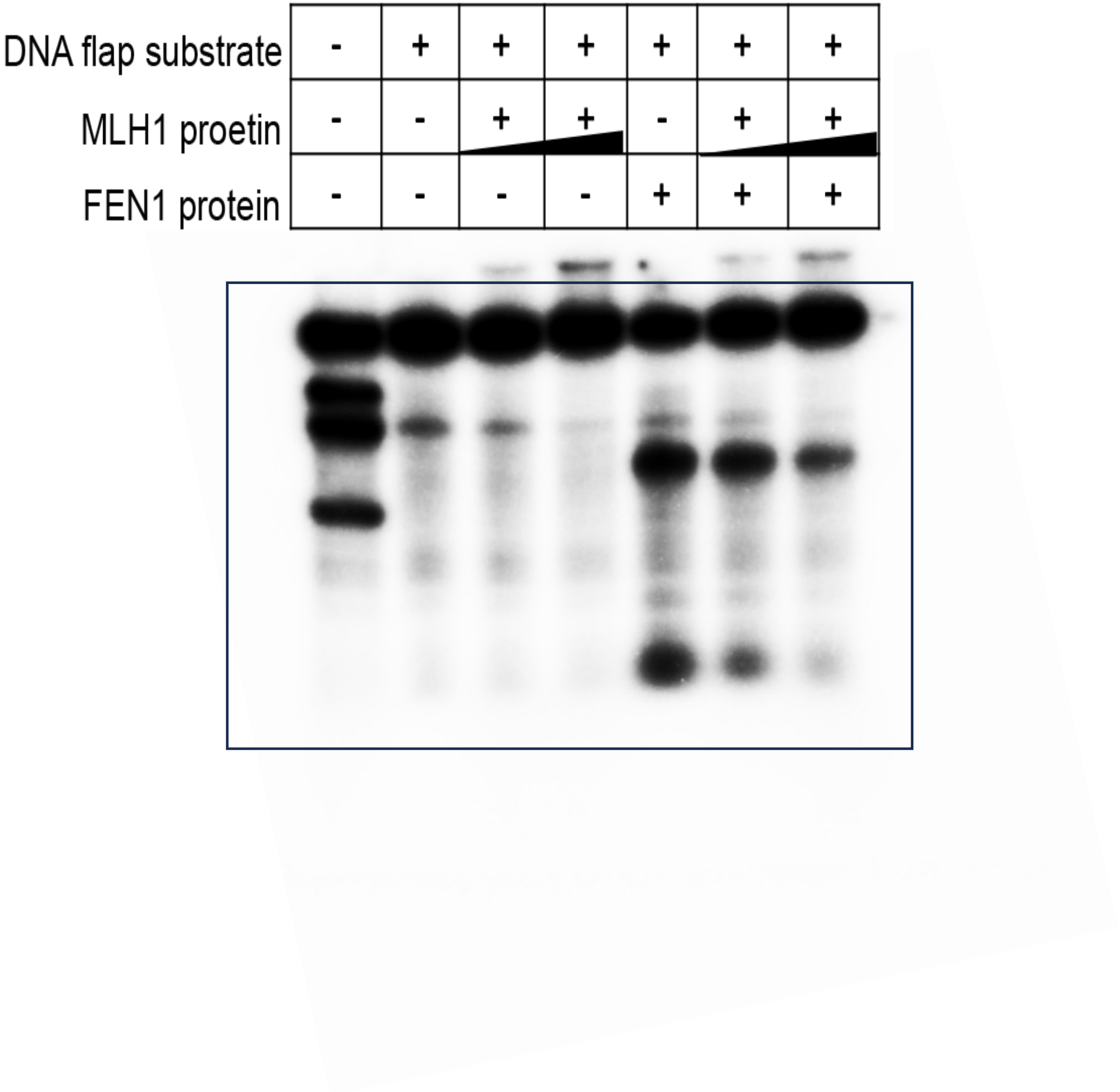

Full unedited gel for Supplemental Figure 8F

-	+	+	+	DNA flap substrate
-	-	+	+	MLH1 proetin
-	-	-	+	FEN1 protein

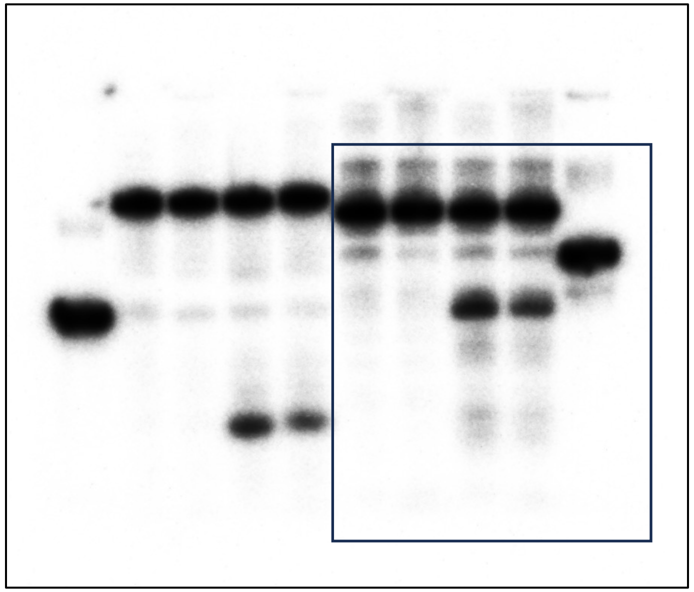

Full unedited blot for Supplemental Figure 8G

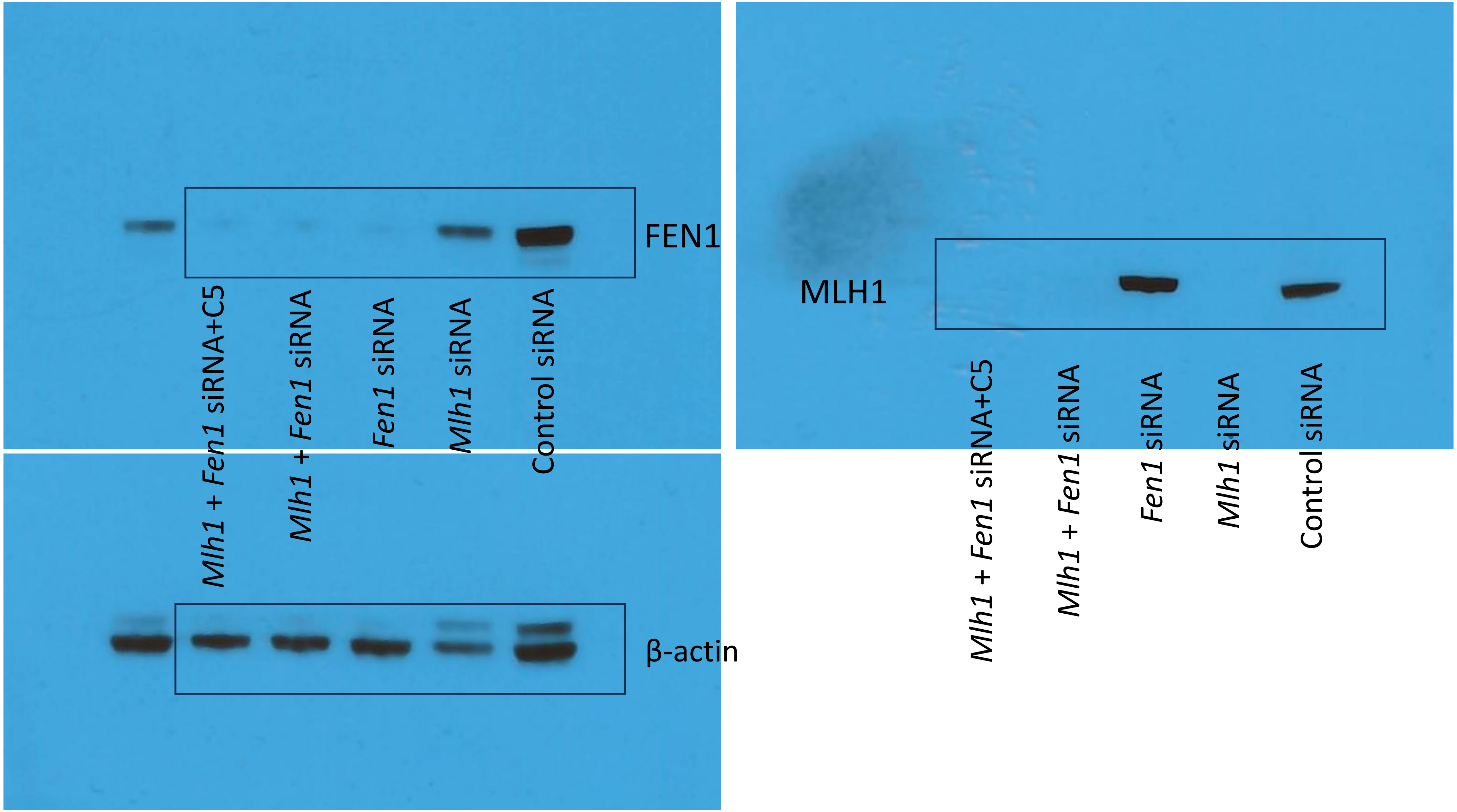

Full unedited blot for Supplemental Figure 8H

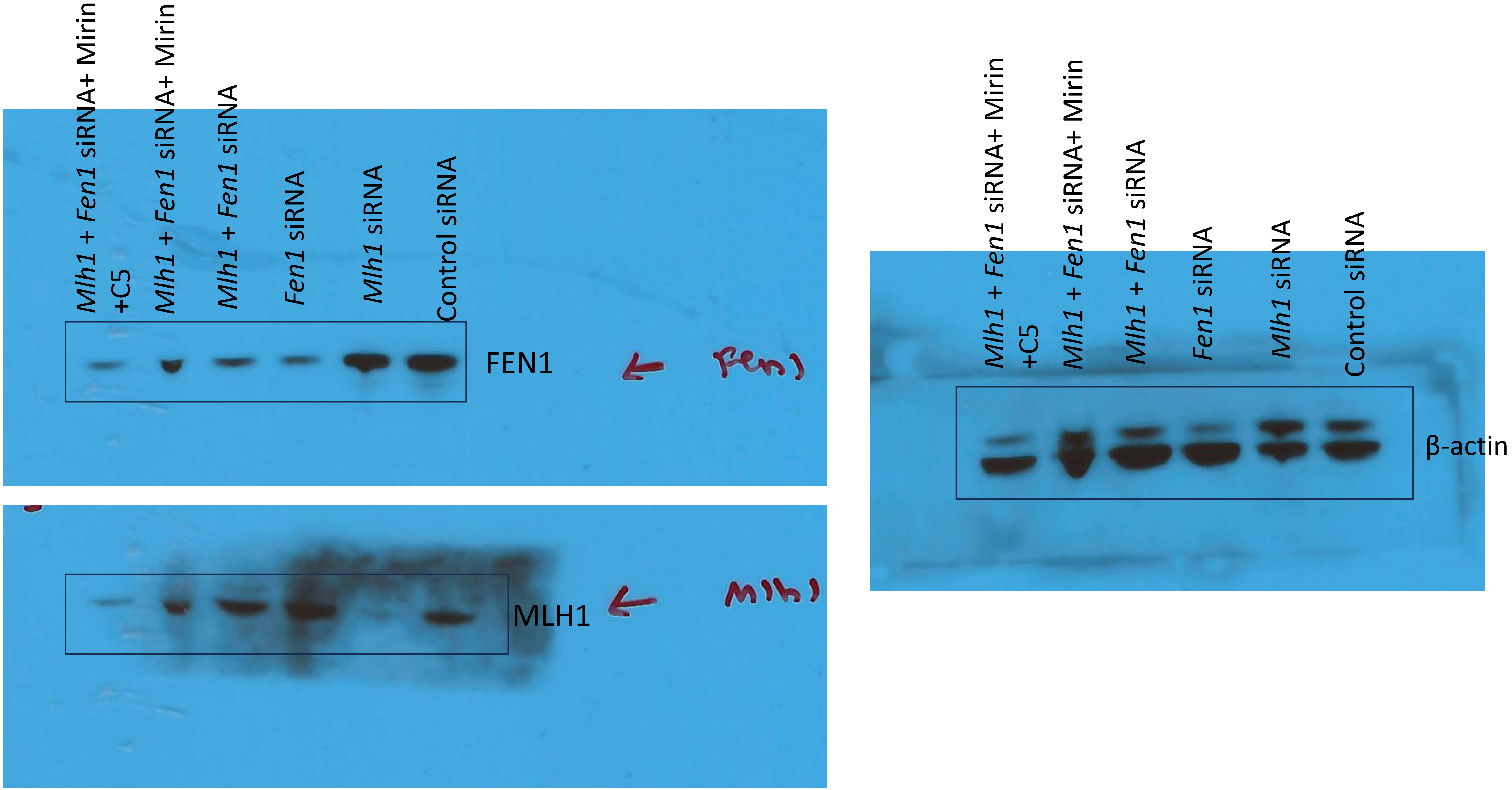

Full unedited blot for Figure 4D

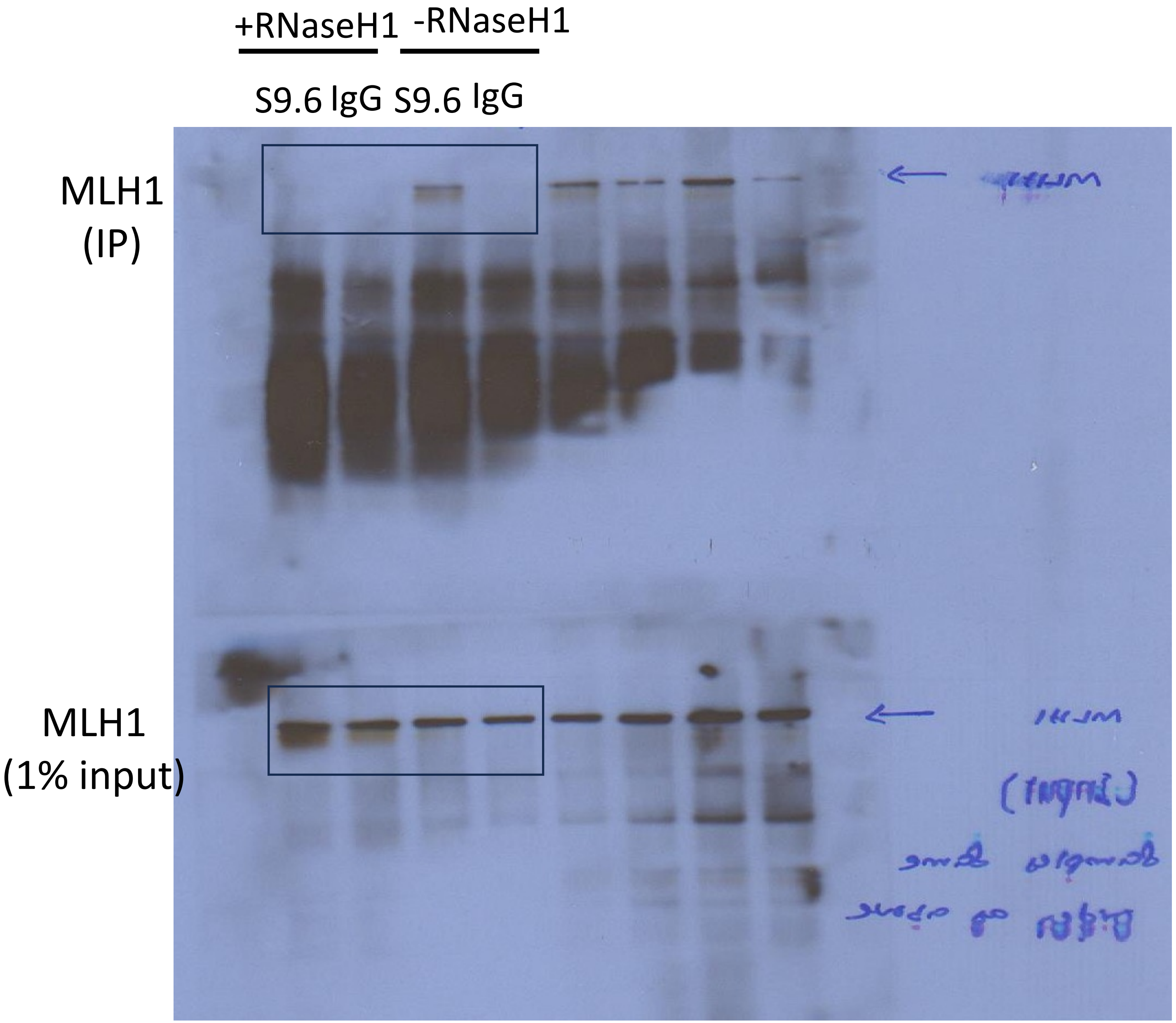

Full unedited blot for Figure 4E

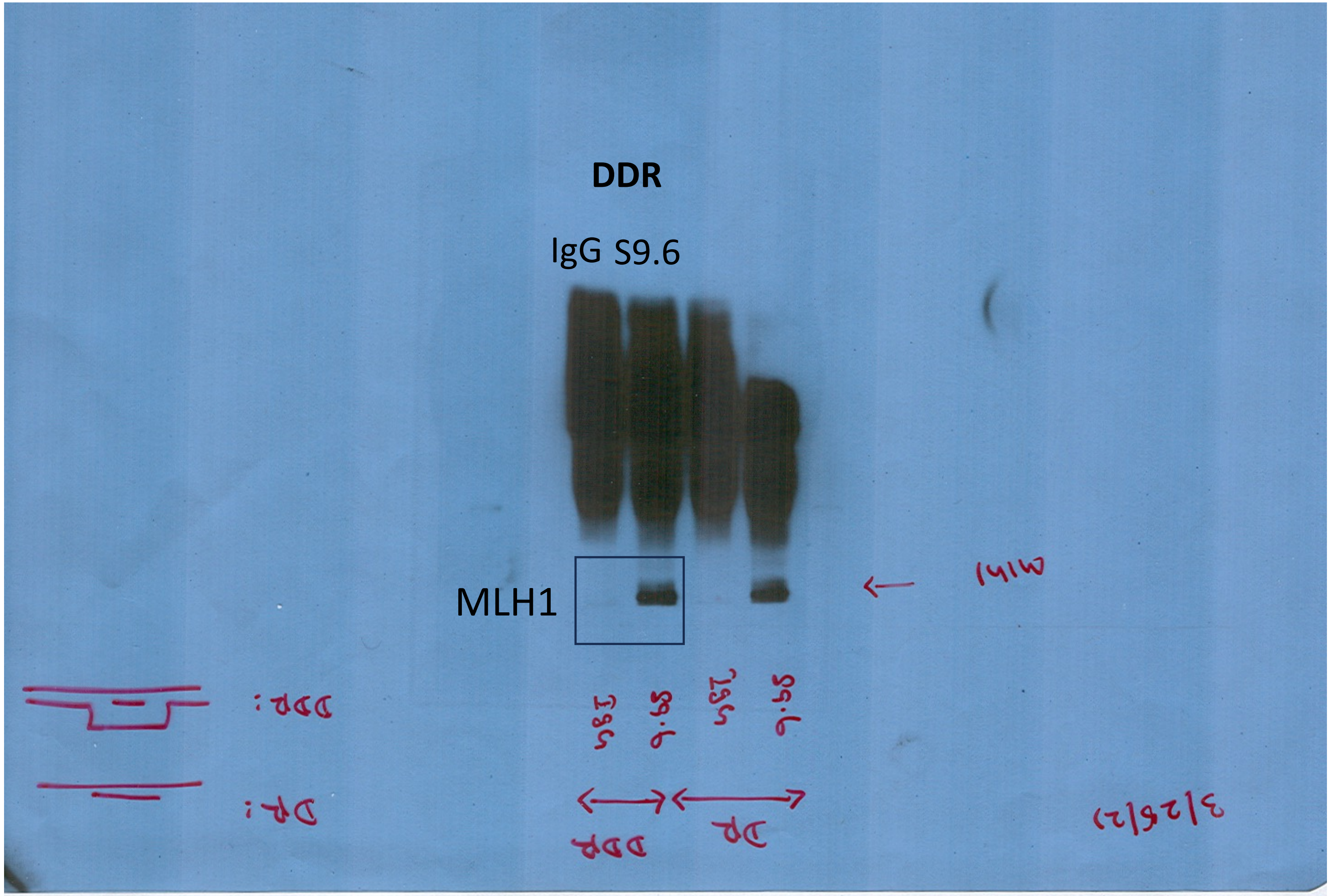

Full unedited gel for Figure 4F

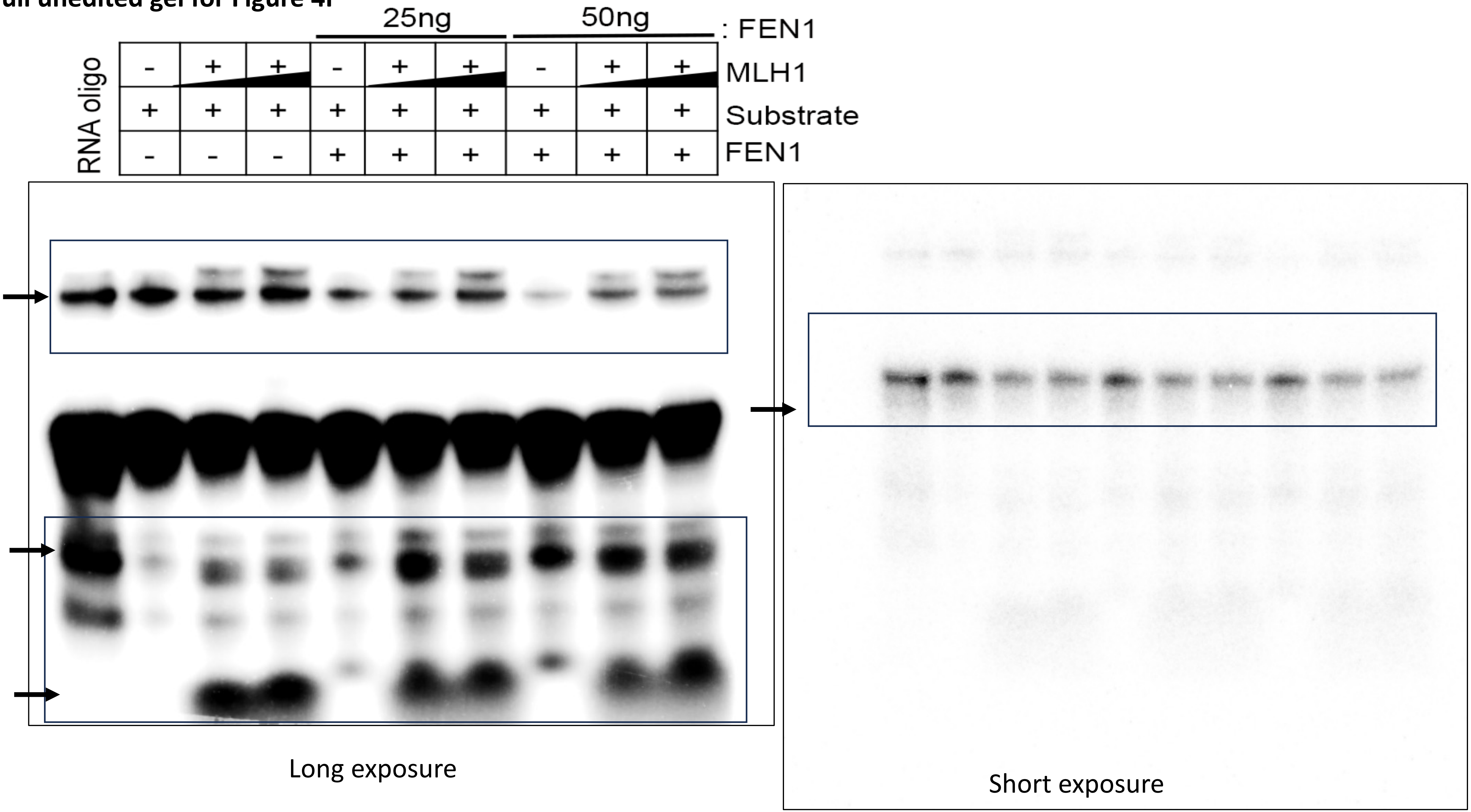

Full unedited blot for Figure 4H

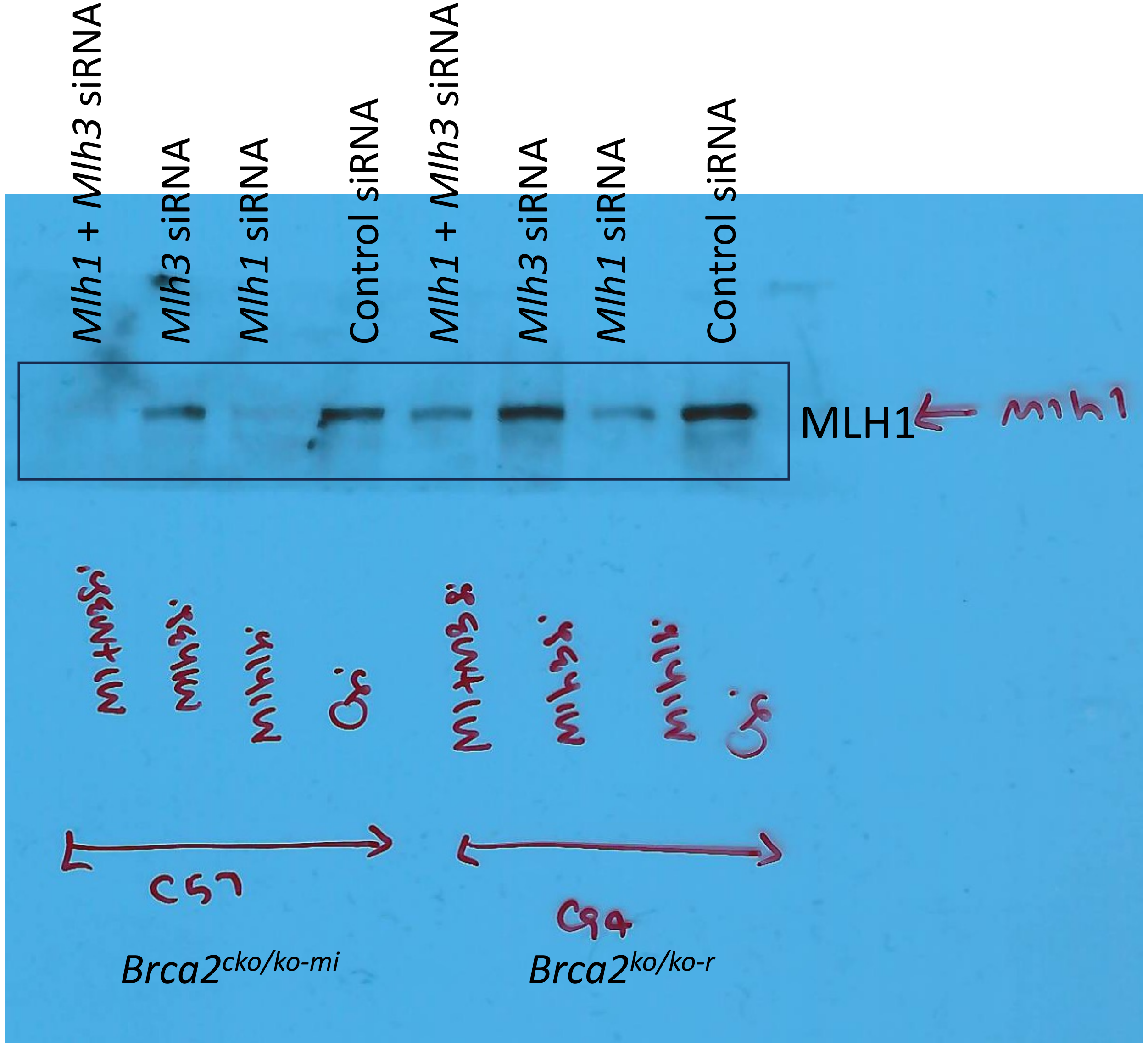

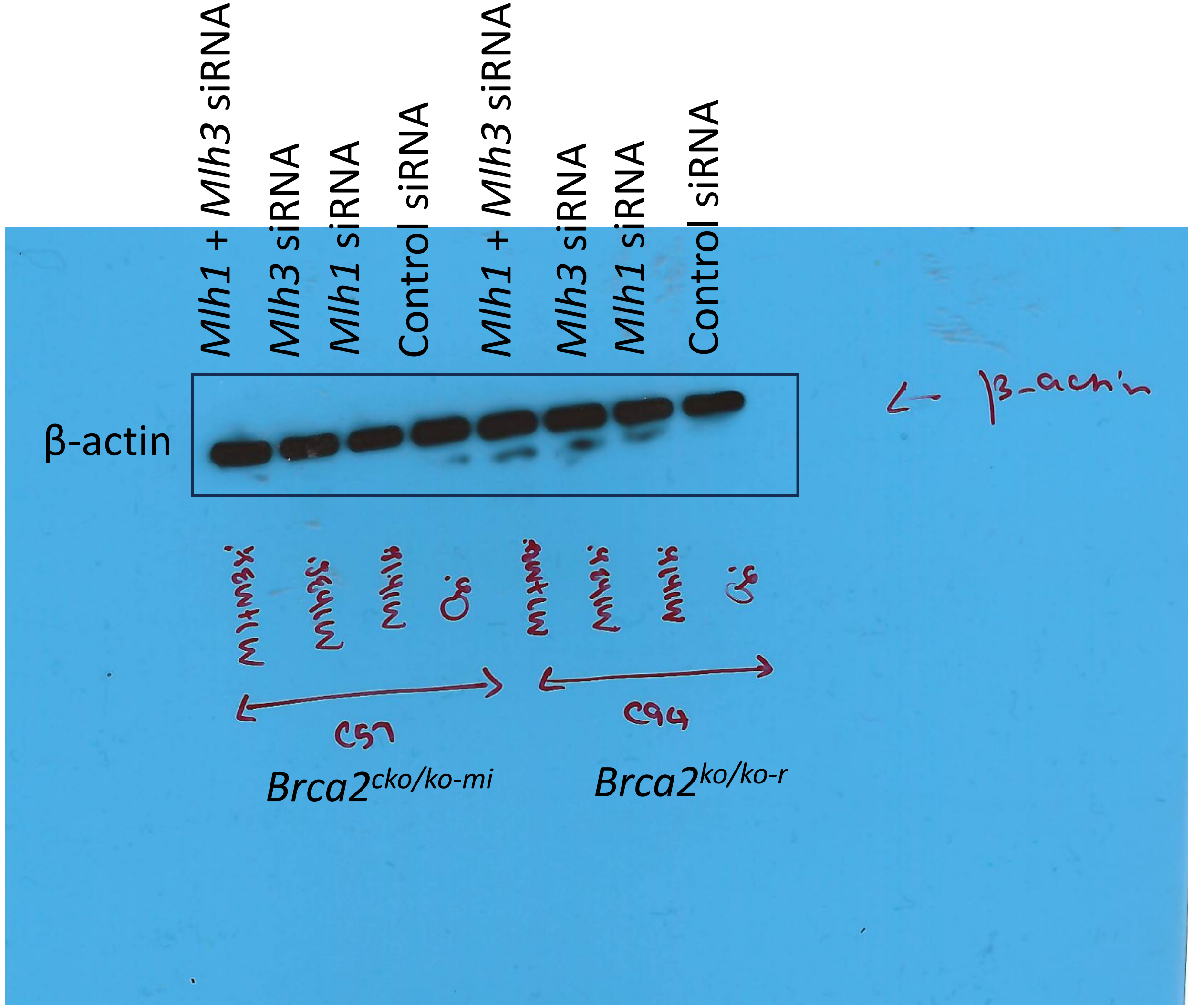

Full unedited blot for Figure 6B

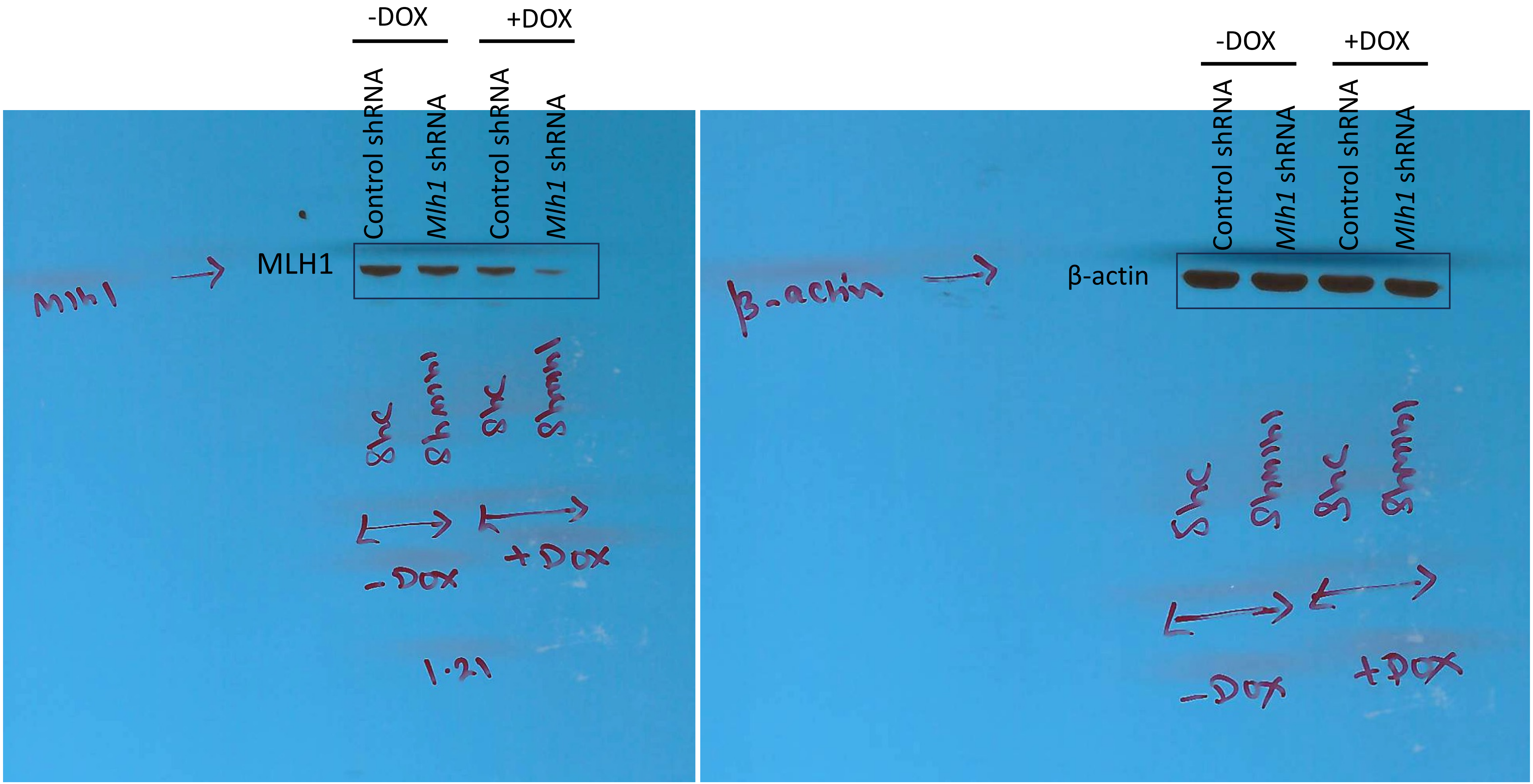

Full unedited blot for Figure 7B

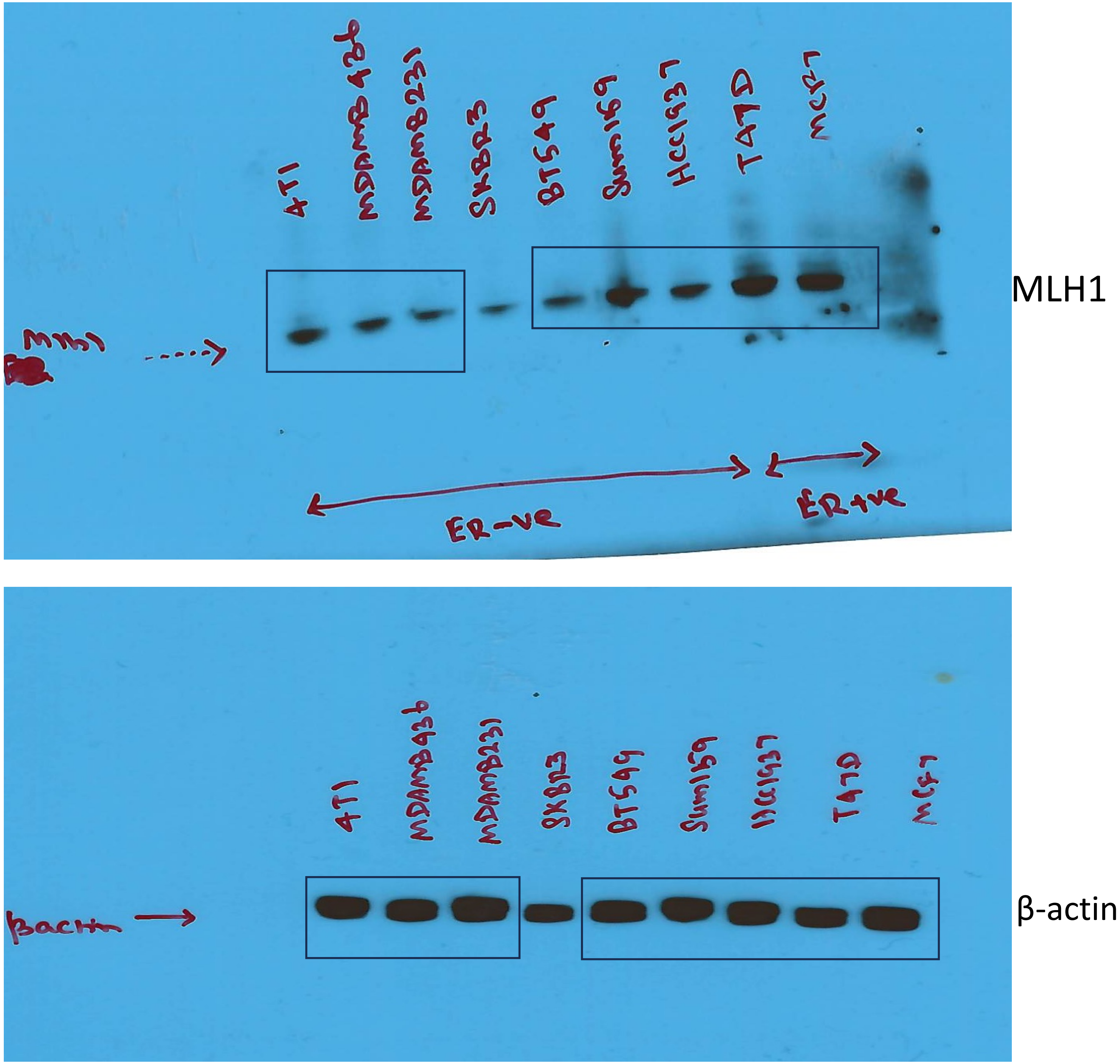

Full unedited blot for Figure 7C

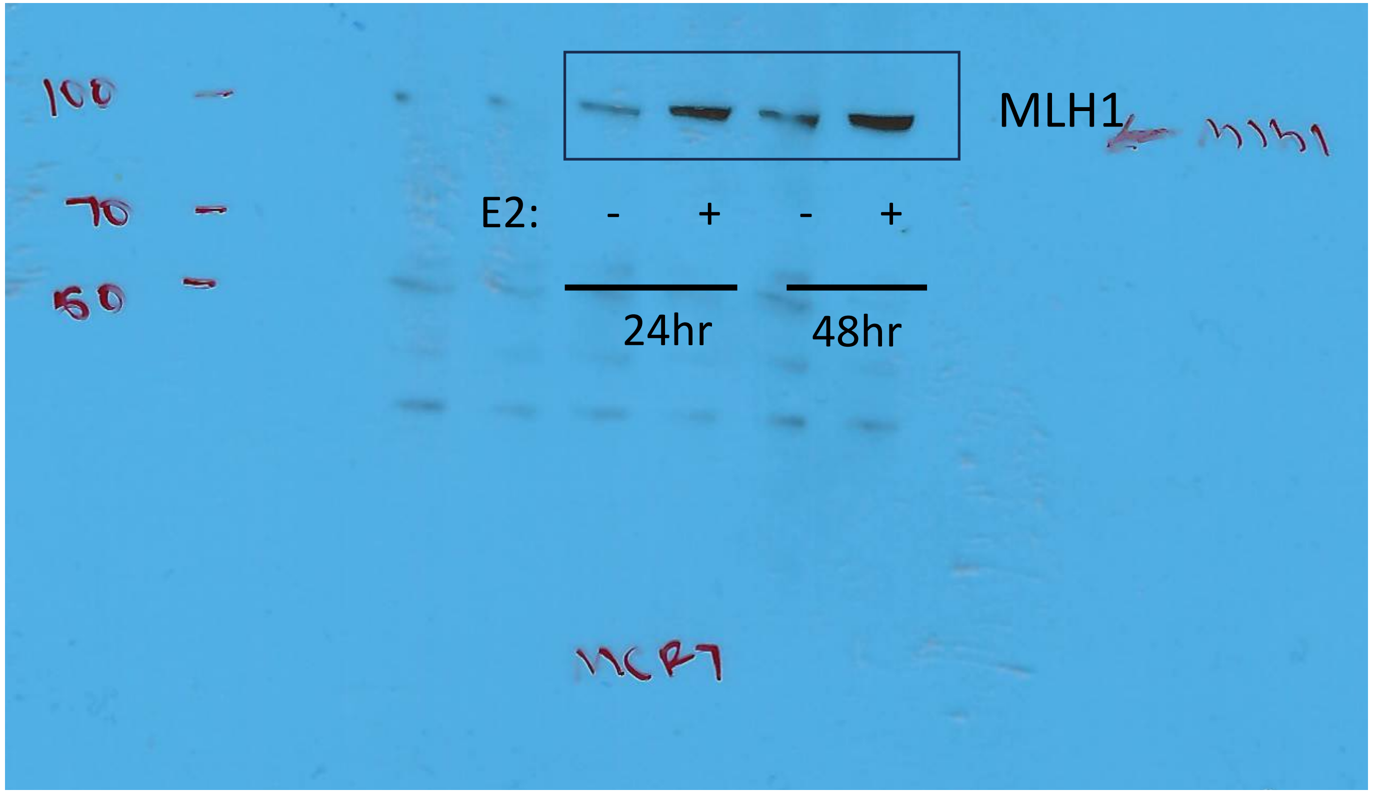

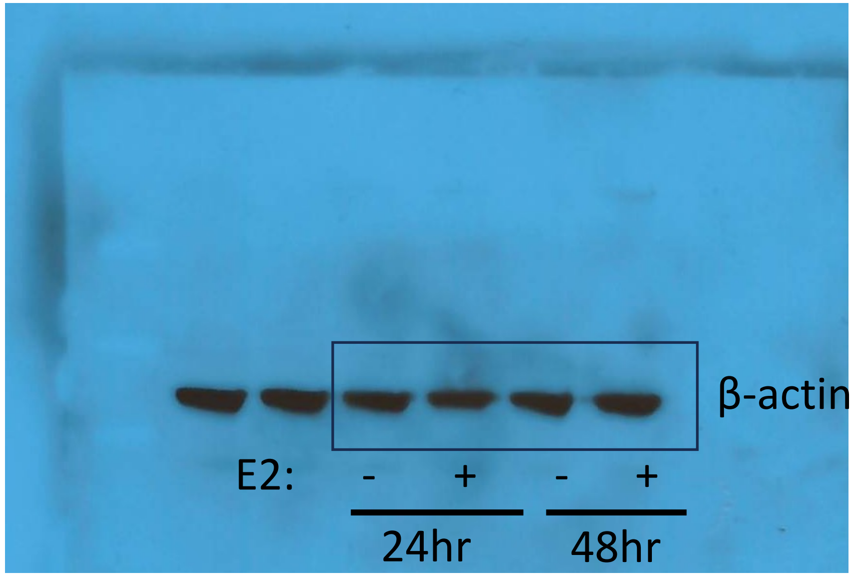

Full unedited blot for Figure 7D

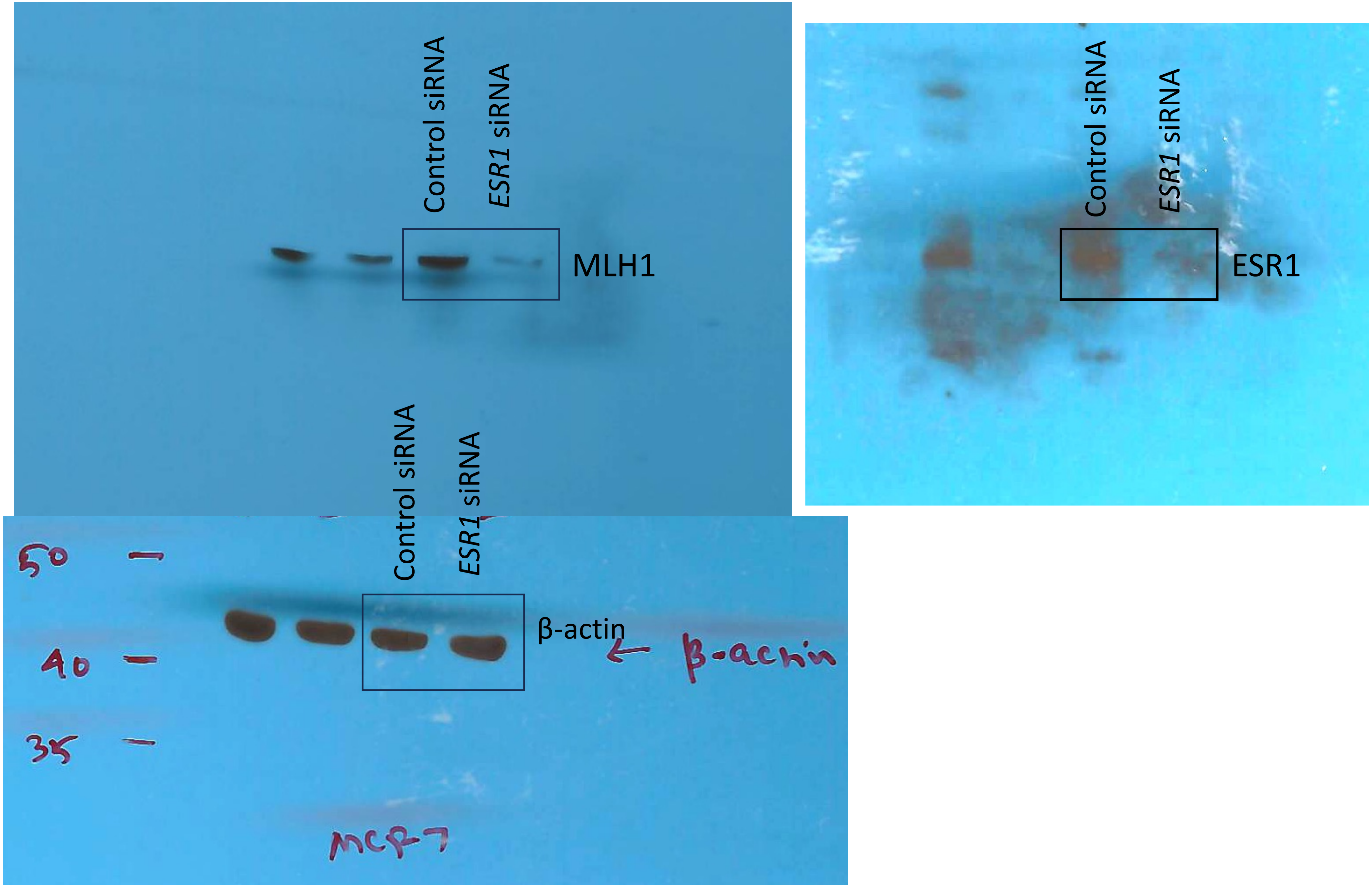

Full unedited blot for Figure 7E

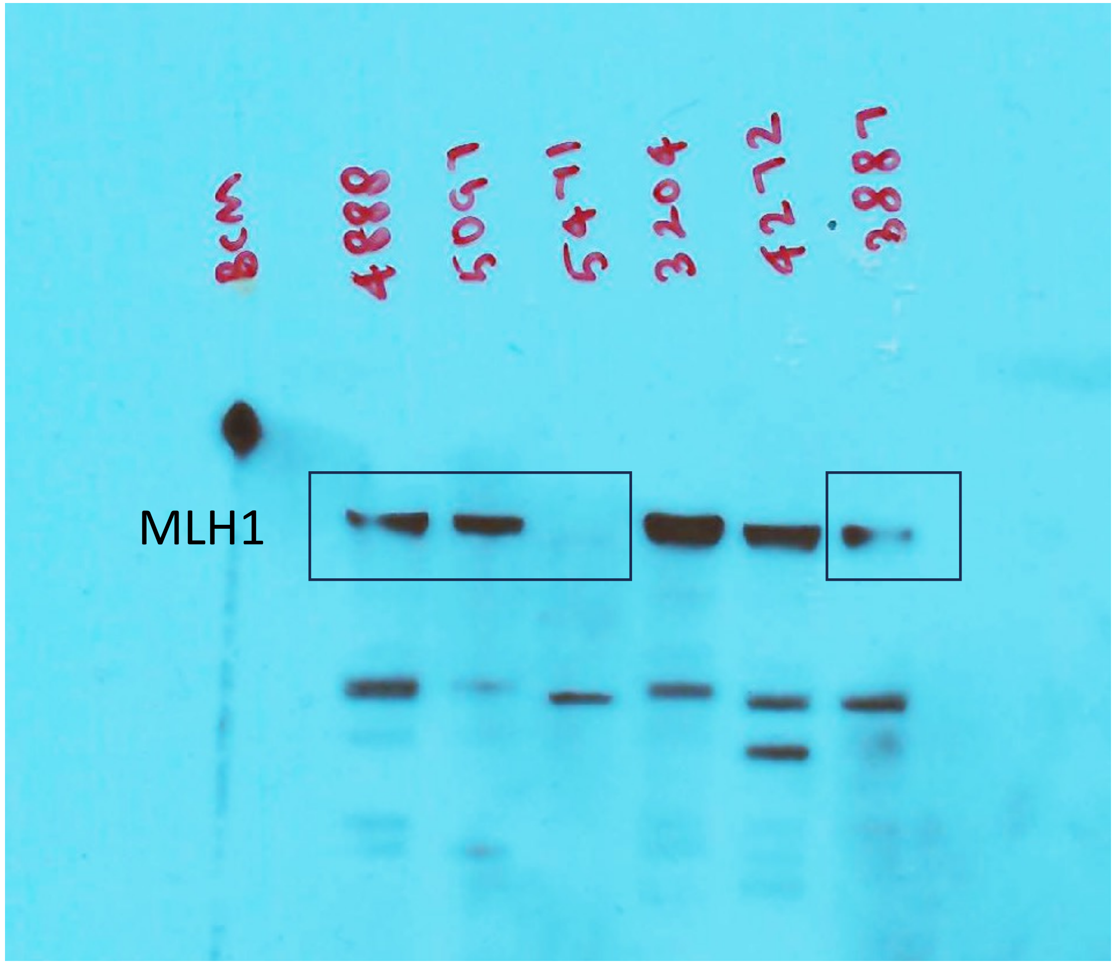

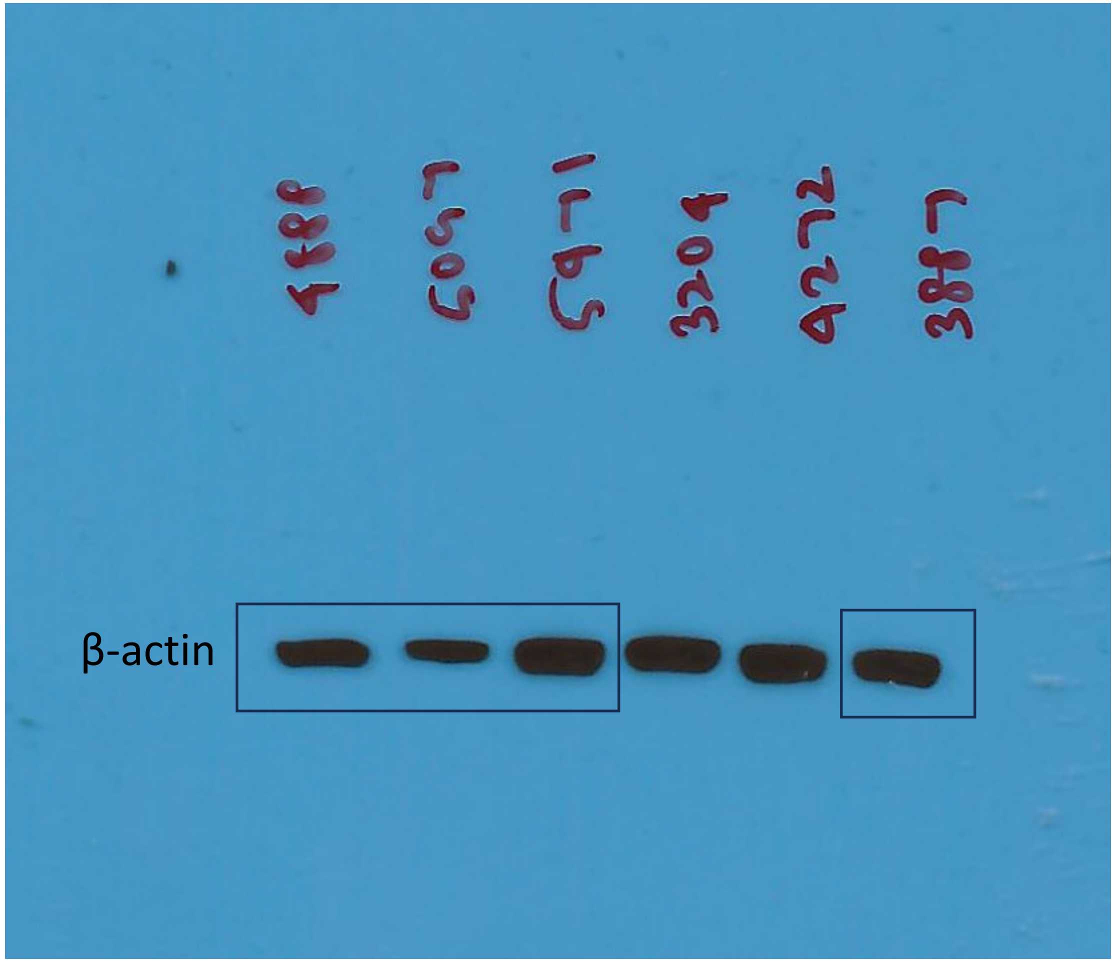

Full unedited blot for Figure 7J

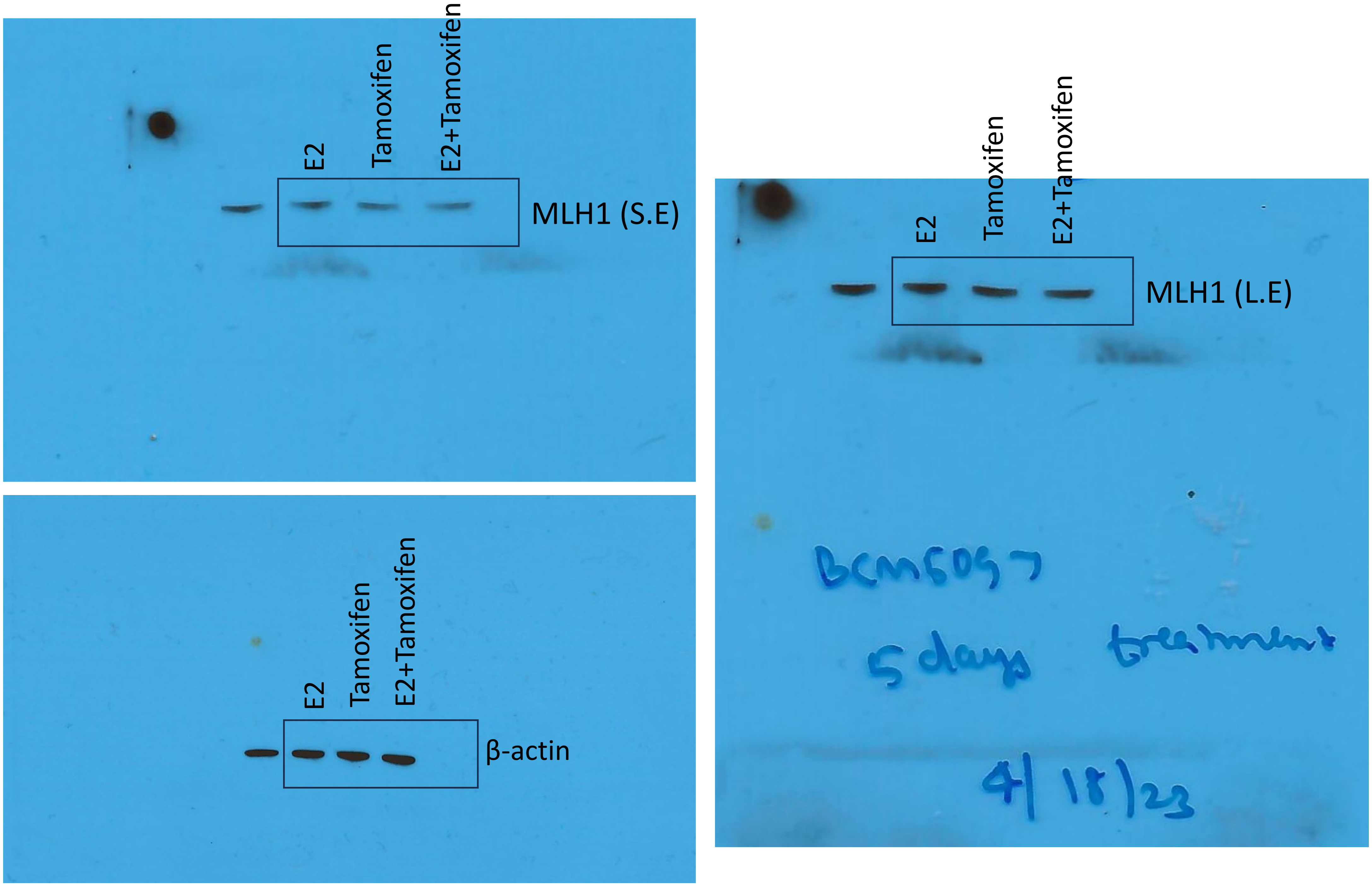

Supplement: Unedited blot and gel images [file jci-134-173718-s070.pdf]
